# The Binding of *Pseudomonas aeruginosa* to Cystic Fibrosis Bronchial Epithelial Model Cells Alters the Composition of the Exosomes They Produce Compared to Healthy Control Cells

**DOI:** 10.3390/ijms25020895

**Published:** 2024-01-11

**Authors:** Víctor Lozano-Iturbe, Noelia Blanco-Agudín, Emma Vázquez-Espinosa, Iván Fernández-Vega, Jesús Merayo-Lloves, Fernando Vazquez, Rosa M. Girón, Luis M. Quirós

**Affiliations:** 1 Department of Functional Biology, University of Oviedo, 33006 Oviedo, Spain; vity921@gmail.com (V.L.-I.); uo257460@uniovi.es (N.B.-A.); fvazquez@uniovi.es (F.V.); 2 Instituto Universitario Fernández-Vega, Fundación de Investigación Oftalmológica, University of Oviedo, 33012 Oviedo, Spain; fernandezvivan@uniovi.es (I.F.-V.); merayojesus@uniovi.es (J.M.-L.); 3 Instituto de Investigación Sanitaria del Principado de Asturias (ISPA), 33011 Oviedo, Spain; 4 Pneumology Service, Institute for Health Research (IP), Hospital Universitario de La Princesa, 28006 Madrid, Spain; evazqueze@hotmail.com; 5 Department of Pathology, Hospital Universitario Central de Asturias, 33011 Oviedo, Spain; 6 Department of Microbiology, Hospital Universitario Central de Asturias, 33011 Oviedo, Spain

**Keywords:** exosomes, cystic fibrosis, extracellular vesicles, miRNAs

## Abstract

Cystic fibrosis (CF) is a genetic disease that causes dehydration of the surface of the airways, increasing lung infections, most frequently caused by *Pseudomonas aeruginosa*. Exosomes are nanovesicles released by cells that play an essential role in intercellular communication, although their role during bacterial infections is not well understood. In this article, we analyze the alterations in exosomes produced by healthy bronchial epithelial and cystic fibrosis cell lines caused by the interaction with *P. aeruginosa*. The proteomic study detected alterations in 30% of the species analyzed. In healthy cells, they mainly involve proteins related to the extracellular matrix, cytoskeleton, and various catabolic enzymes. In CF, proteins related to the cytoskeleton and matrix, in addition to the proteasome. These differences could be related to the inflammatory response. A study of miRNAs detected alterations in 18% of the species analyzed. The prediction of their potential biological targets identified 7149 genes, regulated by up to 7 different miRNAs. The identification of their functions showed that they preferentially affected molecules involved in binding and catalytic activities, although with differences between cell types. In conclusion, this study shows differences in exosomes between CF and healthy cells that could be involved in the response to infection.

## 1. Introduction

Exosomes are nanovesicles released into the extracellular medium by different cell types, both in healthy and pathological conditions, and are characterized mainly by their size, between 30 and 150 nm in diameter [1]. They play an essential role in intercellular communication, a function that depends on their molecular composition, composed mainly of proteins and different types of RNA, including mRNAs and miRNAs [2,3].

The presence of exosomes has been detected in various biological fluids such as blood or urine, and in pleural effusions, sputum, or bronchoalveolar lavages [4], which suggests that these vesicles play important roles in normal lung physiology, contributing to the maintenance of homeostasis through the mediation of cell communication in the airways [5]. Most cell types present in the lung release exosomes, including epithelial cells, endothelial cells, fibroblasts, stem cells, and immune cells, although different studies suggest that epithelial cells are the main source of exosomes in this organ [6].

The main mechanism of control of homeostasis mediated by exosomes is based on their contribution to signaling and intercellular communication [7]. Among other cases analyzed, it has been described how damaged tissues release vesicles capable of sending signals to progenitor cells to modulate their function [8], the modulation of differentiation and metabolism through the transfer of miRNAs [9], the activation of mechanisms for the degradation of organelles or proteins in response to stress situations [10], or the modulation of the activity of immune cells [11].

In addition to their role in physiological control, in recent years much evidence has appeared linking exosomes to pulmonary pathologies, including idiopathic pulmonary fibrosis [12], chronic obstructive pulmonary disease [13], lung cancer [14], or cystic fibrosis (CF) [15]. In the case of infectious pathologies, very recent evidence indicates that, in the upper respiratory tract, mucosal exposure to inhaled pathogens stimulates the secretion of exosomes with antimicrobial activities [16].

CF is a genetic disease caused by the mutation of a gene that encodes a chloride channel located in the membrane of epithelial cells, the CF transmembrane conductance regulator (CFTR) protein. CFTR mutations produce decreased secretion and increased electrolyte absorption, leading to the thickening of the secretions lining the respiratory epithelium. This thickening can lead to airway obstruction, accompanied by inflammation with excessive recruitment of neutrophils and tissue destruction due to the release of hydrolytic enzymes [17]. As a result of the above, CF patients experience an increase in pulmonary bacterial infections [18]. Initial isolation of *Staphylococcus aureus* is common, and its presence, together with other pathogens such as *Haemophilus influenzae*, predisposes to colonization by *Pseudomonas aeruginosa*. Initially, *P. aeruginosa* infection is carried out by non-mucoid organisms, but its persistence in the lungs of CF patients results in alginate-producing mucoid phenotypes [17]. The mucoid phenotype originates from biofilms that protect the microorganism from antimicrobial agents and immune defense, so early treatment can prevent or delay the development of chronic infection [17,18].

CF is a pathology associated with the existence of persistent inflammation, and evidence has been described suggesting that intercellular communication mediated by secreted vesicles may play a role in the airways under inflammatory conditions [5]. Previous studies have reported the fact that exosomes, isolated from both sputum and bronchoalveolar lavage fluid in inflammatory lung disorders, are capable of inducing proinflammatory effects in bronchial epithelial cells, suggesting their contribution to the dissemination of lung inflammation [19]. On the other hand, the analysis of the proteomic content of exosomes isolated from bronchoalveolar lavage fluid from patients with CF compared to patients with asthma (pathology also associated with inflammation but with less severe lung damage) showed a higher content of antioxidant and implicated proteins. in leukocyte chemotaxis, reinforcing its role in propagating inflammation [15].

On the other hand, in the composition of these nanovesicles, along with proteins, lipids, and RNAs, proteoglycans also appear, particularly heparan sulfate proteoglycans (HSPGs). In addition, these molecules are very relevant since they play essential roles in relation to the biogenesis, secretion, and composition of exosomes, as well as in their internalization by the recipient cells and their functional activity in them [20,21]. HSPGs are complex molecules made up of specific proteins to which heparan sulfate (HS) polysaccharide chains are covalently attached. HS is formed by long, unbranched chains made up of glucuronic acid and N-acetyl-glucosamine, modified by a series of chemical reactions that give rise to enormous structural diversity [22]. These molecules interact specifically with a multitude of ligands, acting as regulators of cell physiology [22]. Previous studies in our laboratory have shown that the structure of these molecules is altered in lung epithelial cells used as CF models [23], which is directly related to the regulation of exosome formation, binding, and internalization. of these nanovesicles in the receptor cells, as well as their functional activity.

Different studies suggest that the nanovesicles secreted by bacteria and epithelia seem to play an essential role in the control of homeostasis and the development of pathologies. This has been particularly studied in the intestine, where microbiota-host bidirectional communication does not imply direct cellular contacts, but rather the exchange of nanovesicles that seems to regulate, through effectors, cellular processes [24]. In the case of CF, the previously described structural alteration of HSPGs [25] should influence both exosome synthesis [20,21] and bacterial adherence [23]. Based on these considerations, the objective of this article is to isolate exosomes produced by bronchial epithelial cell lines, both healthy and those with cystic fibrosis, and determine the alterations they experience in response to the interaction with *P. aeruginosa*. The study aims to analyze the protein and miRNA content present in the vesicles, as well as the bioinformatic analysis of the observed alterations, to try to achieve a better understanding of the lung infections associated with the development of the pathology.

## 2. Results

### 2.1. Characterization of Exosomes Derived from Bronchial Epithelial Cell Lines

**Figure 1:**
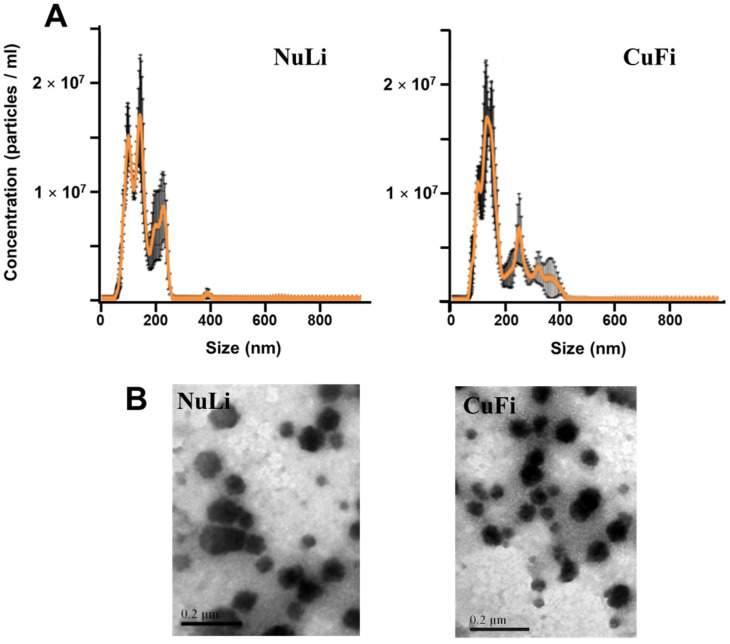
Characterization of extracellular vesicles derived from bronchial epithelial cell lines NuLi and CuFi. (**A**) Vesicle size distribution determined by NTA analysis; left: NuLi cells, right: CuFi cells. The average size per concentration with standard error of the mean is shown. (**B**) TEM images of exosomes from NuLi cells (left panel) and CuFi cells (right panel).

The nanoparticle tracking analysis (NTA) of the nanovesicles isolated from the healthy epithelium cell line (NuLi) showed a size distribution in which two peaks appeared, a main one corresponding to 130 nm and a secondary one corresponding to 100 nm. In both cases, the sizes were within the typical range of exosomes. On the other hand, samples isolated from the CF disease model cell line (CuFi) showed a very similar pattern, although more shifted in favor of the 130 nm peak (Figure 1A). When the samples were analyzed by transmission electron microscopy (TEM), in both cases, vesicles of relatively homogeneous sizes were observed, located at values of 87.2 ± 22.2 nm for NuLi and 71 ± 16 nm for CuFi. (Figure 1B). No relevant changes were observed after the adherence of *P. aeruginosa*.

### 2.2. Proteomic Analysis of Exosomes from Bronchial Epithelial Cell Lines with and without Contact with Pseudomonas aeruginosa

LC-MS/MS-based proteomic analysis identified 1283 different proteins in exosomes isolated from cultures of the NuLi and CuFi cell lines, as well as from those same lines cultured in contact with *P. aeruginosa* (Supplementary Table S1). When the proteins identified in this study were compared with those commonly identified in exosomes isolated from other cell types (http://exocarta.org/exosome_markers_new; accessed on 28 March 2023), it was possible to determine the presence of 16 of the 20 most frequent proteins (80%), and 28 of the 40 most frequent proteins (70%) are considered (Supplementary Table S2). Furthermore, the core proteome of exosomes has recently been described, identifying putative exosomal biomarkers. These markers were classified into classes, and the ubiquitous proteins with higher abundance in exosomes compared to the cells from which they are derived were included in classes I to IV [25]. In this study, all proteins present in these 4 classes could be identified, with the only exception of B2M, which is included in class III (Supplementary Table S3).

**Figure 2:**
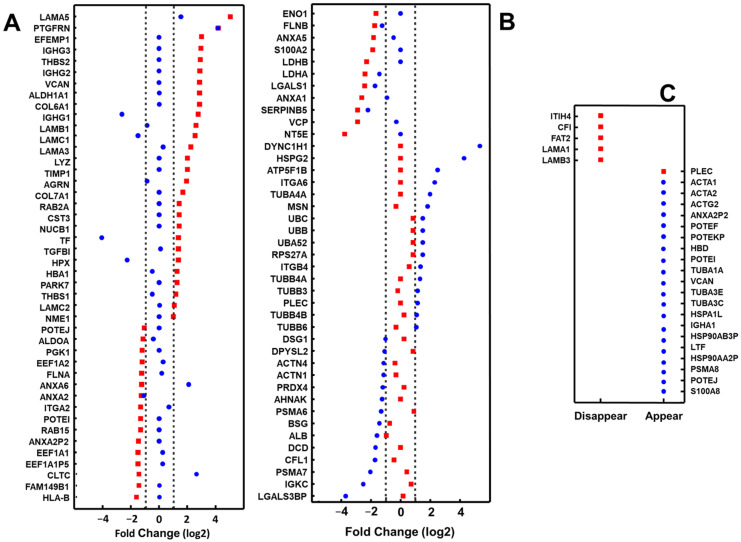
Proteins of exosomes produced by the bronchial epithelial cell lines whose levels are altered after the union of *P. aeruginosa*. (**A**,**B**) Proteins showing upregulation (≥2-fold levels) or downregulation (≤0.5-fold levels). (**C**) Proteins whose detection in exosomes appears or disappears after the attachment of the bacterium. Red squares: NuLi; blue dots: CuFi.

For further analysis, the 179 proteins that appeared represented by at least 10 PSMs were selected (Supplementary Table S4). Different proteins altered their levels ≥2-fold, specifically 22 in the case of NuLi and 16 in the case of CuFi, while another 2, LAMA5 and PTGFRN, did so in both cell lines (Figure 2A,B). On the other hand, the levels of 30 proteins decreased (≤0.5 times) in NuLi, 13 in CuFi, and another 6 (ANXA2, FLNB, LDHA, LGALS1, SERPINB5, ALB) in both cell types. Six proteins showed opposite behaviors depending on the cell line: Fours were overexpressed in NuLi while downregulated in CuFi (IGHG1, LAMC1, TF, HPX), while two (ANXA6 and CLTC) were underexpressed in NuLi while upregulated in CuFi (Figure 2A,B). Furthermore, the binding of bacteria resulted in the loss of detection of 5 proteins present in NuLi exosomes and the detection of 1 new protein in NuLi and 20 in CuFi (Figure 2C).

**Figure 3:**
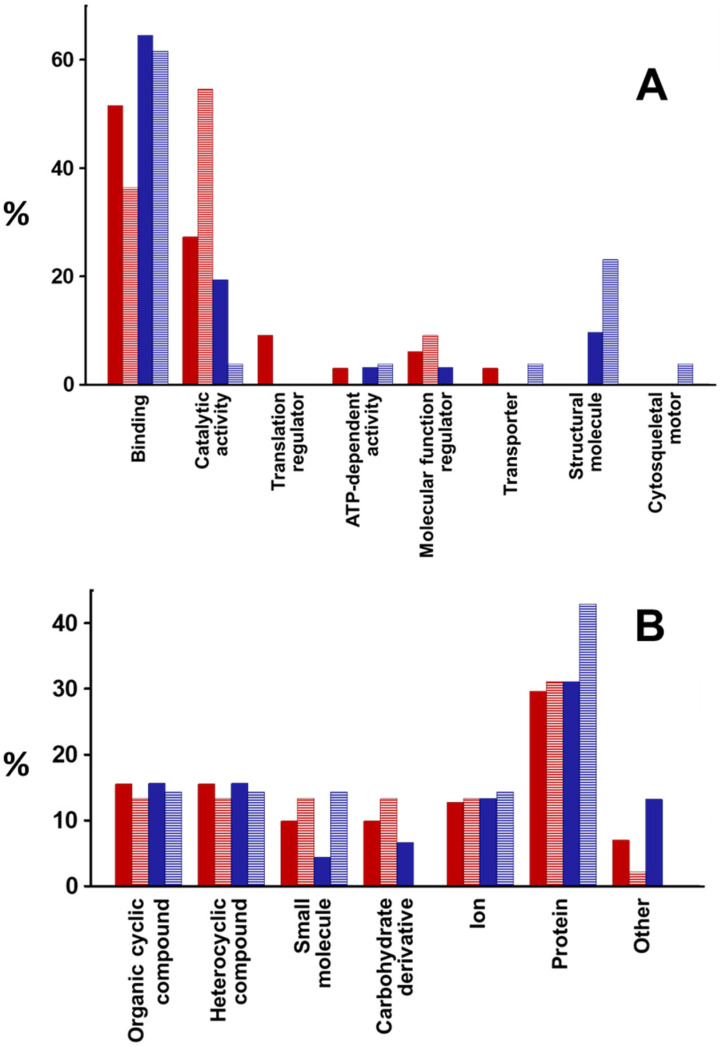
Identification, using the PANTHER classification system, of the molecular functions of the proteins whose relative abundance levels in exosomes were altered by the adherence of *P. aeruginosa*. (**A**) Functions of proteins. (**B**) Identification of types of ligands within the group of proteins involved in binding. Red bars: NuLi; blue bars: CuFi; solid bars: proteins with high levels; striped bars: proteins with decreased levels.

The function of the proteins whose levels were altered in the exosomes produced by epithelial cells after the adherence of *P. aeruginosa* was carried out using the PANTHER classification system (http://pantherdb.org/; accessed on 4 May 2023). It was possible to assign functions to 80.5% of the overexpressed and 30% of the underexpressed in NuLi, as well as to 61 and 87% of the overexpressed and underexpressed in CuFi, respectively. The majority of functions were related to binding, followed by catalytic activities, except for those underexpressed in NuLi, where this second category was the majority. The rest of the functions represented much more limited percentages (Figure 3A). On the other hand, when the analysis was limited to the proteins involved in binding, it was found that in all cases, they were mostly related to binding to other proteins (Figure 3B).

**Figure 4:**
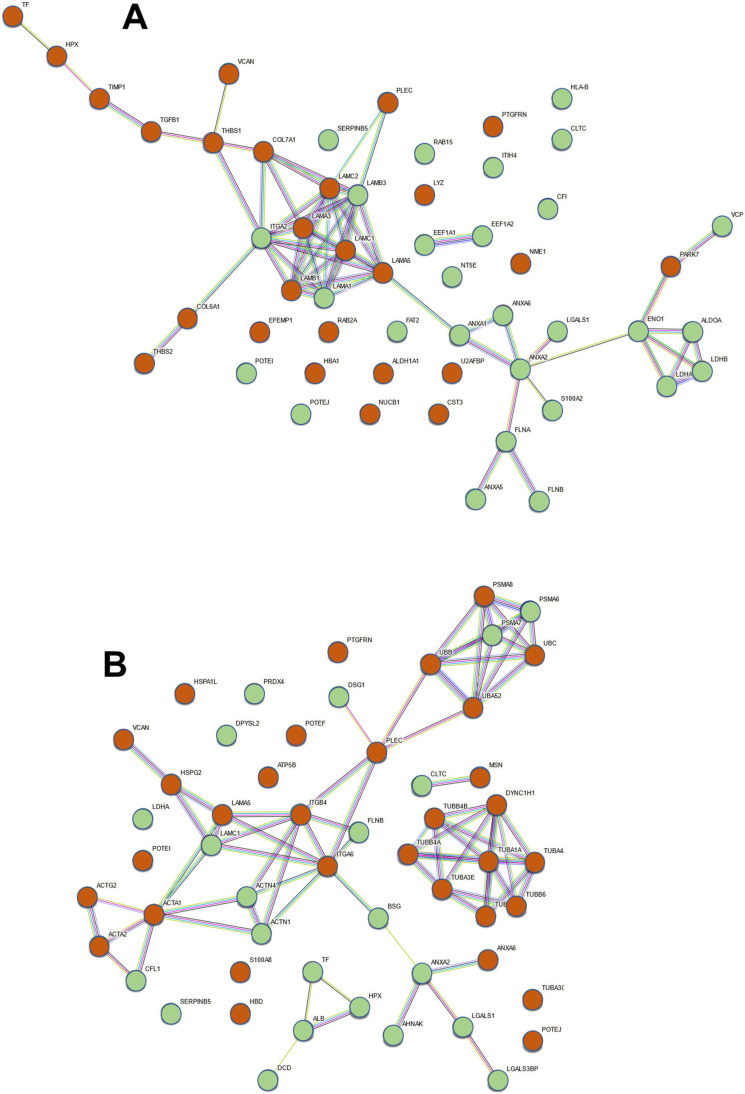
Functional association network of the proteins whose relative abundance levels in exosomes were altered by the adherence of *P. aeruginosa*. The STRING program was used to model the functional association of proteins whose expression is altered in exosomes of (**A**) NuLi cells and (**B**) CuFi cells. Brown nodes represent proteins whose levels are increased, and green nodes represent those whose levels are decreased.

To identify the existence of functional relationships between the proteins whose levels appeared altered in the exosomes after the binding of the bacteria, we carried out a functional association network study using the STRING program (https://string-db.org/; accessed on 24 May 2023), and the results are shown in Figure 4. The association network generated by the altered proteins in NuLi exosomes showed a core of intense interactions formed mainly by different laminin chains, mostly corresponding to proteins whose level is increased in exosomes (LAMC1, LAMC2, LAMA5, LAMB1, LAMA3), with some whose level decreases (LAMA1, LAMB3). In direct relation to this nucleus, there are molecules mainly responsible for interactions with the extracellular matrix whose levels increased (PLEC, COL7A1, COL6A1, THBS1, THBS2, VCAN), and integrin alpha-2 (ITGA2), which acts among other functions as a receptor for laminin, whose level decreased. A second core of looser interactions was centered on annexin A2 (ANXA2) and was made up of molecules whose levels, in all cases, decreased in the exosomes produced by NuLI. More heterogeneous molecules were included in this group, including different regulatory proteins (ANXA2, ANXA1, ANXA6, LGALS1, S100A2), filamins (FLA, FLNB), and a group of catabolic enzymes (ENO1, ALDOA, LDHA, LDHB). In the case of CuFi, the network showed association groups formed mainly by proteins related to the cytoskeleton. On the one hand, the levels of different tubulin chains or functionally related proteins (TUBA1A, TUBA3E, TUBA4A, TUBB3, TUBB4A, TUBB4B, TUBB6, DYNC1H1) appeared increased. On the other hand, there was an extensive association group that included proteins of the actin family (ACTA1, ACTA2, ACTG2), whose levels were increased, or related to their dynamics and function (CLF1, ACTN1, ACTN4), whose levels decreased. These molecules were related to extracellular matrix proteoglycans (HSPG2, VCAN) and laminins (LAMC1, LAMA5) or receptor integrins of the latter (ITGA6, ITGB4), as well as molecules that serve as connections between different elements of the cytoskeleton and the cell surface (FLNE, PLEC). In connection with the above, an association network appeared between proteins related to the proteasome (PSMA6, PSMA7, and PSMA8) and ubiquitin (UBA52, UBB, and UBC).

### 2.3. miRNA Profile Analysis of Exosomes from Bronchial Epithelial Cell Lines with and without Contact with Pseudomonas aeruginosa

**Figure 5:**
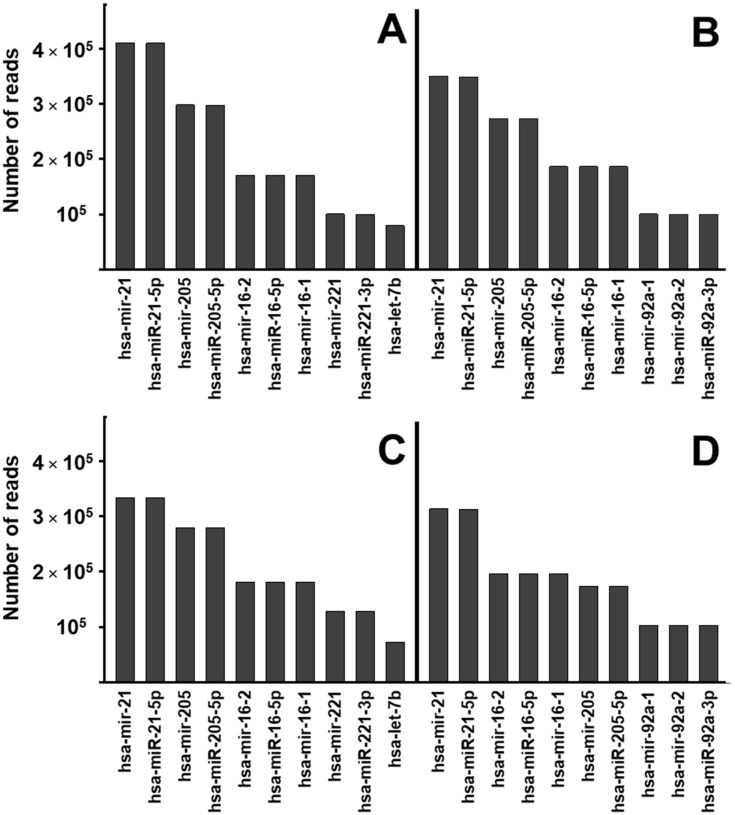
Expression of the 10 most abundant miRNAs in bronchial epithelial cell lines with and without contact with *P. aeruginosa*. (**A**) NuLi cells; (**B**) CuFi cells; (**C**) NuLi cells after the union of *P. aeruginosa*; (**D**) CuFi cells after the union of *P. aeruginosa*.

Using Next Generation Sequencing, we were able to detect several hundred different miRNAs, of which 243 were considered the most representative since they were present in at least 1000 copies (Supplementary Table S5). The 10 most abundant species were matched in exosomes isolated from NuLi and CuFi cells (Figure 5A,B). However, contact with *P. aeruginosa* produced differential alterations in these species between both cell lines. In NuLi, the 7 most abundant species were maintained, but mir-221, miR-2221-3p, and let-7b, located in the eighth to tenth positions, were replaced by mir-92a-1, mir 92a-2, and miR-92a-3P (Figure 5C). The changes were greater in CuFi, where miRNAs 8 to 10 were also replaced by the same species as in NuLi, but, in addition, mir-205 and mir-205-5p went from being the third and fourth most abundant species to positions 6th and 7th, advancing mir-16-2, mir-16-5p, and mir-16-1 to the 3rd, 4th, and 5th positions, respectively (Figure 5D).

**Figure 6:**
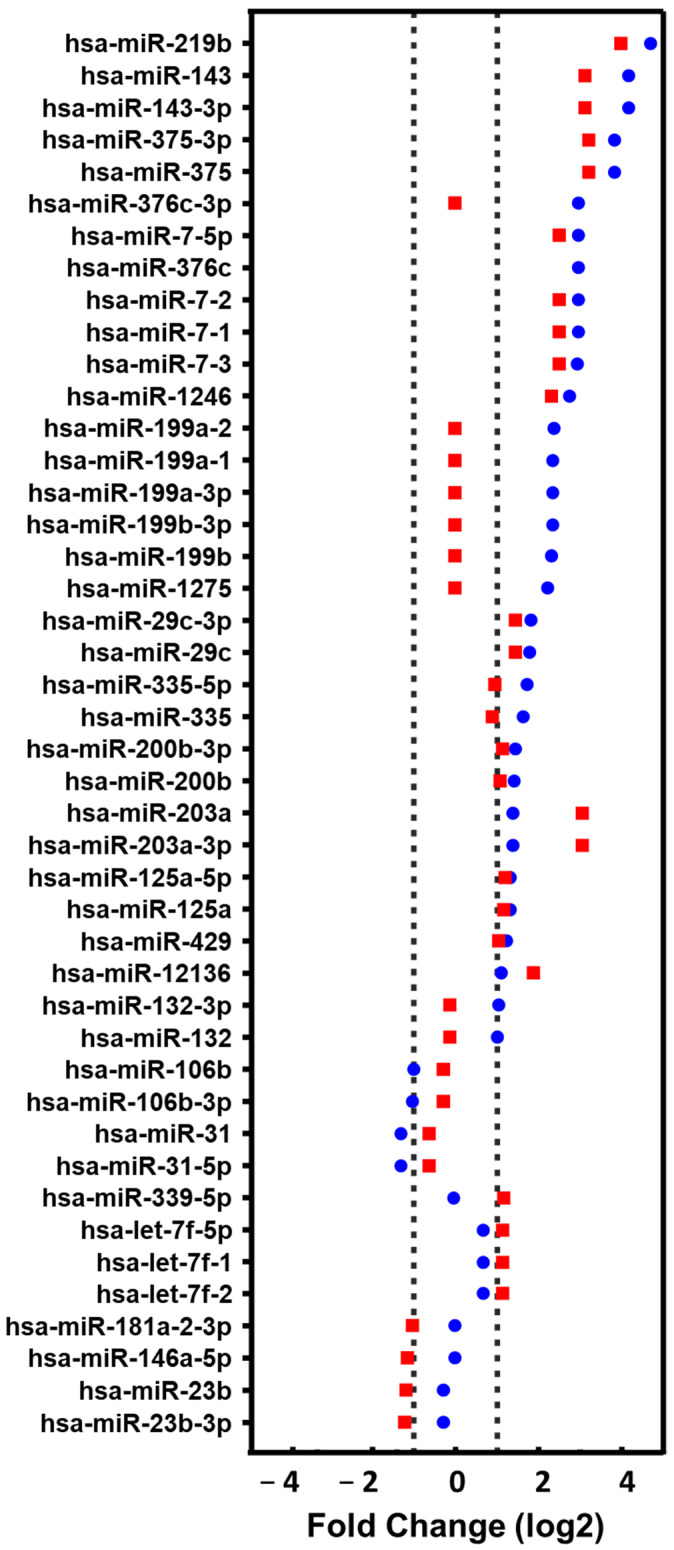
miRNAs from exosomes produced by bronchial epithelial cell lines whose levels show upregulation (≥2-fold levels) or downregulation (≤0.5-fold levels) upon *P. aeruginosa* binding. Red squares: NuLi; blue dots: CuFi.

Of the 243 miRNAs analyzed, *P. aeruginosa* binding to bronchial epithelial cells produced levels of at least 2-fold upregulation in 36 species (14.8%) and downregulation levels of at least 50% in 8 species (3.2%). The overexpression of 4 species was detected only in NuLi and that of 12 in CuFi, while 20 species were overexpressed in both cell lines. On the other hand, 4 species appeared with their levels decreased in NuLi and another 4 in CuFi (Figure 6).

The bioinformatic analysis of the miRNAs whose levels were altered allowed us to identify numerous genes potentially regulated by them. In the case of species whose levels rise in both NuLi and CuFi after contact with the bacterium, 2802 potential targets were identified, of which 3 were regulated by 7 different miRNAs, 8 by 6, 28 by 5, 42 by 4, 266 by 3, 838 by 2, and 1617 by a single miRNA. For the miRNAs whose levels only increased in NuLi, 1280 targets were detected, of which 9 could be regulated by 3 different species, 183 by 2, and 1088 by 1. On the other hand, those that increased only in CuFi presented 1532 possible targets, 1 potentially regulated by 5 miRNAs, 7 by 4, 37 by 3, 163 by 2, and 1324 by 1. Regarding the species whose levels appeared decreased, in the case of NuLi 794, potential targets were detected, with 1 of them regulated by 3 miRNAs, 37 by 2, and 756 by 1, while in CuFi 741 species were detected, with 14 regulated by 2 miRNAs and 727 by 1 (Supplementary Table S6).

The identification of the functions of the proteins whose genes are potentially regulated by the miRNAs whose levels were altered showed, in all cases, that they preferentially affect molecules involved in binding, followed by catalytic activities (Supplementary Figure S1). However, differences were observed in the less frequently affected categories. In the case of the proteins regulated by miRNAs whose levels were increased in exosomes produced by both cell types, genes encoding transcription regulator activities were affected, followed by molecular function regulators, transporter, and molecular transducer activities. In the case of miRNA-regulated proteins that were exclusively increased in either NuLi or CuFi, as well as those that were decreased in CuFi, the predicted patterns were virtually identical, potentially affecting regulatory genes of structural molecules, followed to a lesser extent by molecular function regulators, molecular adapter, and translation regulator activities. On the other hand, the genes potentially regulated by miRNAs whose levels appeared decreased in the exosomes produced by NuLI followed a different pattern to the previous ones, appearing more affected by molecular transducer activities, followed by molecular adaptor, lipoprotein receptor, and transcription regulation activities (Supplementary Figure S1).

## 3. Discussion

All cell types in the respiratory system can secrete extracellular vesicles. These vesicles can be detected in the bronchoalveolar lavage fluid, and the epithelium lining the airways appears to be their main source [26]. These vesicles allow the delivery of specific molecular species, mainly proteins and different types of RNA, allowing them to play key roles in tissue physiology and cellular communication [3,27,28]. Each type of cell varies the molecular composition of exosomes depending on their physiological and pathological state, so these nanovesicles have potential applications as diagnostic and therapeutic agents. Among the functions of exosomes is the control of inflammation signals and the response to stress, so, in the case of the lung epithelium, the production of exosomes can be influenced by environmental factors, such as cigarette smoke [13], or microbial infections [29].

In the case of CF, the dehydration produced by the alteration in CFTR is the main cause of impaired cilia functioning and mucociliary clearance, which results in bacteria not being cleared from the airway. There is evidence of the release of vesicles by epithelial cells in CF, and their possible usefulness has been proposed to identify the state of the pathology [30]. Given that *P. aeruginosa* is the most common bacteria that affects CF patients [31], we have addressed the study of the alterations in the molecular composition of exosomes induced by the infection of this microorganism in an in vitro model of cystic fibrosis. To carry it out, we used the bronchial epithelial cell line NuLi-1 as a model of healthy epithelium and CuFi-1, which carries the Δ508/Δ508 mutation, as a model of CF.

The analysis of the exosomes isolated from both cell lines using NTA showed an average particle size of 130 nm, with a second peak located at 100 nm, more evident in the NuLi samples, sizes that fit perfectly into the expected range for exosomes. TEM visualization of the vesicles confirmed the results, although their sizes appeared slightly smaller, between 87 and 71 nm. The appearance of bimodal distributions of vesicles, with the presence of a second peak corresponding to larger sizes, is consistent with previous descriptions and has been assigned to the presence of low levels of larger particles, susceptible to altering the weighted size distribution due to their brighter light dispersion [32]. On the other hand, previous studies on the physical characterization of exosomes derived from airway epithelium have suggested that the smaller size observed in TEM compared to NTA may be due to mucin decoration on the surface of these nanoparticles [33].

The proteomic analysis of the isolated exosomes allowed the identification of 1283 proteins, among which 80% of the 20 most common proteins described in exosomes produced by other cell types and 70% of the 40 most frequent were found, according to the database ExoCarta, confirming the nature of the isolated vesicles. However, it has recently been described that frequently, biomarkers of exosomes, including the tetraspanin CD9, CD63, and CD81, are heterogeneous and do not exhibit universal utility across different cell types. Unbiased quantitative analysis has identified putative exosomal biomarkers that have been grouped into classes on the basis of their measured abundance, with the first four classes being highly abundant proteins in exosomes [25]. Class I is formed solely by syntenin-1, which has been consistently identified in exosomes produced by different cell lines. The potential use of this molecule as a supposed universal biomarker has been proposed, and in this study, it has been possible to identify it in the two cell lines used, both in the absence and presence of Pseudomonas. Similarly, all the proteins that constitute classes II (SLC1A5, SLC3A2, GNB1, CLTC), III (CD47, GNB2, ITGB1, BSG), and IV (ATP1A1, RAP1B, GNAI3) could be detected in the exosomes of NuLi and CuFI, the only exception being beta-2-microglobulin (B2M), integrated into class III, which did not show detectable values. These data once again confirm the nature of the nanovesicles purified in this study.

Among the detected proteins, the 179 that presented the most consistent levels, represented by at least 10 PSM, were selected for analysis. The binding of *P. aeruginosa* altered the levels of 54 proteins in the case of NuLi and 55 in that of CuFi. However, alterations that analogously affected the same molecules in both cell types only represented 15% of the cases, most of them being specific to each cell line. Previous studies on comparative proteomics of respiratory exosomes in CF detected 14 proteins with altered expression in the pathology, none of which coincided with those detected in this work (15). However, the previous work was carried out using bronchoalveolar fluid and comparing CF with primary ciliary dyskinesia and asthma, so the conditions analyzed were very different from those used in this work.

In order to assign functional significance to the alterations observed in exosomal proteins upon *P. aeruginosa* binding, the PANTHER classification system was used, performing separate analyses for proteins that were overexpressed and underexpressed. Among the overexpressed ones, binding activities constituted the main functional group (51% and 65% in NuLi and CuFi, respectively), followed by catalytic activities. However, among the proteins whose levels decreased, differences were found between both cell lines. Those involved in catalytic activities were the main group in NuLi, followed by binding activities, while in CuFi the group of molecules related to binding was the most abundant, with those involved in catalytic activities representing a minor amount. When the same type of focused analysis was carried out on the proteins involved in binding to determine potential ligands, it was found that in all cases, they were mainly related to binding to other proteins.

Interestingly, in the CuFi exosomes, a set of proteins were detected after the binding of *P. aeruginosa* that were absent in the controls, and among them, several pseudogenes were identified (ANXA2P2, POTEKP, HSP90AB3P, and HSP90AA2P). Pseudogenes are copies of genes that are assumed to have no biological function. However, recent studies have detected hundreds of pseudogene transcripts that appear expressed in tissue-specific patterns, as well as the detection of numerous peptides encoded by them [34]. It has been described that a smaller percentage of these proteins have biological functions (15%), although many seem to come from accidental translations of pseudogene transcripts [35]. Although several pseudogenes have been shown to be involved in pathologies, which could be related to their detection in CF model cells, it is interesting to emphasize that one of the functions of exosomes is to remove excess and unnecessary constituents from cells to maintain cellular homeostasis [36].

We carried out an association network study using the STRING program to identify the existence of functional relationships between the proteins whose levels appeared altered in exosomes after the binding of *P. aeruginosa*. In the case of NuLi, two sets of related proteins could be visualized. The first of them included different isoforms of the laminin chains, five of which were increased and two were decreased, as well as the laminin receptor integrin alpha-2 (ITGA2). This set also showed a certain relationship with molecules mainly involved in interactions with the extracellular matrix. The second group presented fewer compact relationships and included subgroups composed mainly of annexins, filamins, and certain catabolic enzymes. On the other hand, in CuFi, a network composed of laminins, laminin receptors, and extracellular matrix (ECM) proteoglycans was also detected, although to a lesser extent and related to actin, including structural molecules, and to dynamics and function. Furthermore, two other relevant groups could be observed, the first formed by seven members of the tubulin family together with functionally related dynein and the second by proteins related to the proteasome and ubiquitin.

It should be noted that the association networks detected in NuLi mainly include ECM proteins, particularly laminins, membrane anchoring receptors, proteins with relationships with the cytoskeleton (annexins and filamins, among others), as well as various catabolic enzymes. This allows us to propose possible functional relationships since the ECM acts on the structure and dynamics of the cytoskeleton, influencing cell behavior and dynamics [37]. On the other hand, several of these elements are related to inflammation and the immune response. This is the case of proteoglycans, or laminin, capable of acting by regulating signaling pathways [38]. Annexins are reversibly associated with the cytoskeleton or with proteins that mediate the interaction between the cell and the ECM, and their alterations cause changes in the inflammatory response [39], while filamins also interact with the cytoskeleton together with other ligands, conditioning cellular function [40]. On the other hand, alterations in certain metabolic enzymes have also been related to the regulation of inflammation, and, in fact, several of them have evolved moonlighting functions with roles in the immune response totally independent of their conventional enzymatic activities [41]. In CuFi, alterations were also observed in the protein levels of the ECM and membrane receptors, but especially in the cytoskeleton, involving actin and tubulin, which again could be related to the inflammatory response to the presence of microorganisms [42,43], although the results would suggest the existence of differences with respect to healthy cells. Furthermore, alterations in the proteasome and ubiquitin could have some relationship with multiple factors, among which could be their described involvement in inflammation [44], or the translation of species derived from the pseudogenes indicated above.

Along with proteins, an essential component of exosomes is the various types of non-coding RNAs, particularly miRNAs. Their importance is such that these molecules are thought to be the major contributors to the molecular events occurring in the recipient cell [45]. In the lung, miRNAs have been described to play relevant roles, both as regulators of development and in the maintenance of homeostasis. In fact, different studies have detected important changes in the levels of certain miRNAs during fetal lung development [46], while their levels remain relatively constant in the developed lung [47]. However, the expression of miRNAs is altered in pathological states, such as asthma, chronic obstructive pulmonary disease, pulmonary hypertension, or cancer [48]. Also, in the case of CF, the study of different patient samples, including bronchial brushings or sputum, has described the alteration of numerous miRNAs in the bronchial epithelium [49,50].

In this study, several hundred different miRNAs were detected, of which those present in at least 1000 copies were selected, resulting in 243 species on which subsequent analysis was carried out. Binding of *P. aeruginosa* to bronchial epithelial cells resulted in altered levels of 44 species (18%), of which 36 experienced upregulation and eight experienced downregulation. Previous studies using bronchial brushing have detected the alteration of 93 miRNAs in CF, with 57 of them down-regulated and 36 up-regulated [51], and eight of these species also showed alterations associated with bacterial adhesion in this study. Of the miRNAs previously described as up-regulated in CF, two increased their levels because of the binding of *P. aeruginosa*: miR-143, both in NuLi and CuFi, and miR-199a-3p, only in CuFi. In addition, six molecules described as downregulated in CF altered their levels: miR-29c, miR200b, miR-125a-5p, and miR-429 increased in both cell lines, while miR-31 decreased only in CuFi and miR-23b at NuLi. Interestingly, miR-126, whose downregulation has been described in CF and has been associated with toll-like receptors hyporesponsive state, which could be important at times of infective exacerbations [51], was detectable in our study but did not experience any alterations.

Genes potentially regulated by miRNA species that showed altered expression levels could be tracked by means of bioinformatics analysis. 7149 potential targets were detected, of which 5512 were susceptible to being controlled by a single miRNA species, 1235 by 2, 313 by 3, 49 by 4, 29 by 5, 8 by 6, and 3 by 7. The identification of the functions of the potential targets of miRNAs, grouping them based on their over- or under-expression in each cell model, showed, in all cases, that they preferentially affect molecules involved in binding, followed by catalytic activities. However, differences were observed in the less frequently affected categories. The potential targets of the species overexpressed in both NuLi and CuFi, as well as those underexpressed in CuFi, showed very similar patterns, potentially affecting regulatory genes of structural molecules, followed to a lesser extent by molecular function regulators, molecular adapters, and translation regulator activities. However, targets potentially regulated by overexpressed species in both cell types, as well as those whose levels are decreased in NuLi, showed different patterns. In the former, the targets included genes encoding transcription regulatory activities, followed by molecular function regulators, transporter, and molecular transducer activities. In the latter, molecular transducer activities were most affected, followed by molecular adapters, lipoprotein receptors, and transcription regulation activities. In this sense, previous studies on miRNAs identified in the pulmonary pathways have proposed roles related to the host-pathogen relationship [52], inflammation, and the immune response [49].

## 4. Materials and Methods

### 4.1. Cell Lines, Bacterial Strains and Culture Conditions

The bronchial epithelial cell lines used in this work were NuLi-1 (ATCC^®^ CRL-4011), as a model of healthy epithelium, and CuFi-1 (ATCC^®^ CRL-4013™), which carries the mutation Δ508/Δ508 and may act as a CF airway disease model [53] (ATCC, Manassas, VA, USA). The two lines were grown in BEGM™ supplemented with SingleQuots™ Kit (Lonza, Walkersville, MD, USA) at 37 °C in a 5% CO_2_ atmosphere.

A clinical isolate of *P. aeruginosa* from the Central University Hospital of Asturias was used. The bacteria were cultured in brain-heart infusion broth (ThermoFischer, Waltham, MA, USA) at 37 °C in a shaking incubator.

### 4.2. Bacterial Adhesion

*P. aeruginosa* cultures were treated with gentamicin at 500 μg/mL for 4 h, after which the absence of viable microorganisms was confirmed by growth in solid medium. The bacterial suspensions were centrifuged, washed with PBS, and resuspended in PBS to an A_600_ of 0.5.

Adherence of *P. aeruginosa* to epithelial cell monolayers was carried out on cells grown to 50–60% confluence in 90 mm diameter culture dishes. The media was aspirated, and the cells were washed twice with PBS and then blocked with 10% fetal bovine serum in PBS for 1 h at 37 °C in a 5% CO_2_ atmosphere. After further washing with PBS, 2 mL of bacteria in 8 mL of cell culture media was added, and the mixture was incubated for 1 h at 37 °C and 5% CO_2_. Next, the wells were rinsed twice with 500 µL of PBS to remove any unbound bacteria, and the cells were incubated in the culture medium to obtain exosomes.

### 4.3. Exosome Purification

For the purification of the exosomes, plates containing cells grown to a confluence of between 50 and 60% were used, with the culture medium being eliminated and replaced by an equivalent volume where the FBS was replaced by an exosome-depleted FBS (Gibco, Waltham, MA, USA). After 72 h of incubation, the culture medium was collected, and the exosomes were obtained. For the characterization of the nanovesicles by nanoparticle tracking analysis (NTA) or electron microscopy, the exosomes were purified using the total exosome isolation from cell culture media kit (Invitrogen, Waltham, MA, USA), following the manufacturer’s instructions. The exosomes destined for peptide analysis by LC-MS/MS were purified using Exo-spinTM mini columns (CELL guidance systems, Cambridge, UK) following the manufacturer’s specifications; in this case, the precipitation step prior to addition to the column was replaced by ultracentrifugation at 110,000× *g* for 2.5 h to avoid the precipitant interfering with the mass spectrometric analysis. For the isolation of total RNA, the ExoQuick-TC (System Biosciences, Palo Alto, CA, USA) was used, according to the manufacturer’s instructions. Once purified, the exosomes were quantified by determining the protein concentration with the Pierce BCA Protein Assay Kit (Thermo Scientific, Waltham, MA, USA), following the manufacturer’s instructions.

### 4.4. NanoSight Particle Size Analysis Using Dynamic Light Scattering

A 1:100 dilution of the exosomes was prepared in PBS, from which 1:10 and 1:100 dilutions were subsequently obtained using the same buffer. The dilutions were injected into a Malvern Panalytical NanoSight LM10 instrument, starting with the one with the lowest concentration, until the one that provided a number of particles between 21 and 100 in the observed field was identified. The analysis was carried out with a 405 nm laser, and the light scattered by the particles was analyzed using the NTA analysis software 3.1 (Nanosight, Westborough, MA, USA) to determine the size of the particles and their concentration.

### 4.5. Transmission Electron Microscopy (TEM)

Twenty microliters of the purified exosomes was adsorbed onto a carbon grid for 2 min. After drying off the excess sample, it was stained by depositing 50 microliters of 2% phosphotungstic acid on the grid for 1 min and allowing the sample to dry for 10 min. The samples were examined in a JEOL JEM 2000 EX II electron microscope (JEOL, Tokyo, Japan) at 120 Kv.

### 4.6. Proteomic Analysis

The exosome suspension was applied to a standard SDS-PAGE gel and allowed to penetrate 1 cm into the resolving gel. The protein mixture was stained with Coomassie, extracted, and cut into 1 mm^2^ fragments, which were deposited in 96-well plates and processed in a Proteineer DP (Bruker Daltonics, Bremen, Germany), using a digestion protocol based on Schevchenko et al. [54] with minor variations as previously described [55]. The finally obtained tryptic-eluted peptides were dried by fast vacuum centrifugation.

A 1 µg aliquot of each digested sample was subjected to 1D-nano LC ESI-MSMS analysis using a nano liquid chromatography system (Eksigent Technologies nanoLC Ultra 1D Plus, SCIEX, Foster City, CA, USA) coupled to a high-speed Triple TOF 5600 mass spectrometer (SCIEX, Foster City, CA, USA) with a Nanospray III source. The analytical column used was a silica-based reversed-phase Acquity UPLC^®^ M-Class Peptide BEH C18 Column, 75 µm × 150 mm, 1.7 µm particle size, and 130 Å pore size (Waters, Milford, MA, USA). The trap column was a C18 Acclaim PepMapTM 100 (Thermo Scientific) column of 100 µm × 2 cm, 5 µm particle diameter, 100 Å pore size, on-line switched with the analytical column. The loading pump delivered a solution of 0.1% formic acid in water at 2 µL/min. The nano-pump provided a flow rate of 250 nL/min and was operated under gradient elution conditions. Peptides were separated using a 150 min gradient ranging from 2% to 90% in mobile phase B (mobile phase A: 2% acetonitrile, 0.1% formic acid; mobile phase B: 100% acetonitrile, 0.1% formic acid). The injection volume was 5 µL.

Data acquisition was performed with a TripleTOF 5600 System (SCIEX, Foster City, CA, USA), using an ion spray voltage floating (ISVF) of 2300 V, curtain gas (CUR) of 35, interface heater temperature (IHT) of 150, ion source gas 1 (GS1) of 25, and declustering potential (DP) of 100 V. All data were analyzed using information-dependent acquisition (IDA) mode with Analyst TF 1.7 software (SCIEX, Foster City, CA, USA). For IDA parameters, a 0.25 s MS survey scan in the mass range of 350–1250 Da was followed by 35 MS/MS scans of 100 ms in the mass range of 100–1800 (total cycle time: 4 s). Switching criteria were set to ions greater than a mass-to-charge ratio (*m*/*z*) of 350 and smaller than an *m*/*z* of 1250 with a charge state of 2–5 and an abundance threshold of more than 90 counts (cps). Former target ions were excluded for 15 s. The IDA rolling collision energy (CE) parameters script was used to automatically control the CE.

### 4.7. Proteomics Data Analysis

The mass spectrometry data obtained were processed using PeakView v2.2 Software (SCIEX) and exported as mgf files, which were searched using Mascot Server v2.6.0 (Matrix Science, London, UK) against the Homo sapiens protein database from UniProt.

Search parameters were set as follows: Enzyme, trypsin; allowed missed cleavages, 2; carbamidomethyl (C) as fixed modification and acetyl (protein N-term), pyrrolidone from E, pyrrolidone from Q and oxidation (M) as variable modifications. Peptide mass tolerance was set to ±25 ppm for precursors and 0.05 Da for fragment masses. The confidence interval for protein identification was set to ≥95% (*p* < 0.05), and only peptides with an individual ion score above the 1% false discovery rate (FDR) at the spectra level were considered correctly identified.

Among the proteins identified, those that presented a more consistent expression were selected, that is, 10 PSMs (peptide spectrum matches). Proteins whose level differences between samples were ≥2-fold (upregulation) or ≤0.5-fold (downregulation) were considered altered.

The functional classification of proteins according to family, molecular function, biological process, and pathway was carried out using the PANTHER (protein analysis through evolutionary relationships) classification system (http://pantherdb.org/; accessed on 4 May 2023) [56]. Protein–protein interaction networks were generated using the STRING database program (https://string-db.org/; accessed on 24 May 2023) [57], using a high confidence (0.700) as the minimum required interaction score in all cases.

### 4.8. Small RNA Next Generation Sequencing (NGS)

The exosome samples were processed for total RNA isolation using the SeraMir Exosome RNA Purification Column kit (System Biosciences, Palo Alto, CA, USA) according to the manufacturer’s instructions. For each sample, 1 µL of the final RNA eluate was used for measurement of small RNA concentration by the Agilent Bioanalyzer Small RNA Assay using the Bioanalyzer 2100 Expert instrument (Agilent Technologies, Santa Clara, CA, USA).

Small RNA libraries were constructed with the CleanTag Small RNA Library Preparation Kit (TriLink, San Diego, CA, USA) according to the manufacturer’s protocol. The final purified library was quantified with High-Sensitivity DNA Reagents (Agilent Technologies, Santa Clara, CA, USA) and High-Sensitivity DNA Chips (Agilent Technologies). The libraries were pooled, and the 140 bp to 300 bp region was size selected on an 8% TBE gel (Invitrogen, Eugene, OR, USA). The size of the selected library was quantified with High-Sensitivity DNA 1000 Screen Tape (Agilent Technologies), High-Sensitivity D1000 reagents (Agilent Technologies), and the TailorMix HT1 qPCR assay (SeqMatic, Fremont, CA, USA), followed by a NextSeq High Output single-end sequencing run at SR75 using the NextSeq 500/550 High Output v2 Kit (Illumina, San Diego, CA, USA) according to the manufacturer’s instructions.

### 4.9. Differential RNA Expression and Bioinformatics Analysis

Among the identified RNAs, those that were most consistently expressed (>1000 copies) were selected, and those with cut-off values of ≥2-fold (up-regulation) or ≤0.5-fold (down-regulation) between samples were considered differentially expressed.

To identify the proteins regulated by the significantly altered miRNAs, biological targets of miRNAs were predicted by searching for the presence of conserved 8 mer and 7 mer sites that matched the seed region of each miRNA using TargetScanHuman 5.2 (http://www.targetscan.org/vert_50/; accessed on 15 July 2023) [58,59,60] and selecting the targets with scores higher than 80 out of 100. Subsequently, the results of the previous selection were analyzed using the online database for miRNA target prediction and functional annotations, miRDB (http://mirdb.org/; accessed on 21 July 2023) [61,62], selecting the matching results from both tools.

## 5. Conclusions

In conclusion, the adherence of *P. aeruginosa* to CF model cell cultures versus control cells showed that the production of exosomes is modified, with alterations appearing in their molecular composition dependent on each cell type. At the protein level, in both cell types, the levels of 30% of the species analyzed were altered, but differences were detected that affected both the specific species and their functions. At the level of miRNAs, alterations were detected in 18% of the species analyzed, showing, again, a pattern of differences dependent on the cell type, likely to affect the potential targets regulated by those species. The differences observed in this study suggest that the production of exosomes in response to the binding of *P. aeruginosa* in lung epithelial cells in CF is different from that in healthy cells, which could influence the response to infection.

## Data Availability

All the data presented in this study are contained within the article or Supplementary Materials.

## Supplementary Materials

The following supporting information can be downloaded at: https://www.mdpi.com/article/10.3390/ijms25020895/s1.
