# Supplementary material for: The Binding of Pseudomonas aeruginosa to Cystic Fibrosis Bronchial Epithelial Model Cells Alters the Composition of the Exosomes They Produce Compared to Healthy Control Cells
Source: Int J Mol Sci. 2024 Jan 11;25(2):895. doi: 10.3390/ijms25020895 (PMC10815301; doi:10.3390/ijms25020895)
Supplement: Supplementary file 1 [file ijms-25-00895-s001.zip › ijms-2784435-supplementary.pdf]

Supplementary Figure S1.

Prediction of the molecular functions of the genes potentially regulated by miRNAs whose relative abundance levels in exosomes were altered by *P. aeruginosa* adherence using the PANTHER classification system. (A) Genes regulated by miRNAs upregulated in NuLi and in CuFi; (B) Genes regulated by miRNAs upregulated in NuLi; (C) Genes regulated by miRNAs that are downregulated in NuLi; (D) Genes regulated by miRNA upregulated in CuFi; (C) Genes regulated by miRNAs that are downregulated in CuFi.

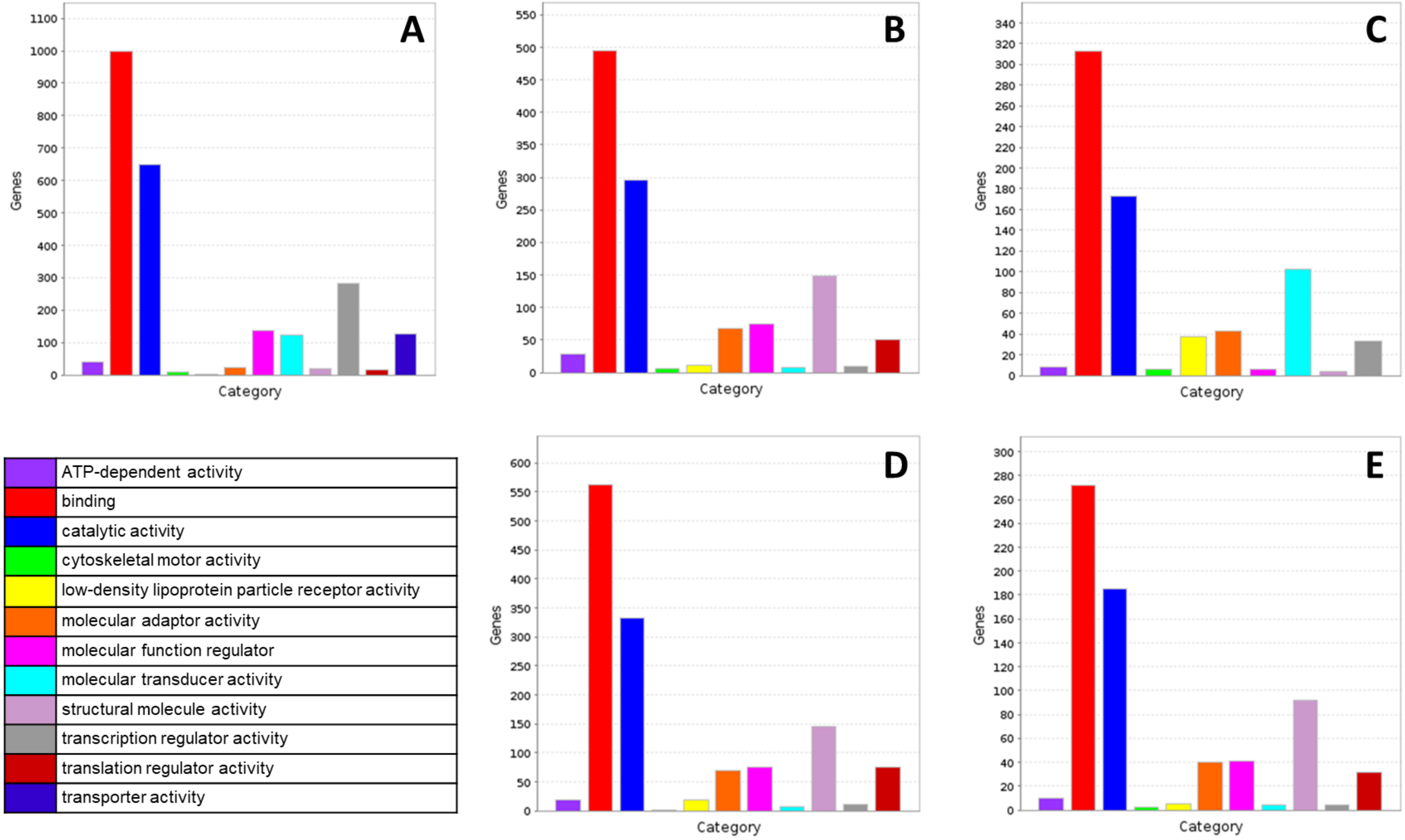

Supplementary Table S1. List of proteins identified by LC-MS/MS in exosomes isolated from NuLi and CuFi cell lines grown in isolation or in contact with *P. aeruginosa.*

Protein symbol	Uniprot Entry	NuLi	NuLi + *P. aeruginosa*	CuFi	CuFi + . *P. aeruginosa*
TF	P02787	✓	✓	✓	✓
LGALS3BP	Q08380	✓	✓	✓	✓
Immunoglobulin gamma-1 heavy chain	P0DOX5	✓	✓	✓	✓
IGHG1	P01857	✓	✓	✓	✓
HPX	P02790	✓	✓	✓	✓
LAMC1	P11047	✓	✓	✓	✓
PSMA7	O14818	✓	✓	✓	✓
ITIH4	Q14624	✓	✓		✓
AGRN	O00468	✓	✓	✓	✓
PARK7	Q99497	✓	✓	✓	✓
IGKC	P01834	✓	✓	✓	✓
PSMA6	P60900	✓	✓	✓	✓
HBB	P68871	✓	✓	✓	✓
ALB	P02769	✓	✓	✓	✓
HBG2	P69892	✓	✓	✓	✓
HBE1	P02100	✓	✓	✓	✓
HBG1	P69891	✓	✓	✓	✓
PRDX4	Q13162	✓	✓	✓	✓
THBS1	P07996	✓	✓	✓	✓
COL7A1	Q02388	✓	✓	✓	
PSMA5	P28066	✓	✓	✓	✓
GPI	P06744	✓	✓	✓	✓
FN1	P02751	✓	✓	✓	✓
DCD	P81605	✓	✓	✓	✓
LAMB1	P07942	✓	✓	✓	✓
KRT2	P35908	✓	✓	✓	✓
PSMB4	P28070	✓	✓	✓	✓
LAMA3	Q16787	✓	✓	✓	✓
ALB	P02768	✓	✓	✓	✓
DES	P17661	✓	✓	✓	✓
KRT77	Q7Z794	✓	✓	✓	✓
TAGLN	Q01995	✓	✓	✓	✓
PSMA3	P25788	✓	✓	✓	✓
CFL1	P23528	✓	✓	✓	✓
COL6A1	P12109	✓	✓	✓	✓
PRDX1	Q06830	✓	✓	✓	✓
KRT1	P04264	✓	✓	✓	✓
KRT78	Q8N1N4	✓	✓	✓	✓
CALM3	P0DP25	✓	✓	✓	✓

NEFH	P12036	✓	✓	✓	✓
HBA1	P69905	✓	✓	✓	✓
NID1	P14543	✓	✓		✓
APOE	P02649	✓	✓	✓	✓
PSMB3	P49720	✓	✓		✓
KRT5	P13647	✓	✓	✓	✓
PRDX2	P32119	✓	✓	✓	✓
DPYSL2	Q16555	✓	✓	✓	✓
KRT9	P35527	✓	✓	✓	✓
BSG	P35613	✓	✓	✓	✓
ACTN1	P12814	✓	✓	✓	✓
KRT15	P19012	✓	✓	✓	✓
VIM	P08670	✓	✓	✓	✓
KRT18	P05783	✓	✓	✓	✓
NME1	P15531	✓	✓	✓	✓
KRT31	Q15323	✓	✓	✓	✓
A2M	P01023	✓	✓	✓	✓
ADAM10	O14672	✓	✓		✓
PSMA1	P25786	✓	✓	✓	✓
PSMA4	P25789	✓	✓	✓	✓
SERPINB12	Q96P63	✓	✓	✓	✓
DSC1	Q08554	✓	✓	✓	✓
ENO3	P13929	✓	✓	✓	✓
TGM3	Q08188	✓	✓	✓	✓
APRT	P07741	✓	✓	✓	✓
CSTA	P01040	✓	✓	✓	✓
SDCBP	O00560	✓	✓	✓	✓
CD63	P08962	✓	✓	✓	✓
SERPINE1	P05121	✓	✓	✓	✓
ACTN4	O43707	✓	✓	✓	✓
KRT6B	P04259	✓	✓	✓	✓
FTH1	P02794	✓	✓	✓	✓
NME2	P22392	✓	✓	✓	✓
TGFBI	Q15582	✓	✓	✓	✓
LAMC2	Q13753	✓	✓	✓	✓
KRT10	P13645	✓	✓	✓	✓
LAMA5	O15230	✓	✓	✓	✓
HSPA2	P54652	✓	✓	✓	✓
IGSF1	Q8N6C5	✓	✓	✓	✓
PEBP1	P30086	✓	✓	✓	✓
RAB2A	P61019	✓	✓	✓	✓
KRT80	Q6KB66	✓	✓	✓	✓
DSG1	Q02413	✓	✓	✓	✓
ARHGDIA	P52565	✓	✓	✓	✓
PSMB1	P20618	✓	✓	✓	✓

CD9	P21926	✓	✓	✓	✓
KRT6A	P02538	✓	✓	✓	✓
NUCB1	Q02818	✓	✓	✓	✓
PKM	P14618	✓	✓	✓	✓
ALDOC	P09972	✓	✓	✓	✓
PRDX5	P30044	✓	✓	✓	✓
ENO2	P09104	✓	✓	✓	✓
TRAP1	Q12931	✓	✓	✓	✓
FH	P07954	✓	✓	✓	✓
PKP1	Q13835	✓	✓	✓	✓
HSPA8	P11142	✓	✓	✓	✓
LAMB3	Q13751	✓	✓		✓
KRT12	Q99456	✓	✓	✓	✓
ITGB1	P05556	✓	✓	✓	✓
HSPA1B	P0DMV9	✓	✓	✓	✓
YWHAE	P62258	✓	✓	✓	✓
UGP2	Q16851	✓	✓	✓	✓
CKB	P12277	✓	✓	✓	✓
PSMB5	P28074	✓	✓	✓	✓
KRT19	P08727	✓	✓	✓	✓
YWHAG	P61981	✓	✓	✓	✓
HLA-B	P01889	✓	✓	✓	✓
YWHAZ	P63104	✓	✓	✓	✓
PDCD6IP	Q8WUM4	✓	✓	✓	✓
PTGFRN	Q9P2B2	✓	✓	✓	✓
CSN1S2	P02663	✓	✓		
TPI1	P60174	✓	✓	✓	✓
YWHAH	Q04917	✓	✓	✓	✓
ANXA2	P07355	✓	✓	✓	✓
RAP1B	P61224	✓	✓	✓	✓
RAP1A	P62834	✓	✓	✓	✓
LCP1	P13796	✓	✓		✓
FAT1	Q14517	✓	✓		
EFNB1	P98172	✓	✓	✓	✓
KRT86	O43790	✓	✓		✓
KRT81	Q14533	✓	✓		✓
HSPA5	P11021	✓	✓	✓	✓
KRT14	P02533	✓	✓	✓	✓
RAC2	P15153	✓	✓	✓	✓
HNRNPK	P61978	✓	✓	✓	✓
ACTC1	P68032	✓	✓	✓	✓
ACTBL2	Q562R1	✓	✓	✓	✓
GNA13	Q14344	✓	✓	✓	✓
VCL	P18206	✓	✓	✓	✓
YWHAB	P31946	✓	✓	✓	✓

ITGA3	P26006	✓	✓	✓	✓
SFN	P31947	✓	✓	✓	✓
RAN	P62826	✓	✓	✓	✓
YWHAQ	P27348	✓	✓	✓	✓
KRT16	P08779	✓	✓	✓	✓
P4HB	P07237	✓	✓	✓	✓
CSN1S1	P02662	✓	✓	✓	✓
VAT1	Q99536	✓	✓	✓	✓
UCHL1	P09936	✓	✓	✓	✓
GDI2	P50395	✓	✓	✓	✓
NEFM	P07197	✓	✓	✓	✓
LDHA	P00338	✓	✓	✓	✓
RAB5C	P51148	✓	✓		✓
RAC1	P63000	✓	✓	✓	✓
SLC16A1	P53985	✓	✓	✓	✓
SELENBP1	Q13228	✓	✓	✓	
ARG1	P05089	✓	✓	✓	✓
CDSN	Q15517	✓	✓	✓	✓
DNAJC5	Q9H3Z4	✓	✓	✓	✓
CAT	P04040	✓	✓	✓	✓
CSN3	P02668	✓	✓	✓	
CALML5	Q9NZT1	✓	✓	✓	✓
CAPZB	P47756	✓	✓	✓	✓
SLC3A2	P08195	✓	✓	✓	✓
ACTG1	P63261	✓	✓	✓	✓
KRT17	Q04695	✓	✓	✓	✓
ACTB	P60709	✓	✓	✓	✓
ALDOA	P00883	✓	✓	✓	✓
ALDOA	P04075	✓	✓	✓	✓
ALDH9A1	P49189	✓	✓	✓	✓
RAB5A	P20339	✓	✓		✓
AHNAK	Q09666	✓	✓	✓	✓
LMAN1	P49257	✓	✓	✓	
HSP90AB1	P08238	✓	✓	✓	✓
EZR	P15311	✓	✓	✓	✓
KRT74	Q7RTS7	✓	✓	✓	✓
KRT73	Q86Y46	✓	✓	✓	✓
NEFL	P07196	✓	✓	✓	✓
TXNL1	O43396	✓	✓	✓	✓
LGALS1	P09382	✓	✓	✓	✓
CHMP4B	Q9H444	✓	✓	✓	✓
QSOX1	O00391	✓	✓		
PYGM	P11217	✓	✓	✓	✓
PDIA3	P30101	✓	✓	✓	✓
S100A14	Q9HCY8	✓	✓	✓	✓

HSPB1	P04792	✓	✓	✓	✓
CRMP1	Q14194	✓	✓	✓	✓
HSPA4	P34932	✓	✓	✓	✓
CTSD	P07339	✓	✓	✓	✓
HLA-C	P10321	✓	✓	✓	✓
LMNA	P02545	✓	✓	✓	✓
RPSA	P08865	✓	✓	✓	✓
CD81	P60033	✓	✓	✓	✓
CD82	P27701	✓	✓		
CD47	Q08722	✓	✓	✓	
POTEE	Q6S8J3	✓	✓	✓	✓
PGK1	P00558	✓	✓	✓	✓
FLNB	O75369	✓	✓	✓	✓
HSP90AA1	P07900	✓	✓	✓	✓
PFN1	P07737	✓	✓	✓	✓
RDX	P35241	✓	✓	✓	✓
SERPINB5	P36952	✓	✓	✓	✓
PTPRF	P10586	✓	✓		
ENO1	P06733	✓	✓	✓	✓
AHCY	P23526	✓	✓	✓	✓
GAPDH	P04406	✓	✓	✓	✓
KRT7	P08729	✓	✓	✓	✓
ALDH1A1	P00352	✓	✓	✓	✓
TUBA1B	P68363	✓	✓	✓	✓
TUBA1C	Q9BQE3	✓	✓	✓	✓
TUBB2B	Q9BVA1	✓	✓	✓	✓
RAB5B	P61020	✓	✓		✓
PGAM1	P18669	✓	✓	✓	
FAM149B1	Q96BN6	✓	✓	✓	
ANXA3	P12429	✓	✓	✓	✓
PSMC6	P62333	✓	✓	✓	
MYL6	P60660	✓	✓	✓	✓
TSG101	Q99816	✓	✓		
ATP1A1	P05023	✓	✓	✓	✓
ANXA5	P08758	✓	✓	✓	✓
TUBB2A	Q13885	✓	✓	✓	✓
HSP90B1	P14625	✓	✓	✓	✓
DSP	P15924	✓	✓	✓	✓
BASP1	P80723	✓	✓	✓	✓
SLC1A5	Q15758	✓	✓		✓
TUBB	P07437	✓	✓	✓	✓
ATP1A3	P13637	✓	✓	✓	✓
PPIA	P62937	✓	✓	✓	✓
DDB1	Q16531	✓	✓	✓	✓
VPS35	Q96QK1	✓	✓	✓	✓

TMEM163	Q8TC26	✓	✓		
TUBB4B	P68371	✓	✓	✓	✓
RPS3	P23396	✓	✓	✓	✓
EGFR	P00533	✓	✓		✓
KRT8	P05787	✓	✓	✓	✓
RAB35	Q15286	✓	✓	✓	✓
HLA-A	P04439	✓	✓	✓	✓
CS	O75390	✓	✓		✓
HSP90AB4P	Q58FF6	✓	✓	✓	✓
FLNA	P21333	✓	✓	✓	✓
EEF1A2	Q05639	✓	✓	✓	✓
RAB3A	P20336	✓	✓	✓	✓
PPIB	P23284	✓	✓	✓	✓
RAB15	P59190	✓	✓	✓	✓
GNAS	Q5JWF2	✓	✓	✓	✓
GNAS	P63092	✓	✓	✓	✓
INA	Q16352	✓	✓	✓	✓
UBC	P0CG48	✓	✓	✓	✓
UBB	P0CG47	✓	✓	✓	✓
UBA52	P62987	✓	✓	✓	✓
RPS27A	P62979	✓	✓	✓	✓
TUBB6	Q9BUF5	✓	✓	✓	✓
EEF1A1	P68104	✓	✓	✓	✓
EEF1A1P5	Q5VTE0	✓	✓	✓	✓
EEF1G	P26641	✓	✓	✓	✓
C3	P01024	✓	✓		✓
PRDX6	P30041	✓	✓	✓	
PGAM4	Q8N0Y7	✓	✓		
GDI1	P31150	✓	✓		
PGD	P52209	✓	✓	✓	✓
SEPTIN9	Q9UHD8	✓	✓	✓	✓
MFGE8	Q08431	✓	✓		✓
SEC22B	O75396	✓	✓		
TACSTD2	P09758	✓	✓	✓	
EIF2S3	P41091	✓	✓	✓	✓
TGM1	P22735	✓	✓	✓	✓
FGFBP1	Q14512	✓	✓	✓	
AMY2B	P19961	✓	✓	✓	
AMY2A	P04746	✓	✓	✓	
AMY1A	P0DUB6	✓	✓	✓	
PLD3	Q8IV08	✓	✓		
TSN	Q15631	✓	✓		
ANXA7	P20073	✓	✓		
TUBB3	Q13509	✓	✓	✓	✓
MSN	P26038	✓	✓	✓	✓

ATP1A2	P50993	✓	✓	✓	✓
EPHA2	P29317	✓	✓	✓	✓
GSTP1	P09211	✓	✓	✓	✓
RAB1B	Q9H0U4	✓	✓	✓	✓
ITGB4	P16144	✓	✓	✓	✓
EIF4A2	Q14240	✓	✓	✓	✓
ARRDC1	Q8N5I2	✓	✓		✓
HSPG2	P98160	✓	✓	✓	✓
PYGL	P06737	✓	✓	✓	✓
RAB11B	Q15907	✓	✓	✓	✓
TUBB4A	P04350	✓	✓	✓	✓
PGM1	P36871	✓	✓	✓	✓
ANXA6	P08133	✓	✓	✓	✓
ANPEP	P15144	✓	✓	✓	✓
RAB1A	P62820	✓	✓	✓	✓
WDR1	O75083	✓	✓	✓	✓
TARS1	P26639	✓	✓	✓	✓
VAMP3	Q15836	✓	✓	✓	✓
ALDH1A2	O94788	✓	✓		✓
PPP1CB	P62140	✓	✓	✓	✓
ITGB6	P18564	✓	✓	✓	
GMPPB	Q9Y5P6	✓	✓	✓	✓
TMED9	Q9BVK6	✓	✓	✓	✓
SF3B3	Q15393	✓	✓	✓	✓
UBE2V1	Q13404	✓	✓	✓	✓
OTUB1	Q96FW1	✓	✓		✓
ERP29	P30040	✓	✓		
HNRNPA2B1	P22626	✓	✓		
CTNNA1	P35221	✓	✓	✓	✓
SLC7A5	Q01650	✓	✓	✓	✓
ANXA1	P04083	✓	✓	✓	✓
PYGB	P11216	✓	✓	✓	✓
EEF2	P13639	✓	✓	✓	✓
ITGA2	P17301	✓	✓	✓	✓
RAB3B	P20337	✓	✓	✓	✓
COL12A1	Q99715	✓	✓	✓	✓
RAB3D	O95716	✓	✓	✓	✓
ITGA6	P23229	✓	✓	✓	✓
RAB3C	Q96E17	✓	✓	✓	✓
GNB2	P62879	✓	✓	✓	✓
GNB1	P62873	✓	✓	✓	✓
VAMP2	P63027	✓	✓	✓	✓
EHD3	Q9NZN3	✓	✓	✓	✓
GNG12	Q9UBI6	✓	✓	✓	✓
PDCD6	O75340	✓	✓	✓	✓

S100A9	P06702	✓	✓	✓	✓
USP14	P54578	✓	✓	✓	
IGSF8	Q969P0	✓	✓	✓	✓
PPP3CA	Q08209	✓	✓	✓	✓
TUBA4A	P68366	✓	✓	✓	✓
EIF4A1	P60842	✓	✓	✓	✓
DPYSL3	Q14195	✓	✓	✓	✓
HYOU1	Q9Y4L1	✓	✓	✓	✓
EHD2	Q9NZN4	✓	✓	✓	✓
UBA1	P22314	✓	✓	✓	✓
RAB7A	P51149	✓	✓		
ACO1	P21399	✓	✓	✓	✓
PGM2	Q96G03	✓	✓		✓
CLIC1	O00299	✓	✓	✓	✓
GSN	P06396	✓	✓	✓	✓
STAT1	P42224	✓	✓	✓	
MAP1B	P46821	✓	✓		
MPZL1	O95297	✓	✓	✓	✓
LSR	Q86X29	✓	✓		
ATP5F1B	P06576	✓	✓	✓	✓
LDHB	P07195	✓	✓	✓	✓
PAFAH1B1	P43034	✓	✓	✓	✓
EHD1	Q9H4M9	✓	✓	✓	✓
NPEPPS	P55786	✓	✓	✓	✓
ANXA4	P09525	✓	✓	✓	
PSMC2	P35998	✓	✓	✓	✓
FSCN1	Q16658	✓	✓	✓	✓
CASP14	P31944	✓	✓		✓
RPL11	P62913	✓	✓	✓	✓
AK1	P00568	✓	✓		
RPL18	Q07020	✓	✓		
B4GAT1	O43505	✓	✓		
PLS3	P13797	✓	✓		
VCP	P55072	✓	✓	✓	✓
STOM	P27105	✓	✓	✓	✓
PLXNB2	O15031	✓	✓	✓	✓
GNAI3	P08754	✓	✓	✓	✓
JUP	P14923	✓	✓	✓	✓
LRP1	Q07954	✓	✓		
SEPTIN2	Q15019	✓	✓	✓	✓
CPNE3	O75131	✓	✓	✓	✓
ATP1B1	P05026	✓	✓	✓	
IST1	P53990	✓	✓	✓	✓
AKR1A1	P14550	✓	✓		✓
XPO1	O14980	✓	✓		✓

TCP1	P17987	✓	✓	✓	✓
ALCAM	Q13740	✓	✓		
ATP6V0A1	Q93050	✓	✓		
RPS16	P62249	✓	✓	✓	✓
PSMC3	P17980	✓	✓		
CTNNB1	P35222	✓	✓	✓	✓
SPTAN1	Q13813	✓	✓	✓	✓
ATP5F1A	P25705	✓	✓	✓	✓
GNAO1	P09471	✓	✓		✓
SPTBN1	Q01082	✓	✓	✓	✓
PSMD11	O00231	✓	✓		
SLC16A3	O15427	✓	✓	✓	
MARCKS	P29966	✓	✓	✓	✓
SEPTIN11	Q9NVA2	✓	✓	✓	✓
ATIC	P31939	✓	✓		✓
FMNL2	Q96PY5	✓	✓		
LRBA	P50851	✓	✓	✓	✓
CANX	P27824	✓	✓		
ATP2B1	P20020	✓	✓	✓	✓
ITGA5	P08648	✓	✓		✓
RTCB	Q9Y3I0	✓	✓	✓	✓
ALDH2	P05091	✓	✓		
CAND1	Q86VP6	✓	✓		
ATP2B4	P23634	✓	✓	✓	✓
ARF5	P84085	✓	✓	✓	
EHD4	Q9H223	✓	✓	✓	✓
KIF5B	P33176	✓	✓		
ITGAV	P06756	✓	✓	✓	
NT5E	P21589	✓	✓	✓	✓
PLXNA2	O75051	✓	✓	✓	✓
PFKP	Q01813	✓	✓	✓	
RAB14	P61106	✓	✓	✓	✓
CLTC	Q00610	✓	✓	✓	✓
GNAI2	P04899	✓	✓	✓	✓
ARF3	P61204	✓	✓	✓	
ARF1	P84077	✓	✓	✓	
H4C1	P62805	✓	✓	✓	✓
NSF	P46459	✓	✓		
COPA	P53621	✓	✓	✓	✓
KRT6C	P48668	✓		✓	✓
ACTA1	P68133	✓		✓	✓
ACTA2	P62736	✓		✓	✓
ACTG2	P63267	✓		✓	✓
KRT15	O77727	✓		✓	✓
HBD	P02042	✓		✓	✓

KRT79	Q5XKE5	✓		✓	✓
KRT13	P13646	✓		✓	✓
KRT75	O95678	✓		✓	✓
KRT3	P12035	✓		✓	✓
THBS2	P35442	✓		✓	✓
KRT24	Q2M2I5	✓		✓	✓
KRT28	Q7Z3Y7	✓		✓	✓
POTEF	A5A3E0	✓		✓	✓
VCAN	P13611	✓		✓	✓
ANXA2P2	A6NMY6	✓		✓	✓
POTEKP	Q9BYX7	✓		✓	✓
TUBA1A	Q71U36	✓		✓	✓
KRT76	Q01546	✓		✓	✓
APOD	P05090	✓		✓	✓
PSMA8	Q8TAA3	✓		✓	✓
CFI	P05156	✓			✓
TUBA3E	Q6PEY2	✓		✓	✓
FAT2	Q9NYQ8	✓			
TUBA3C	P0DPH7	✓		✓	✓
LAMA1	P25391	✓			
IGHA1	P01876	✓		✓	✓
HSPA1L	P34931	✓		✓	✓
EFEMP1	Q12805	✓		✓	
KRT27	Q7Z3Y8	✓		✓	✓
KRT25	Q7Z3Z0	✓		✓	✓
KRT33B	Q14525	✓		✓	✓
IGHG3	P01860	✓		✓	✓
lacZ	P00722	✓		✓	✓
LYZ	P00698	✓		✓	✓
CST3	P01034	✓		✓	✓
IGHG2	P01859	✓		✓	✓
HSPA6	P17066	✓		✓	✓
TIMP1	P01033	✓		✓	✓
HSPA7	P48741	✓		✓	✓
TUBB8	Q3ZCM7	✓		✓	✓
HSP90AB3P	Q58FF7	✓		✓	✓
LTF	P02788	✓		✓	✓
TUBB8B	A6NNZ2	✓		✓	✓
KRT35	Q92764	✓		✓	✓
KRT36	O76013	✓		✓	✓
KRT37	O76014	✓		✓	✓
KRT32	Q14532	✓		✓	✓
KRT38	O76015	✓		✓	✓
TPM2	P07951	✓			✓
POTEI	P0CG38	✓		✓	✓

TUBB1	Q9H4B7	✓		✓	✓
HSP90AA2P	Q14568	✓		✓	✓
TPM4	P67936	✓			✓
LCN1	P31025	✓		✓	✓
CFB	P00751	✓			✓
CHGA	P10645	✓		✓	✓
KRT71	Q3SY84	✓		✓	✓
THBS3	P49746	✓			
KRT72	Q14CN4	✓		✓	✓
AZGP1	P25311	✓		✓	✓
CFL2	Q9Y281	✓		✓	✓
ACTN3	Q08043	✓		✓	✓
TUBA8	Q9NY65	✓		✓	✓
KRT84	Q9NSB2	✓		✓	✓
HSP90AB2P	Q58FF8	✓		✓	✓
POTEJ	P0CG39	✓		✓	✓
RAB1C	Q92928	✓		✓	✓
IGHG4	P01861	✓			✓
PSMB6	P28072	✓		✓	✓
GNA12	Q03113	✓		✓	✓
KPRP	Q5T749	✓		✓	✓
Immunoglobulin alpha-2 heavy chain	P0DOX2	✓		✓	✓
PIP	P12273	✓		✓	
IGHA2	P01877	✓		✓	✓
GAPDHS	O14556	✓		✓	✓
RAB2B	Q8WUD1	✓		✓	✓
LYZ	P61626	✓		✓	✓
PKLR	P30613	✓		✓	✓
KRT4	P19013	✓		✓	✓
SPARC	P09486	✓		✓	✓
PTX3	P26022	✓			✓
DKK3	Q9UBP4	✓			
NME2P1	O60361	✓		✓	
CLSTN1	O94985	✓		✓	✓
IGFBP7	Q16270	✓		✓	✓
PZP	P20742	✓			✓
GLOD4	Q9HC38	✓		✓	✓
PSMB2	P49721	✓		✓	✓
TPM1	P09493	✓			✓
RAC3	P60763	✓		✓	✓
TPM3	P06753	✓			✓
TUBA4B	Q9H853	✓		✓	✓
SET	Q01105	✓		✓	✓
CLU	P10909	✓			

S100A7	P31151	✓		✓	✓
S100A8	P05109	✓		✓	✓
TXN	P10599	✓		✓	✓
LACRT	Q9GZZ8	✓		✓	✓
XP32	Q5T750	✓		✓	✓
HLA-H	P01893	✓		✓	✓
ST13	P50502	✓		✓	✓
SDC1	P18827	✓		✓	✓
ST13P5	Q8NFI4	✓		✓	✓
SKP1	P63208	✓		✓	✓
EEF1B2	P24534	✓		✓	✓
RRBP1	Q9P2E9	✓		✓	✓
NPEPPSL1	A6NEC2	✓		✓	✓
APP	P05067	✓			
PTMA	P06454	✓		✓	✓
RAB11A	P62491	✓		✓	✓
SERPINI1	Q99574	✓			✓
MDH1	P40925	✓		✓	✓
S100A2	P29034	✓		✓	✓
ST13P4	Q8IZP2	✓		✓	✓
PSMA2	P25787	✓		✓	✓
TMSB4X	P62328	✓			✓
HSP90AA5P	Q58FG0	✓		✓	✓
Ras-related protein Rap-1b-like protein	A6NIZ1	✓		✓	✓
DSTN	P60981	✓		✓	✓
GLUD1	P00366	✓		✓	✓
GLUD2	P49448	✓		✓	✓
GLUD1	P00367	✓		✓	✓
PRSS23	O95084	✓		✓	
GNB4	Q9HAV0	✓		✓	✓
LCN1P1	Q5VSP4	✓		✓	✓
GPX3	P22352	✓			
KRT87P	A6NCN2	✓			✓
KRT85	P78386	✓			✓
KRT83	P78385	✓			✓
CA2	P00921	✓			
M6PR	P20645	✓		✓	✓
CHST6	Q9GZX3	✓			
XPNPEP3	Q9NQH7	✓		✓	✓
HSP90B2P	Q58FF3	✓		✓	✓
TADA2B	Q86TJ2	✓		✓	✓
FBN1	P35555	✓			
CTNNA2	P26232	✓		✓	✓
DNPEP	Q9ULA0	✓			✓

TMBIM1	Q969X1	✓		✓	✓
CSPG4	Q6UVK1	✓			
PCSK9	Q8NBP7	✓			✓
ATP1A4	Q13733	✓		✓	✓
TIMP2	P16035	✓		✓	✓
SDCBP2	Q9H190	✓		✓	✓
ACTN2	P35609	✓		✓	✓
ERO1A	Q96HE7	✓		✓	✓
PLOD2	O00469	✓		✓	✓
SDC4	P31431	✓		✓	✓
PGAM2	P15259	✓			
STIP1	P31948	✓		✓	
MYL6B	P14649	✓		✓	✓
GPC1	P35052	✓			
KRT33A	O76009	✓			✓
RPLP2	P05387	✓		✓	✓
PGK2	P07205	✓		✓	✓
STMN1	P16949	✓			✓
CAPZA1	P52907	✓		✓	✓
ALOX12B	O75342	✓		✓	✓
PRPH	P41219	✓		✓	✓
FABP5	Q01469	✓		✓	✓
HDGFL3	Q9Y3E1	✓		✓	✓
LDLR	P01130	✓			✓
ITIH2	P19823	✓			✓
NPC2	P61916	✓			
CPB2	Q96IY4	✓			✓
CAPZA2	P47755	✓		✓	✓
FSTL1	Q12841	✓			
PTMS	P20962	✓			
PGLYRP2	Q96PD5	✓			
HSP90AA4P	Q58FG1	✓			
MAPT	P10636	✓			
SCG2	P13521	✓		✓	✓
AXL	P30530	✓			
SPOCK3	Q9BQ16	✓			
SPOCK1	Q08629	✓			
SLC7A7	Q9UM01	✓		✓	✓
SLC7A6	Q92536	✓		✓	✓
FICD	Q9BVA6	✓			
MANF	P55145	✓			
DSG2	Q14126	✓			
CCN1	O00622	✓			
SCAMP2	O15127	✓		✓	✓
HSPE1	P61604	✓		✓	✓

SYPL1	Q16563	✓		✓	✓
APOA1	P02647	✓			
HLA-G	P17693	✓		✓	✓
CDH3	P22223	✓		✓	✓
CCBE1	Q6UXH8	✓			
DBN1	Q16643	✓		✓	
ATP2B3	Q16720	✓		✓	✓
Immunoglobulin kappa light chain	P0DOX7	✓		✓	✓
EPCAM	P16422	✓			
CSRP1	P21291	✓			
PTPRK	Q15262	✓			
SLC38A2	Q96QD8	✓		✓	✓
KTN1	Q86UP2	✓		✓	✓
EEF1D	P29692	✓		✓	✓
HLA-E	P13747	✓		✓	✓
MTPN	P58546	✓		✓	✓
MCAM	P43121	✓		✓	✓
STX12	Q86Y82	✓			
EIF5AL1	Q6IS14	✓		✓	✓
EIF5A	P63241	✓		✓	✓
AP3D1	O14617	✓		✓	✓
CAV1	Q03135	✓		✓	✓
SH3BGRL	O75368	✓			
LAMP2	P13473	✓		✓	
CASK	O14936	✓		✓	✓
NAP1L1	P55209	✓		✓	✓
NAP1L4	Q99733	✓		✓	✓
MATN3	O15232	✓			
SLC44A1	Q8WWI5	✓			
PHGDH	O43175	✓		✓	
DSC3	Q14574	✓		✓	✓
MSLN	Q13421	✓			
LUM	P51884	✓			✓
PPIE	Q9UNP9	✓			
PEPD	P12955	✓			
GSDMA	Q96QA5	✓			
KRT20	P35900	✓		✓	✓
GGCT	O75223	✓			✓
DIRAS2	Q96HU8	✓		✓	✓
TFG	Q92734	✓		✓	✓
KIFBP	Q96EK5	✓			
SNX2	O60749	✓		✓	✓
SNX1	Q13596	✓		✓	✓
GSTM1	P09488	✓			

DBI	P07108	✓			
PPP3CB	P16298	✓		✓	✓
HNRNPD	Q14103	✓		✓	✓
POLE	Q07864	✓			
JAG1	P78504	✓			
SETSIP	P0DME0	✓		✓	✓
MAPRE1	Q15691	✓		✓	✓
CP	P00450	✓			
HEXB	P07686	✓		✓	
NBEA	Q8NFP9	✓		✓	✓
STMN2	Q93045	✓			✓
MAP4	P27816	✓			
UBE2V2	Q15819	✓		✓	✓
NIT2	Q9NQR4	✓		✓	✓
DMBT1	Q9UGM3	✓			
ADRM1	Q16186	✓		✓	✓
SCGB2A1	O75556	✓			✓
ERVMER34-1	Q9H9K5	✓			
GUCY1A2	P33402	✓			
VPS28	Q9UK41	✓			
GOLM1	Q8NBJ4	✓			
GPM6A	P51674	✓			
NFU1	Q9UMS0	✓			✓
COL14A1	Q05707	✓			
ING5	Q8WYH8	✓			✓
ING4	Q9UNL4	✓			✓
SERPINB14	P01012	✓			
PSAP	P07602	✓			
CADM1	Q9BY67	✓			
EVA1A	Q9H8M9	✓			
TINAGL1	Q9GZM7	✓			
GIT1	Q9Y2X7	✓			
GIT2	Q14161	✓			
KAT6B	Q8WYB5	✓			
SEC23A	Q15436	✓			
PCBD1	P61457	✓			✓
NRP1	O14786	✓			
KCTD12	Q96CX2	✓			
DYNC1I2	Q13409	✓		✓	✓
LZTFL1	Q9NQ48	✓			
TSPAN14	Q8NG11	✓			
QPCT	Q16769	✓			
MMP9	P14780	✓			
SERPINB4	P48594	✓			
RAG1	P15918	✓			✓

EML4	Q9HC35	✓		✓	✓
IGLC3	P0DOY3	✓			
IGLC2	P0DOY2	✓			
IGLC1	P0CG04	✓			
Immunoglobulin lambda-1 light chain	P0DOX8	✓			
IGLC6	P0CF74	✓			
IGLL5	B9A064	✓			
IGLC7	A0M8Q6	✓			
TSPAN9	O75954	✓			
EPHB2	P29323	✓			
FBN2	P35556	✓			
DYNC1H1	Q14204		✓	✓	✓
PLEC	Q15149		✓	✓	✓
RAB8A	P61006		✓		
FASN	P49327		✓	✓	✓
RAB10	P61026		✓		
RAB8B	Q92930		✓		
ATP6V1A	P38606		✓		
TLN1	Q9Y490		✓	✓	
CUL4B	Q13620		✓		
PSMD3	O43242		✓		✓
COL1A1	P02452		✓		
RAB6A	P20340		✓		
MYH9	P35579		✓	✓	✓
DIP2B	Q9P265		✓		
ARF4	P18085		✓	✓	
COL1A2	P08123		✓		
COPB1	P53618		✓		✓
PSMD2	Q13200		✓		
RAB6B	Q9NRW1		✓		
GFPT1	Q06210		✓		✓
AP2B1	P63010		✓		
RACK1	P63244		✓	✓	✓
TF	Q29443		✓		
COL6A3	P12111		✓		
SLC25A5	P05141		✓		
TKT	P29401		✓	✓	✓
GNAI1	P63096		✓		
HSPD1	P10809		✓		✓
CKAP4	Q07065		✓		
CADPS	Q9ULU8		✓		
CSE1L	P55060		✓		
GCN1	Q92616		✓		
PFKL	P17858		✓		

CCT3	P49368		✓		
EIF4A3	P38919		✓		
SLC25A6	P12236		✓		
S100A16	Q96FQ6		✓		
SEC31A	O94979		✓		
ATP2A2	P16615		✓		
AP1B1	Q10567		✓		
CDCP1	Q9H5V8		✓		
RAB13	P51153		✓		
IQGAP1	P46940		✓	✓	✓
CCT4	P50991		✓	✓	✓
ATP6V1B2	P21281		✓	✓	✓
CTNND1	O60716		✓		
ATP8A1	Q9Y2Q0		✓		
RPN1	P04843		✓		
EPRS1	P07814		✓		
GANAB	Q14697		✓		
ANXA11	P50995		✓		
RPS9	P46781		✓		
AP2M1	Q96CW1		✓		
MYO1B	O43795		✓		✓
CCT8	P50990		✓	✓	✓
PSMC5	P62195		✓		
COPB2	P35606		✓		
SYT1	P21579		✓		
RRAS	P10301		✓		
ARPC1A	Q92747		✓		
ARL8B	Q9NVJ2		✓		
RPS4X	P62701		✓		
CCT6A	P40227		✓	✓	✓
HRNR	Q86YZ3		✓		
ACLY	P53396		✓		
GNAQ	P50148		✓		
SLC25A4	P12235		✓		
MVP	Q14764		✓	✓	
SRC	P12931		✓	✓	
STXBP1	P61764		✓		
MYOF	Q9NZM1		✓		
NID2	Q14112		✓		
DNM2	P50570		✓		
PFKM	P08237		✓		
UBR4	Q5T4S7		✓		
AP2A1	O95782		✓		
RPS2	P15880		✓		
CCT7	Q99832		✓		

RPL4	P36578		✓		
MYO1C	O00159		✓		
RALB	P11234		✓		
MAPK1	P28482		✓		
VAT1L	Q9HCJ6		✓		
ACTR1A	P61163		✓		
PPP2R1A	P30153		✓		
H3-3A	P84243		✓		
H3-4	Q16695		✓		
H3C1	P68431		✓		
H3C15	Q71DI3		✓		
HSPA9	P38646		✓	✓	✓
COL17A1	Q9UMD9		✓		✓
TAGLN2	P37802		✓	✓	✓
SYT5	O00445		✓		✓
FERMT1	Q9BQL6		✓		
PLXNA1	Q9UIW2		✓		
ARPC2	O15144		✓		
PSMD1	Q99460		✓		
PI4K2A	Q9BTU6		✓		
PSMC4	P43686		✓		
GNA11	P29992		✓		
DDX39B	Q13838		✓		
SEPTIN6	Q14141		✓		
AHCYL1	O43865		✓		
RAB33B	Q9H082		✓		
CAD	P27708		✓	✓	
PSMC1	P62191		✓	✓	✓
GARS1	P41250		✓		✓
SND1	Q7KZF4		✓		✓
AARS1	P49588		✓	✓	
BAIAP2	Q9UQB8		✓		
KPNB1	Q14974		✓		
VDAC1	P21796		✓		
SLC12A2	P55011		✓		
USO1	O60763		✓		
CYFIP1	Q7L576		✓		
RPS18	P62269		✓		
EIF3L	Q9Y262		✓		
HNRNPM	P52272		✓		
ATP9A	O75110		✓		
STT3A	P46977		✓		
COPG1	Q9Y678		✓		
ACOT7	O00154		✓		
XPO7	Q9UIA9		✓		

PSMD13	Q9UNM6		✓		
PCBP1	Q15365		✓		
RALA	P11233		✓		
TMED10	P49755		✓		
AP1M1	Q9BXS5		✓		
SEPTIN7	Q16181		✓		
SEPTIN8	Q92599		✓		
EIF3A	Q14152		✓		
IARS1	P41252		✓		
ARL8A	Q96BM9		✓		
RAB4B	P61018		✓		
ACTR2	P61160		✓		✓
H2BC15	Q99877		✓	✓	✓
H2BC5	P58876		✓	✓	✓
H2BC12	O60814		✓	✓	✓
H2BC14	Q99879		✓	✓	✓
H2BS1	P57053		✓	✓	✓
H2BC18	Q5QNW6		✓	✓	✓
H2BC4	P62807		✓	✓	✓
H2BC9	Q93079		✓	✓	✓
H2BC13	Q99880		✓	✓	✓
PDIA4	P13667		✓		✓
F11R	Q9Y624		✓	✓	
ABCE1	P61221		✓	✓	✓
PSMD6	Q15008		✓		
SNAP25	P60880		✓		
FLOT2	Q14254		✓		
HUWE1	Q7Z6Z7		✓		
GLG1	Q92896		✓		
MTHFD1	P11586		✓		
ERLIN2	O94905		✓		
ACTR3	P61158		✓		
DNM1	Q05193		✓		
RPL7	P18124		✓		
G6PD	P11413		✓		
DARS1	P14868		✓		
TUFM	P49411		✓		
SERPINH1	P50454		✓		
FARSB	Q9NSD9		✓		
AGL	P35573		✓		
RAB27A	P51159		✓		
YES1	P07947		✓		
RAP2B	P61225		✓		
CD109	Q6YHK3		✓		
LTA4H	P09960		✓		

RRAS2	P62070		✓		
SAR1B	Q9Y6B6		✓		
GOT2	P00505		✓		
AP1M2	Q9Y6Q5		✓		
YKT6	O15498		✓		
ERP44	Q9BS26		✓		
CCT5	P48643		✓		
RPL13	P26373		✓		
SRI	P30626		✓		
HK1	P19367		✓		
SEC61A1	P61619		✓		
ACTR1B	P42025		✓		
ELAPOR1	Q6UXG2		✓		
SERPINB3	P29508		✓		
SLC2A1	P11166		✓		
CPE	P16870		✓		
SLC38A5	Q8WUX1		✓		
RAB43	Q86YS6		✓		
RAB12	Q6IQ22		✓		
RAB4A	P20338		✓		
AVP	P01185		✓		
MAT2A	P31153		✓	✓	✓
ACO2	Q99798		✓		✓
H2BC21	Q16778		✓	✓	✓
H2BC17	P23527		✓	✓	✓
H2BC3	P33778		✓	✓	✓
H2BC11	P06899		✓	✓	✓
H2BU1	Q8N257		✓	✓	✓
TMEM43	Q9BTV4		✓	✓	
COL4A2	P08572		✓	✓	✓
MMP14	P50281		✓		✓
CD44	P16070		✓	✓	
HNRNPU	Q00839		✓		✓
PA2G4	Q9UQ80		✓	✓	
IDH2	P48735		✓		
PHB	P35232		✓		
DDX17	Q92841		✓		
TFRC	P02786		✓		
DHX9	Q08211		✓		
PHB2	Q99623		✓		
CACNA2D1	P54289		✓		
ALDH18A1	P54886		✓		
MAP4K4	O95819		✓		
MLEC	Q14165		✓		
CNP	P09543		✓		

BUB3	O43684		✓		
DDOST	P39656		✓		
RPN2	P04844		✓		
AP1G1	O43747		✓		
AP2A2	O94973		✓		
SAR1A	Q9NR31		✓		
ERGIC1	Q969X5		✓		
VPS29	Q9UBQ0		✓		
COL6A2	P12110		✓		
KLC1	Q07866		✓		
RPL6	Q02878		✓		
LANCL1	O43813		✓		
PGRMC1	O00264		✓		
RPL15	P61313		✓		
KLC2	Q9H0B6		✓		
TM9SF4	Q92544		✓		
GNB3	P16520		✓		
SLC25A31	Q9H0C2		✓		
RTN1	Q16799		✓		
ESD	P10768		✓		
ATP1B3	P54709		✓		✓
QARS1	P47897		✓	✓	✓
HRAS	P01112		✓		✓
NRAS	P01111		✓		✓
RUVBL2	Q9Y230		✓	✓	
PRMT1	Q99873		✓		
RPS8	P62241		✓		
VARS1	P26640		✓		
ITGA1	P56199		✓		
NCAM1	P13591		✓		
SERPINE2	P07093		✓		
CPNE1	Q99829		✓		
TMED4	Q7Z7H5		✓		
RARS1	P54136		✓		
KIF5C	O60282		✓		
COPS4	Q9BT78		✓		
HSD17B4	P51659		✓		
ETFB	P38117		✓		
HPRT1	P00492		✓		
PXDN	Q92626		✓		
UPF1	Q92900		✓		
ATP6AP2	O75787		✓		
RAB18	Q9NP72		✓		
DDX1	Q92499		✓		
MINK1	Q8N4C8		✓		

SRP68	Q9UHB9		✓		
UBE2N	P61088		✓		
CYB5R3	P00387		✓		
CPD	O75976		✓		
TM9SF2	Q99805		✓		
SF3B1	O75533		✓		
ARCN1	P48444		✓		
TTYH3	Q9C0H2		✓		
ATP6V0D1	P61421		✓		
FLG	P20930		✓		
SART3	Q15020		✓		
ECPAS	Q5VYK3		✓		
AP3B1	O00203		✓		
SNRNP200	O75643		✓		
RPS3A	P61247		✓		
RPS6KA3	P51812		✓		
RAB21	Q9UL25		✓		
CSNK2A1	P68400		✓		
CSNK2A3	Q8NEV1		✓		
SRP54	P61011		✓		
CYFIP2	Q96F07		✓		
RPS15A	P62244		✓		
VPS13C	Q709C8		✓		
RPL5	P46777		✓		
USP5	P45974		✓		
RPS11	P62280		✓		
PRKACA	P17612		✓		
CAB39	Q9Y376		✓		
AP3B2	Q13367		✓		
RPL10A	P62906		✓		
SHMT2	P34897		✓		
OXCT1	P55809		✓		
PTPRN	Q16849		✓		
PTPN9	P43378		✓		
SYP	P08247		✓		
STX4	Q12846		✓		
GNA14	O95837		✓		
RPS14	P62263		✓		
SCARB1	Q8WTV0		✓		
RPL3	P39023		✓		
SLC29A1	Q99808		✓		
ARL1	P40616		✓		
MAP2K2	P36507		✓		
S100A11	P31949		✓		
ADH5	P11766		✓		

HACD3	Q9P035		✓		
RPL13AP3	Q6NVV1		✓		
RPL13A	P40429		✓		
GNG5	P63218		✓		
HM13	Q8TCT9		✓		
RPL23	P62829		✓		
RTN3	O95197		✓		
RHOC	P08134		✓		
RHOA	P61586		✓		
CCT2	P78371		✓	✓	✓
RPLP0	P05388		✓	✓	✓
IDH1	O75874		✓	✓	✓
RPL7A	P62424		✓		✓
ARPC4	P59998		✓	✓	✓
H2AZ1	P0C0S5		✓		✓
H2AZ2	Q71UI9		✓		✓
RPS25	P62851		✓	✓	✓
EIF3E	P60228		✓	✓	✓
IDH3A	P50213		✓		
MDH2	P40926		✓		
APMAP	Q9HDC9		✓		
GIPC1	O14908		✓		
HSPH1	Q92598		✓		
EPB41L2	O43491		✓		
PSMD12	O00232		✓		
ARF6	P62330		✓		
GMPS	P49915		✓		
SARS1	P49591		✓		
VDAC2	P45880		✓		
HADHB	P55084		✓		
PSME1	Q06323		✓		
ERLIN1	O75477		✓		
FLG2	Q5D862		✓		
CYRIB	Q9NUQ9		✓		
WASHC5	Q12768		✓		
CTBP1	Q13363		✓		
DDX5	P17844		✓		
PTPN23	Q9H3S7		✓		
PTK7	Q13308		✓		
MARCKSL1	P49006		✓		
IDH3B	O43837		✓		
STRAP	Q9Y3F4		✓		
CAMK2B	Q13554		✓		
CAMK2D	Q13557		✓		
CNN1	P51911		✓		

MYH10	P35580		✓		
SACM1L	Q9NTJ5		✓		
CDH1	P12830		✓		
LAP3	P28838		✓		
CMAS	Q8NFW8		✓		
HSPA13	P48723		✓		
KIF1A	Q12756		✓		
DLG1	Q12959		✓		
PSMD5	Q16401		✓		
HNRNPA1	P09651		✓		
CAPN1	P07384		✓		
STXBP3	O00186		✓		
DCTN1	Q14203		✓		
VAMP7	P51809		✓		
MYO1D	O94832		✓		
DRG2	P55039		✓		
TTLL12	Q14166		✓		
PCBP2	Q15366		✓		
CUL3	Q13618		✓		
GBE1	Q04446		✓		
PSMD8	P48556		✓		
IPO5	O00410		✓		
RHEB	Q15382		✓		
GALE	Q14376		✓		
SPCS2	Q15005		✓		
RPL26	P61254		✓		
RPL26L1	Q9UNX3		✓		
ARPC3	O15145		✓		
CDC42	P60953		✓		
RHOG	P84095		✓		
POMC	P01189		✓		
TM9SF3	Q9HD45		✓		
EPHB4	P54760		✓		
VPS37B	Q9H9H4		✓		
PSMD14	O00487		✓		
SEPTIN5	Q99719		✓		
AKR1B1	P15121		✓		
PRPS1	P60891		✓		
PNP	P00491		✓		
CRYM	Q14894		✓		
S100A6	P06703		✓		
VAMP1	P23763		✓		
ALDH1L1	O75891		✓		
ALDH1L2	Q3SY69		✓		
MYL12B	O14950		✓	✓	

MYL12A	P19105		✓	✓	
CAP1	Q01518		✓	✓	✓
LAMP1	P11279		✓	✓	✓
RBBP4	Q09028		✓		✓
PRPF19	Q9UMS4		✓		✓
S100A10	P60903		✓	✓	
SLC6A8	P48029		✓	✓	✓
FDPS	P14324		✓	✓	✓
COPS2	P61201		✓	✓	✓
FERMT2	Q96AC1		✓		
PBXIP1	Q96AQ6		✓		
DYNC1LI1	Q9Y6G9		✓		
STX1B	P61266		✓		
SYNGR1	O43759		✓		
RPL24	P83731		✓		
ASNS	P08243		✓		
PACSIN2	Q9UNF0		✓		
NAPA	P54920		✓		
LLGL1	Q15334		✓		
OGDH	Q02218		✓		
CTBP2	P56545		✓		
NCKAP1	Q9Y2A7		✓		
ASL	P04424		✓		
PPP2R2A	P63151		✓		
RPL27	P61353		✓		
RAB3GAP2	Q9H2M9		✓		
RPS24	P62847		✓		
PACSIN3	Q9UKS6		✓		
RAP2C	Q9Y3L5		✓		
ATP6V1C1	P21283		✓		
SEZ6L2	Q6UXD5		✓		
SNAP23	O00161		✓		
CAPNS1	P04632		✓		
DHX15	O43143		✓		
HARS1	P12081		✓		
TM9SF1	O15321		✓		
TBC1D8B	Q0IIM8		✓		
PGLS	O95336		✓		
MAPRE3	Q9UPY8		✓		
NANS	Q9NR45		✓		
GPS1	Q13098		✓		
COPS3	Q9UNS2		✓		
SLC38A1	Q9H2H9		✓		
KLC4	Q9NSK0		✓		
PARVA	Q9NVD7		✓		

PTPA	Q15257		✓		
ATP6V1H	Q9UI12		✓		
NIBAN2	Q96TA1		✓		
MOB1B	Q7L9L4		✓		
MOB1A	Q9H8S9		✓		
PRPF8	Q6P2Q9		✓		
PDIA6	Q15084		✓		
SRPRB	Q9Y5M8		✓		
PAICS	P22234		✓		
MAP2K1	Q02750		✓		
PPP2CB	P62714		✓		
PPP2CA	P67775		✓		
BPNT1	O95861		✓		
KRAS	P01116		✓		
RRAGB	Q5VZM2		✓		
RRAGA	Q7L523		✓		
RPL28	P46779		✓		
SRP72	O76094		✓		
RPS6	P62753		✓		
COG4	Q9H9E3		✓		
ARL3	P36405		✓		
LIN7C	Q9NUP9		✓		
PDXP	Q96GD0		✓		
COG5	Q9UP83		✓		
RPS13	P62277		✓		
ACADS	P16219		✓		
LMAN2	Q12907		✓		
PRMT5	O14744		✓		
EFTUD2	Q15029		✓		
LASP1	Q14847		✓		
SFRP1	Q8N474		✓		
OLA1	Q9NTK5		✓		
EPHX1	P07099		✓		
RPS20	P60866		✓		
MON2	Q7Z3U7		✓		
COPZ1	P61923		✓		
CUL1	Q13616		✓		
CDK1	P06493		✓		
PLOD3	O60568		✓		
DNM1L	O00429		✓		
SUCLA2	Q9P2R7		✓		
ARL6IP5	O75915		✓		
EIF3B	P55884		✓		
RAB3GAP1	Q15042		✓		
FGB	P02675		✓		

RPL27A	P46776		✓		
ATP5PO	P48047		✓		
GOLGA7	Q7Z5G4		✓		
CHMP2A	O43633		✓		
RPL10	P27635		✓		
VAMP8	Q9BV40		✓		
GBF1	Q92538		✓		
ATL3	Q6DD88		✓		
RAB25	P57735		✓		
PPP3CC	P48454		✓		
PCK2	Q16822		✓		
ago-01	Q9UL18		✓		
KARS1	Q15046		✓		
RAB22A	Q9UL26		✓		
SURF4	O15260		✓		
ACADVL	P49748		✓		
MMS19	Q96T76		✓		
LYN	P07948		✓		
HCK	P08631		✓		
CLINT1	Q14677		✓		
SEC11A	P67812		✓		
IFIT3	O14879			✓	✓
DYNLRB1	Q9NP97			✓	✓
DYNLRB2	Q8TF09			✓	✓
CLTCL1	P53675			✓	✓
GLRX3	O76003			✓	✓
SERPINA3	P01011			✓	✓
SDF4	Q9BRK5			✓	✓
NACA	E9PAV3			✓	✓
ATP2B2	Q01814			✓	✓
NACA	Q13765			✓	✓
RPLP0P6	Q8NHW5			✓	✓
RPS28	P62857			✓	✓
MPST	P25325			✓	✓
HNRNPR	O43390			✓	✓
SYNCRIP	O60506			✓	✓
CBX3	Q13185			✓	✓
ADAMTS18	Q8TE60			✓	✓
MESD	Q14696			✓	✓
PABPC1	P11940			✓	✓
PABPC3	Q9H361			✓	✓
EEF1E1	O43324			✓	✓
MGAT1	P26572			✓	✓
GSS	P48637			✓	✓
PSMB8	P28062			✓	✓

RPL22	P35268			✓	✓
IFITM2	Q01629			✓	✓
IFITM1	P13164			✓	✓
IFITM3	Q01628			✓	✓
TOLLIP	Q9H0E2			✓	✓
HMGB3	O15347			✓	
ALDH1A3	P47895			✓	
CPNE5	Q9HCH3			✓	
CPNE8	Q86YQ8			✓	
CPNE2	Q96FN4			✓	
CPNE7	Q9UBL6			✓	
CPNE4	Q96A23			✓	
CPNE6	O95741			✓	
CPNE9	Q8IYJ1			✓	
RNH1	P13489			✓	
PDCD5	O14737			✓	
PPP4R2	Q9NY27			✓	
ZC3H14	Q6PJT7			✓	
RUVBL1	Q9Y265			✓	
CKMT1A	P12532			✓	
PFDN2	Q9UHV9			✓	
ARPC5L	Q9BPX5			✓	
XRCC6	P12956			✓	
LGALS3	P17931			✓	
CMPK1	P30085			✓	
TLR2	O60603			✓	
PAFAH1B3	Q15102			✓	
CD276	Q5ZPR3			✓	
CNDP2	Q96KP4			✓	
HMGCS1	Q01581			✓	
NDRG1	Q92597			✓	
THOP1	P52888			✓	
VASP	P50552			✓	
DDX6	P26196			✓	
RRN3P2	A6NIE6			✓	
EIF2S2	P20042			✓	
NMT1	P30419			✓	
SYNE2	Q8WXH0			✓	
NIN	Q8N4C6			✓	
KRT82	Q9NSB4				✓
FLNC	Q14315				✓
VPS26A	O75436				✓
CDV3	Q9UKY7				✓
CBR3	O75828				✓
TAB2	Q9NYJ8				✓

CBR1	P16152				✓
C1QBP	Q07021				✓
SNRPD3	P62318				✓
EIF2S3B	Q2VIR3				✓
RPS5	P46782				✓
RAD23B	P54727				✓
RTN4	Q9NQC3				✓
EIF3K	Q9UBQ5				✓
AHNAK2	Q8IVF2				✓
HOOK2	Q96ED9				✓
CRYAB	P02511				✓
RNPEP	Q9H4A4				✓
CENPF	P49454				✓
CORO1C	Q9ULV4				✓
LARGE1	O95461				✓
TMSB10	P63313				✓
ELAVL1	Q15717				✓
H2AC21	Q8IUE6				✓
H2AC7	P20671				✓
H2AC6	Q93077				✓
H2AC20	Q16777				✓
H2AC11	P0C0S8				✓
H2AJ	Q9BTM1				✓
H2AW	Q7L7L0				✓
H2AC18	Q6FI13				✓
H2AC14	Q99878				✓
H2AC12	Q96KK5				✓
H2AX	P16104				✓
H2AC4	P04908				✓
H2AC1	Q96QV6				✓
NPC1	O15118				✓
BCAS2	O75934				✓
MIF	P14174				✓
FARSA	Q9Y285				✓

Supplementary Table S2. Determination of the presence of the 40 main proteins most frequently identified in exosomes (http://exocarta.org/exosome_markers_new) in exosomes isolated from NuLi - and CuFi corneal stromal cell lines

1	CD9	
2	HSPA8	✓
3	PDCD6IP	✓
4	GAPDH	✓
5	ACTB	✓
6	ANXA2	✓
7	CD63	
8	SDCBP	
9	ENO1	✓
10	HSP90AA1	✓
11	TSG101	
12	PKM	✓
13	LDHA	✓
14	EEF1A1	✓
15	YWHAZ	✓
16	PGK1	✓
17	EEF2	✓
18	ALDOA	✓
19	HSP90AB1	✓
20	ANXA5	✓
21	FASN	
22	YWHAE	✓
23	CLTC	✓
24	CD81	
25	ALB	✓
26	VCP	✓
27	TPI1	
28	PPIA	
29	MSN	✓
30	CFL1	✓
31	PRDX1	✓
32	PFN1	
33	RAP1B	
34	ITGB1	✓
35	HSPA5	✓
36	SLC3A2	✓
37	HIST1H4A	
38	GNB2	
39	ATP1A1	✓
40	YWHAQ	✓

Supplementary Table S3. List of putative exosomal biomarkers detected in exosomes isolated from NuLi and CuFi cell lines cultured in isolation or in contact with *P. aeruginosa* included in classes I to IV

	**NuLi**	**NuLi + *P. aeruginosa***	**CuFi**	**CuFi + *P. aeruginosa***
**Class I**				
SDCBP	7,90[1]	9,77	4,44	9,72
**Class II**				
SLC1A5	4,51		8,25	2,43
SLC3A2	27,07	32,12	31,10	29,18
GNB1	3,38	2,79	11,42	2,43
CLTC	4,51	12,57	52,68	8,51
**Class III**				
CD47	2,25	4,19	3,17	
GNB2	3,38	2,79	11,42	2,43
ITGB1	33,84	20,95	26,02	27,96
BSG	20,30	33,52	10,79	29,18
B2M				
**Class IV**				
ATP1A1	22,56	36,31	38,71	37,69
RAP1B	6,76	9,77	6,34	6,08
GNAI3	2,25	1,39	10,79	1,21

[1]Normalized PSMs

Supplementary Table S4. List of proteins identified by LC-MS/MS in exosomes represented by at least 10 PSMs

		Normalized PSMs			
**Protein symbol**	**Uniprot Entry**	**NuLi**	**NuLi + *P. aeruginosa***	**CuFi**	**CuFi + . *P. aeruginosa***
**FN1**	P02751	382,50	282,14	125,68	207,94
**TF**	P02787	330,60	127,10	10,79	178,76
**HPX**	P02790	244,85	94,98	34,28	164,16
**THBS1**	P07996	196,33	86,60	61,57	86,34
**KRT10**	P13645	152,32	196,94	95,85	200,64
**ALB**	P02769	138,78	215,10	39,35	94,85
**ACTG1**	P63261	72,21	76,82	83,15	79,04
**ACTB**	P60709	71,08	78,22	83,15	85,12
**KRT14**	P02533	66,57	114,53	63,47	103,36
**ACTA1**	P68133	65,44	67,04	0,00	68,10
**ACTA2**	P62736	65,44	67,04	0,00	68,10
**ACTC1**	P68032	65,44	67,04	65,38	68,10
**AGRN**	O00468	64,31	16,76	12,06	21,89
**ACTG2**	P63267	60,93	64,25	0,00	64,45
**LAMC1**	P11047	56,42	9,78	8,25	23,10
**KRT77**	Q7Z794	55,29	71,23	22,22	77,83
**KRT15**	O77727	54,16	86,60	0,00	85,12
**HBB**	P68871	53,03	29,33	13,33	23,10
**HBG2**	P69892	47,39	29,33	13,96	20,67
**HBD**	P02042	47,39	29,33	0,00	20,67
**HBE1**	P02100	47,39	29,33	13,96	20,67
**HBG1**	P69891	47,39	29,33	13,96	20,67
**HSPA8**	P11142	46,26	32,13	34,91	25,54
**LAMA3**	Q16787	46,26	9,78	17,77	14,59
**LAMA5**	O15230	45,13	1,40	28,56	9,73

**HBA1**	P69905	44,00	18,16	20,95	29,18
**LAMC2**	Q13753	41,75	19,55	26,02	25,54
**HSPA5**	P11021	40,62	27,94	38,72	29,18
**KRT19**	P08727	39,49	62,85	32,37	51,07
**PDCD6IP**	Q8WUM4	37,23	29,33	32,37	35,26
**ANXA2**	P07355	37,23	89,39	34,28	72,96
**ALB**	P02768	37,23	72,63	14,60	43,78
**LGALS3BP**	Q08380	37,23	33,52	3,17	41,34
**TGFBI**	Q15582	36,11	13,97	22,22	20,67
**ITGB1**	P05556	33,85	20,95	26,02	27,97
**LAMB1**	P07942	33,85	5,59	11,43	20,67
**YWHAE**	P62258	29,34	18,16	22,85	19,46
**ACTBL2**	Q562R1	28,21	26,54	28,56	25,54
**PRDX1**	Q06830	28,21	32,13	12,69	23,10
**VIM**	P08670	28,21	34,92	15,87	27,97
**SLC3A2**	P08195	27,08	32,13	31,10	29,18
**PSMA7**	O14818	25,95	19,55	4,44	18,24
**PKM**	P14618	25,95	40,51	19,04	36,48
**HSP90AB1**	P08238	24,82	41,90	31,74	30,40
**PTGFRN**	Q9P2B2	24,82	1,40	21,58	1,22
**HSP90AA1**	P07900	24,82	30,73	36,18	24,32
**THBS2**	P35442	24,82	2,79	0,00	1,22
**ITIH4**	Q14624	24,82	0,00	4,44	6,08
**HSPA2**	P54652	23,69	15,36	15,23	15,81
**ATP1A1**	P05023	22,57	36,32	38,72	37,70
**DPYSL2**	Q16555	22,57	12,57	11,43	24,32
**ACTN1**	P12814	21,44	26,54	11,43	25,54
**TAGLN**	Q01995	21,44	1,40	8,89	2,43
**PRDX4**	Q13162	21,44	18,16	6,35	14,59
**POTEE**	Q6S8J3	21,44	34,92	30,47	29,18

**POTEF**	A5A3E0	21,44	34,92	0,00	29,18
**YWHAZ**	P63104	21,44	11,17	18,41	17,02
**VCAN**	P13611	20,31	2,79	0,00	15,81
**ACTN4**	O43707	20,31	26,54	12,06	26,75
**ANXA2P2**	A6NMY6	20,31	55,87	0,00	43,78
**BSG**	P35613	20,31	33,52	10,79	29,18
**DSP**	P15924	19,18	30,73	34,28	36,48
**YWHAH**	Q04917	19,18	13,97	17,14	12,16
**POTEKP**	Q9BYX7	19,18	26,54	0,00	21,89
**Immunoglobulin gamma-1 heavy chain**	P0DOX5	19,18	2,79	2,54	15,81
**YWHAG**	P61981	19,18	15,36	15,87	14,59
**SFN**	P31947	19,18	12,57	20,31	12,16
**IGHG1**	P01857	19,18	2,79	2,54	15,81
**HSPA1B**	P0DMV9	18,05	12,57	13,96	13,38
**TUBA1B**	P68363	18,05	26,54	30,47	17,02
**TUBA1A**	Q71U36	18,05	26,54	0,00	17,02
**PSMA6**	P60900	18,05	9,78	4,44	10,94
**COL7A1**	Q02388	18,05	5,59	5,71	0,00
**YWHAB**	P31946	18,05	9,78	19,04	13,38
**ITGA3**	P26006	18,05	12,57	19,04	10,94
**TUBB**	P07437	18,05	18,16	34,91	21,89
**TUBA1C**	Q9BQE3	18,05	26,54	30,47	17,02
**TUBB4B**	P68371	18,05	15,36	36,18	17,02
**APOD**	P05090	16,92	2,79	0,00	8,51
**PSMA8**	Q8TAA3	16,92	11,17	0,00	10,94
**TUBB2A**	Q13885	16,92	13,97	29,83	15,81
**YWHAQ**	P27348	16,92	11,17	18,41	10,94
**CFI**	P05156	16,92	0,00	0,00	3,65
**TUBB2B**	Q9BVA1	16,92	13,97	28,56	15,81

**TUBA3E**	Q6PEY2	15,80	20,95	0,00	15,81
**ALDOA**	P00883	15,80	25,14	19,04	18,24
**VCL**	P18206	15,80	9,78	16,50	17,02
**FAT2**	Q9NYQ8	15,80	0,00	0,00	0,00
**TUBA3C**	P0DPH7	15,80	20,95	0,00	15,81
**ALDOA**	P04075	15,80	34,92	19,04	25,54
**LAMA1**	P25391	15,80	0,00	0,00	0,00
**IGHA1**	P01876	15,80	8,38	0,00	12,16
**DSG1**	Q02413	14,67	12,57	10,16	20,67
**ITGB4**	P16144	14,67	9,78	36,82	14,59
**HSPA1L**	P34931	14,67	9,78	0,00	12,16
**EFEMP1**	Q12805	14,67	1,40	0,00	0,00
**ATP1A3**	P13637	14,67	13,97	28,56	17,02
**GAPDH**	P04406	13,54	15,36	21,58	12,16
**ANXA6**	P08133	13,54	32,13	36,82	8,51
**HSP90B1**	P14625	13,54	25,14	24,12	15,81
**PARK7**	Q99497	13,54	5,59	2,54	8,51
**ALDOC**	P09972	13,54	6,98	10,16	10,94
**IGKC**	P01834	13,54	8,38	2,54	14,59
**TUBB3**	Q13509	13,54	15,36	32,37	14,59
**IGHG3**	P01860	13,54	1,40	0,00	8,51
**FTH1**	P02794	12,41	13,97	7,62	14,59
**LAMB3**	Q13751	12,41	0,00	9,52	3,65
**PDIA3**	P30101	12,41	11,17	16,50	10,94
**UBC**	P0CG48	12,41	6,98	27,29	9,73
**UBB**	P0CG47	12,41	6,98	27,29	9,73
**UBA52**	P62987	12,41	6,98	27,29	9,73
**RPS27A**	P62979	12,41	6,98	27,29	9,73
**lacZ**	P00722	12,41	13,97	0,00	17,02
**NME2**	P22392	12,41	6,98	7,62	6,08

**DCD**	P81605	11,28	11,17	3,81	12,16
**RAB2A**	P61019	11,28	4,19	7,62	4,86
**P4HB**	P07237	11,28	11,17	12,69	4,86
**LYZ**	P00698	11,28	2,79	0,00	3,65
**AHCY**	P23526	11,28	13,97	17,77	15,81
**CST3**	P01034	11,28	4,19	0,00	3,65
**DES**	P17661	11,28	9,78	4,44	8,51
**NUCB1**	Q02818	11,28	4,19	8,25	7,30
**NME1**	P15531	11,28	5,59	6,35	6,08
**IGHG2**	P01859	11,28	1,40	0,00	7,30
**TUBB4A**	P04350	11,28	11,17	30,47	12,16
**FLNA**	P21333	11,28	26,54	23,49	20,67
**HSPA6**	P17066	11,28	9,78	0,00	9,73
**TIMP1**	P01033	11,28	2,79	0,00	6,08
**TUBB6**	Q9BUF5	10,15	12,57	22,85	10,94
**CFL1**	P23528	10,15	13,97	4,44	14,59
**ALDH1A1**	P00352	10,15	1,40	16,50	7,30
**EZR**	P15311	10,15	15,36	13,33	12,16
**PRDX2**	P32119	10,15	13,97	5,08	7,30
**HSPA7**	P48741	10,15	8,38	0,00	8,51
**COL6A1**	P12109	10,15	1,40	4,44	4,86
**RAN**	P62826	10,15	12,57	10,79	15,81
**ANXA5**	P08758	9,03	32,13	15,87	21,89
**EEF1A1**	P68104	9,03	25,14	20,31	17,02
**EEF1A1P5**	Q5VTE0	9,03	25,14	20,31	17,02
**POTEI**	P0CG38	9,03	22,35	0,00	18,24
**HSP90AB3P**	Q58FF7	9,03	12,57	0,00	10,94
**RDX**	P35241	9,03	12,57	13,33	10,94
**MSN**	P26038	9,03	11,17	21,58	6,08
**TUBB1**	Q9H4B7	9,03	11,17	0,00	9,73

**ITGA6**	P23229	9,03	6,98	29,83	6,08
**HSPG2**	P98160	9,03	8,38	23,49	1,22
**RAB3A**	P20336	9,03	2,79	19,04	13,38
**LTF**	P02788	9,03	2,79	0,00	10,94
**FLNB**	O75369	7,90	26,54	11,43	26,75
**ENO1**	P06733	7,90	25,14	12,06	9,73
**PGK1**	P00558	7,90	18,16	11,43	8,51
**EEF1A2**	Q05639	7,90	18,16	16,50	13,38
**HSP90AA2P**	Q14568	7,90	12,57	0,00	10,94
**ATP1A2**	P50993	7,90	8,38	19,04	10,94
**KRT8**	P05787	6,77	15,36	13,96	21,89
**POTEJ**	P0CG39	6,77	13,97	0,00	10,94
**EEF2**	P13639	6,77	12,57	20,95	21,89
**ATP5F1B**	P06576	6,77	5,59	27,29	4,86
**TUBA4A**	P68366	6,77	8,38	24,12	6,08
**KRT71**	Q3SY84	6,77	9,78	0,00	13,38
**EPHA2**	P29317	6,77	6,98	16,50	10,94
**LDHA**	P00338	5,64	29,33	6,35	17,02
**ITGA2**	P17301	5,64	13,97	17,77	10,94
**EEF1G**	P26641	5,64	9,78	12,69	10,94
**HLA-B**	P01889	4,51	13,97	3,81	8,51
**CLTC**	Q00610	4,51	12,57	52,68	8,51
**FAM149B1**	Q96BN6	4,51	12,57	7,62	0,00
**RAB15**	P59190	4,51	11,17	9,52	8,51
**AHNAK**	Q09666	4,51	8,38	5,71	13,38
**S100A8**	P05109	4,51	6,98	0,00	10,94
**SERPINB5**	P36952	3,38	25,14	5,08	23,10
**VCP**	P55072	3,38	25,14	15,87	19,46
**ANXA1**	P04083	3,38	20,95	10,16	19,46
**LGALS1**	P09382	3,38	18,16	4,44	14,59

**S100A2**	P29034	3,38	12,57	0,00	7,30
**LDHB**	P07195	2,26	11,17	9,52	2,43
**NT5E**	P21589	1,13	15,36	11,43	9,73
**PLEC**	Q15149	0,00	16,76	24,12	10,94
**DYNC1H1**	Q14204	0,00	2,79	97,75	2,43

Supplementary Table S5. List of miRNAs identified by next generation sequencing and represented by at least 1000 copies in exosomes isolated from bronchial epithelial cell lines with and without contact with *P. aeruginosa*.

	Average normalized reads			
**miARN**	**NuLi**	**NuLi + *P. aeruginosa***	**CuFi**	**CuFi + *P. aeruginosa***
hsa-mir-21	410262,66	349571,09	333614,25	313090,84
hsa-miR-21-5p	409628,56	348901,11	333040,49	312670,11
hsa-mir-205	297766,65	273573,78	279264,28	173274,19
hsa-miR-205-5p	297676,07	273425,72	279193,88	173196,45
hsa-mir-16-2	170280,08	186563,06	181336,91	196039,63
hsa-miR-16-5p	170225,64	186525,40	181269,33	195980,15
hsa-mir-16-1	170075,58	186327,17	181122,90	195801,80
hsa-mir-221	100498,89	57588,94	128908,16	77840,53
hsa-miR-221-3p	100170,40	57388,19	128390,72	77666,76
hsa-let-7b	79640,39	70599,77	72979,84	64413,61
hsa-let-7b-5p	79528,30	70496,88	72892,54	64372,47
hsa-mir-92a-1	77799,39	100551,02	67987,09	103157,79
hsa-mir-92a-2	77379,87	99946,28	67457,68	102631,89
hsa-miR-92a-3p	77379,41	99946,28	67457,68	102631,89
hsa-mir-23a	58856,00	38405,17	57465,15	49491,24
hsa-miR-23a-3p	58831,75	38390,10	57441,21	49472,95
hsa-mir-27a	41916,02	27221,12	42374,98	33530,73
hsa-miR-27a-3p	41886,28	27163,42	42288,39	33384,38
hsa-mir-30a	40365,08	32232,24	40023,63	28061,18
hsa-miR-30a-5p	39255,18	31178,32	38812,05	27375,19
hsa-mir-29a	38085,34	27399,28	55154,63	31152,68
hsa-miR-29a-3p	38055,60	27371,68	55120,84	31111,50
hsa-mir-423	26529,72	28872,27	24523,03	32538,38
hsa-mir-320a	25240,02	26515,99	22647,58	28125,20
hsa-miR-320a-3p	25239,56	26490,92	22642,65	28084,06
hsa-mir-24-1	25176,42	23045,62	30419,70	29817,30
hsa-miR-24-3p	25175,96	23043,09	30418,30	29817,30
hsa-mir-24-2	25165,44	23030,56	30391,55	29794,46
hsa-mir-30d	22966,22	22937,70	20884,77	16765,38
hsa-miR-30d-5p	22939,23	22892,54	20863,65	16765,38
hsa-let-7a-3	21446,85	33956,15	33878,44	43495,76
hsa-let-7a-1	21430,38	33928,55	33904,49	43372,31
hsa-let-7a-2	21189,28	33655,03	33567,27	43107,05
hsa-let-7a-5p	21170,06	33617,37	33545,45	43043,02
hsa-mir-148a	20623,34	20581,45	21499,36	33187,77
hsa-miR-148a-3p	20600,01	20553,85	21472,61	33142,01
hsa-mir-93	20282,51	22486,02	21405,73	19202,91
hsa-let-7i	20234,92	21120,96	15442,88	13971,15

hsa-let-7i-5p	20209,30	21085,82	15420,35	13929,97
hsa-miR-93-5p	19930,69	22094,57	21038,95	18896,51
hsa-mir-31	18809,34	12180,26	20811,55	8300,37
hsa-mir-141	18530,73	14937,99	17673,84	21237,95
hsa-miR-31-5p	18521,12	11994,57	20533,47	8172,33
hsa-miR-423-5p	18444,26	19314,25	15577,34	23487,99
hsa-miR-141-3p	18166,09	14521,45	17337,33	20890,42
hsa-mir-25	17676,11	17615,43	16270,07	16669,35
hsa-miR-25-3p	17625,78	17550,20	16211,64	16596,16
hsa-mir-103a-2	17261,61	16666,91	14579,07	10861,35
hsa-mir-103a-1	17233,25	16644,34	14548,09	10797,33
hsa-miR-103a-3p	17232,33	16644,34	14546,69	10797,33
hsa-mir-125b-1	16294,45	21294,11	18822,76	22111,47
hsa-mir-222	15649,82	10027,24	18579,88	14026,01
hsa-mir-125b-2	15647,08	20689,35	18447,54	21567,24
hsa-miR-222-3p	15640,67	10014,70	18560,88	14007,75
hsa-miR-125b-5p	15535,45	20508,68	18281,39	21416,31
hsa-mir-151a	14909,13	13931,75	16038,46	14730,29
hsa-mir-26a-2	14888,54	18910,26	17150,07	18164,79
hsa-mir-26a-1	14887,62	18917,77	17148,66	18155,62
hsa-miR-26a-5p	14875,73	18905,23	17137,39	18146,49
hsa-miR-151a-3p	14681,75	13733,52	15792,06	14506,22
hsa-mir-200c	13151,40	13091,12	17210,61	16550,43
hsa-miR-200c-3p	13057,15	13003,29	17121,91	16468,11
hsa-mir-27b	12573,57	13793,75	11137,93	13897,96
hsa-miR-27b-3p	12570,83	13788,72	11135,82	13893,42
hsa-mir-100	12377,76	9284,50	10774,67	5538,15
hsa-miR-100-5p	12368,61	9261,91	10763,41	5519,85
hsa-mir-30e	10894,53	13472,54	10985,17	13367,48
hsa-miR-30e-5p	10714,28	13259,25	10795,09	13276,03
hsa-mir-26b	10693,23	12684,61	13252,04	14309,56
hsa-miR-26b-5p	10679,96	12672,07	13237,25	14309,56
hsa-miR-320b	9895,34	16275,46	9667,99	17090,08
hsa-mir-320b-2	9895,34	16275,46	9667,99	17090,08
hsa-mir-320b-1	9874,76	16240,33	9646,87	17044,36
hsa-mir-20a	9661,56	8564,31	9736,28	8730,23
hsa-miR-20a-5p	9643,26	8554,28	9722,90	8711,97
hsa-miR-320c	9355,49	15673,22	9132,25	16491,00
hsa-mir-320c-2	9355,49	15673,22	9132,25	16491,00
hsa-mir-320c-1	9322,55	15643,12	9107,61	16463,53
hsa-mir-181b-2	9002,76	8872,96	6830,88	5048,81
hsa-miR-181b-5p	8986,29	8850,37	6826,66	5044,23
hsa-mir-181b-1	8767,14	8632,07	6704,16	4993,96
hsa-mir-378a	8732,83	8890,53	9443,42	8602,19
hsa-miR-378a-3p	8638,58	8750,00	9278,68	8492,44

hsa-let-7f-2	8440,48	18300,49	9767,95	15388,82
hsa-let-7f-5p	8423,55	18287,93	9726,42	15370,56
hsa-mir-23b	8335,71	3646,05	5854,44	4765,30
hsa-let-7f-1	8322,45	18057,07	9565,21	15096,17
hsa-miR-23b-3p	8310,10	3598,36	5836,14	4756,13
hsa-miR-423-3p	8085,00	9558,00	8945,69	9050,38
hsa-mir-503	6781,58	3957,20	1731,83	1513,74
hsa-miR-503-5p	6733,99	3924,57	1712,82	1504,57
hsa-mir-320d-1	6574,33	12293,16	6573,22	12384,22
hsa-miR-320d	6548,25	12250,50	6555,62	12365,97
hsa-mir-320d-2	6548,25	12250,50	6555,62	12365,97
hsa-mir-361	6526,75	5241,97	6398,63	5186,03
hsa-mir-181a-2	5813,50	5385,00	4415,47	3032,02
hsa-miR-361-5p	5791,54	4466,58	5574,25	4472,61
hsa-mir-155	5728,40	4343,65	5976,93	3210,38
hsa-miR-155-5p	5728,40	4343,65	5976,93	3210,38
hsa-mir-22	5559,58	4725,06	5648,16	6718,02
hsa-mir-125a	5465,34	12350,89	6289,51	15530,62
hsa-miR-22-3p	5295,14	4599,58	5400,36	6567,14
hsa-miR-125a-5p	5273,65	12089,90	6114,92	15297,37
hsa-mir-424	5076,91	3317,33	1525,56	1317,09
hsa-mir-101-2	4976,26	5723,75	4151,47	5218,04
hsa-miR-101-3p	4976,26	5723,75	4150,77	5218,04
hsa-mir-128-1	4957,97	5036,21	4423,22	6178,42
hsa-mir-101-1	4891,17	5615,86	4080,36	5158,56
hsa-mir-128-2	4718,23	4697,44	4169,77	5876,56
hsa-miR-128-3p	4718,23	4697,44	4169,77	5876,56
hsa-miR-424-5p	4559,03	2968,52	1388,99	1193,63
hsa-mir-425	4429,09	4757,67	5548,90	4614,37
hsa-mir-148b	4371,44	5199,31	4675,95	6978,70
hsa-mir-191	4365,95	6308,43	7364,51	6265,28
hsa-miR-148b-3p	4350,86	5176,74	4634,41	6900,96
hsa-mir-181a-1	4339,42	4752,66	3803,70	2780,52
hsa-miR-191-5p	4312,89	6238,16	7295,52	6160,12
hsa-mir-200a	4163,28	4752,66	5273,64	5963,47
hsa-mir-19b-1	4162,37	3332,37	5465,13	3489,35
hsa-mir-19b-2	4154,13	3317,33	5460,90	3484,76
hsa-miR-19b-3p	4154,13	3317,33	5460,90	3484,76
hsa-mir-12136	4137,21	14656,94	10471,95	22532,20
hsa-miR-107	4118,45	2677,44	3403,12	2583,87
hsa-mir-107	4118,45	2677,44	3403,12	2583,87
hsa-miR-200a-3p	4112,96	4704,97	5228,59	5936,04
hsa-miR-181a-5p	4108,84	4544,38	3572,79	2629,59
hsa-miR-425-5p	4042,96	4283,42	5041,32	4170,76
hsa-miR-12136	3955,12	14656,94	10471,95	22532,20

hsa-mir-138-1	3388,73	2112,86	3603,06	2318,61
hsa-mir-183	3257,88	4002,37	4830,83	4879,63
hsa-miR-183-5p	3249,19	3994,84	4818,16	4875,04
hsa-mir-186	3214,42	2812,96	3621,36	2780,52
hsa-miR-186-5p	3212,59	2810,43	3618,54	2766,80
hsa-miR-138-5p	3203,90	1949,74	3444,65	2176,85
hsa-let-7e	2929,40	3417,70	3901,55	4097,61
hsa-let-7e-5p	2909,27	3382,57	3874,80	4074,72
hsa-mir-1307	2784,82	3460,36	2228,15	2693,61
hsa-mir-28	2690,12	2040,07	3205,30	2496,96
hsa-miR-28-3p	2656,26	2027,53	3161,65	2419,22
hsa-miR-1307-3p	2579,86	3026,25	1976,82	2378,09
hsa-mir-146a	2555,61	1146,77	145,02	201,24
hsa-miR-146a-5p	2555,16	1146,77	145,02	201,24
hsa-mir-92b	2433,01	3101,52	2433,72	3251,56
hsa-mir-342	2421,11	3317,33	2647,03	3873,53
hsa-miR-342-3p	2416,08	3314,81	2639,99	3864,36
hsa-mir-335	2379,48	4398,85	2473,85	7577,78
hsa-miR-92b-3p	2323,66	2973,55	2339,38	3068,62
hsa-mir-34a	2320,00	2511,84	1298,17	1614,36
hsa-mir-7-1	2318,63	12970,69	2629,42	20442,22
hsa-let-7g	2318,63	3013,69	2987,06	3530,53
hsa-mir-7-3	2313,14	12948,09	2689,97	20442,22
hsa-miR-34a-5p	2313,14	2506,81	1296,06	1614,36
hsa-mir-138-2	2312,68	1382,63	2212,66	1568,59
hsa-let-7g-5p	2310,39	3008,68	2982,13	3521,36
hsa-miR-7-5p	2299,87	12958,13	2613,94	20423,92
hsa-mir-7-2	2298,04	12958,13	2612,54	20401,08
hsa-mir-200b	2245,88	4737,60	3009,59	7925,36
hsa-miR-335-5p	2132,88	4087,69	2160,56	7170,77
hsa-miR-200b-3p	2108,64	4619,67	2867,38	7783,61
hsa-mir-99b	2085,76	2245,85	2307,00	1929,89
hsa-mir-29c	2035,89	5502,93	2830,07	9763,80
hsa-miR-29c-3p	2013,02	5477,86	2793,46	9722,62
hsa-mir-29b-1	1927,01	2436,55	3496,75	3388,73
hsa-mir-193a	1927,01	1545,75	1556,54	1852,15
hsa-miR-193a-5p	1919,69	1535,70	1546,68	1852,15
hsa-mir-29b-2	1910,54	2436,55	3472,11	3370,43
hsa-miR-29b-3p	1903,22	2418,98	3465,07	3365,89
hsa-mir-130a	1822,24	1528,19	1598,78	1788,13
hsa-miR-130a-3p	1817,66	1528,19	1597,37	1788,13
hsa-mir-17	1802,57	983,65	1507,25	1125,02
hsa-mir-182	1798,45	3129,12	2827,25	4230,24
hsa-miR-182-5p	1785,18	2579,58	2789,94	3795,75
hsa-mir-106b	1760,47	1432,83	2009,21	1001,52

hsa-miR-17-5p	1749,49	956,05	1414,33	1097,55
hsa-let-7c	1732,11	3121,61	3204,59	4440,60
hsa-let-7c-5p	1730,73	3116,58	3201,78	4436,02
hsa-mir-152	1719,30	2107,83	1779,70	2533,56
hsa-miR-152-3p	1716,56	2107,83	1779,00	2533,56
hsa-miR-181a-2-3p	1699,63	830,59	826,49	393,30
hsa-miR-99b-5p	1681,78	1816,75	1912,06	1719,52
hsa-miR-106b-3p	1657,53	1350,00	1907,83	932,95
hsa-mir-132	1640,61	1508,10	921,53	1847,56
hsa-mir-15b	1587,54	1417,76	2211,25	1486,27
hsa-miR-132-3p	1568,78	1427,80	849,02	1746,95
hsa-let-7d	1561,00	1588,39	837,75	978,68
hsa-mir-429	1500,16	3076,42	2164,09	5039,69
hsa-miR-429	1499,70	3076,42	2164,09	5039,69
hsa-mir-532	1496,50	1360,06	2077,49	1564,05
hsa-mir-224	1489,63	1824,28	3234,16	3064,03
hsa-miR-224-5p	1489,63	1824,28	3233,46	3064,03
hsa-miR-532-5p	1438,85	1282,27	1990,20	1495,44
hsa-mir-708	1418,26	1954,77	2883,57	2853,67
hsa-miR-708-5p	1388,52	1924,64	2830,77	2794,23
hsa-mir-660	1367,48	1327,43	1821,94	1289,66
hsa-miR-660-5p	1363,82	1327,43	1819,13	1289,66
hsa-miR-15b-5p	1211,47	1041,38	1748,73	1134,15
hsa-mir-584	1155,65	963,58	2139,45	2739,34
hsa-miR-584-5p	1154,28	963,58	2136,63	2734,79
hsa-miR-30a-3p	1108,99	1053,91	1211,58	685,99
hsa-mir-185	1024,35	777,89	1307,32	836,88
hsa-let-7d-5p	1016,11	1031,32	620,92	685,99
hsa-miR-185-5p	1001,02	750,29	1288,31	809,45
hsa-mir-339	908,60	1605,96	1627,64	1513,74
hsa-mir-19a	906,32	782,90	1124,28	955,79
hsa-miR-19a-3p	904,94	780,40	1122,17	946,67
hsa-mir-345	893,51	1084,02	1532,60	1294,20
hsa-miR-345-5p	893,05	1081,51	1527,67	1275,91
hsa-mir-126	885,27	1189,41	1105,27	1468,02
hsa-mir-629	855,07	1056,42	1127,10	1582,35
hsa-miR-629-5p	847,75	1053,91	1119,35	1564,05
hsa-mir-454	811,61	988,68	1403,06	1134,15
hsa-mir-192	797,43	1006,25	869,44	1614,36
hsa-miR-192-5p	796,06	1001,22	868,03	1596,06
hsa-miR-1246	777,30	3846,78	765,25	5135,72
hsa-mir-1246	777,30	3846,78	765,25	5135,72
hsa-miR-454-3p	711,42	777,89	1249,60	1001,52
hsa-mir-210	690,83	552,04	1076,41	585,37

hsa-miR-210-3p	684,88	544,51	1070,78	553,36
hsa-miR-339-5p	617,17	1390,16	1263,68	1230,18
hsa-mir-375	456,59	4175,52	480,12	6754,62
hsa-miR-375-3p	455,22	4173,00	480,12	6754,62
hsa-mir-199b	414,95	915,89	261,89	1303,37
hsa-mir-199a-2	401,23	898,33	253,44	1298,79
hsa-mir-199a-1	401,23	878,26	253,44	1294,20
hsa-miR-199a-3p	400,32	873,25	251,33	1266,78
hsa-miR-199b-3p	400,32	873,25	251,33	1266,78
hsa-mir-203a	347,24	2910,82	835,64	2163,13
hsa-miR-203a-3p	345,87	2898,26	832,13	2153,96
hsa-mir-6724-1	304,24	639,87	626,56	1074,71
hsa-mir-6724-2	304,24	639,87	626,56	1074,71
hsa-mir-6724-3	304,24	639,87	626,56	1074,71
hsa-mir-6724-4	304,24	639,87	626,56	1074,71
hsa-mir-9-1	298,75	388,95	1389,69	1806,43
hsa-mir-9-2	298,75	388,95	1389,69	1797,26
hsa-mir-9-3	298,75	386,44	1389,69	1792,71
hsa-miR-1275	231,50	883,29	464,64	2140,25
hsa-mir-1275	231,50	883,29	464,64	2140,25
hsa-miR-9-5p	204,96	291,08	1078,52	1417,71
hsa-mir-143	194,90	1688,78	188,67	3370,43
hsa-miR-143-3p	193,98	1686,25	187,97	3356,72
hsa-mir-219b	128,56	2017,50	66,88	1714,93
hsa-mir-376c	116,20	850,66	198,53	1550,34
hsa-miR-376c-3p	116,20	850,66	197,82	1550,34

Supplementary Table S6. Prediction of the biological targets of miRNAs whose expression appears altered in exosomes from bronchial epithelial cell lines with and without contact with *P. aeruginosa*. Colors indicate the number of genes potentially regulated by a miRNA. dark blue: 7; red: 6; purple: 5; green: 4; brown: 3; light blue: 2; black: 1

**Downregulated miRNA**	**Regulated Genes**
**Genes potentially regulated by miRNAs whose levels are increased in CuFi and Nuli**	
**hsa-miR-12136**	*NUFIP2, PRKG1, ZBTB20, BRWD3, DDX3X, FSTL1, MBNL3, MIER3, PTAR1, SESN3, AHR, ATL2, ATXN1, CASK, CNOT6, CTDSPL2, DGKH, EGLN1, FRMD4B, GPM6A, INO80D, MAP3K2, NFIA, PPP1R9A, RFX7, SLC4A7, STRN, TMEFF2, ZNF704, ARID4B, ATXN7, BNIP2, C5orf24, CACNB4, CADM2, CCNT2, CEP97, COL19A1, CREB1, CYTH3, DNMT3B, DPYSL2, EIF5A2, ETS1, FBXO28, GAB1, GLCE, GMFB, GUCY1A2, HOOK3, MAP4K3, MBNL2, MMD, MYEF2, NRXN1, OSBPL8, PGAP1, PHTF2, PIK3CA, PTP4A1, RAP2C, RBMS3, RC3H1, RNF138, ROBO2, SECISBP2L, SEL1L, SMNDC1, STX16, TAOK1, THSD7A, TMEM65, TNPO1, TRIM33, UBE2R2, ULK2, USP37, YTHDF3, ZC3H6, ZFHX3, ZMYM2, ABCB1, ABHD13, ACVR1C, AEBP2, ALDH1L2, ARHGAP12, ARL5A, ATAD1, ATP11C, BAZ1A, BCL11B, C11orf87, CBX5, CCDC50, CCDC88A, CCSER1, CDC42SE2, CDC73, CNTLN, COL25A1, CORO1C, CPEB2, CPED1, CREBRF, CTBP2, DAAM1, EEA1, EFNB2, ELAVL2, ETNK1, EVI5, EXD1, FAM168B, FAM19A2, FAT4, FBXO33, FBXO9, FGF14, FGFR1OP2, FGL2, GIT2, GNAQ, GPC4, GPR137B, HDAC9, HIPK1, HIPK2, HIPK3, HMBOX1, HMGCLL1, HOOK1, IPMK, ITGB1, KCNK2, KDM4C, KLF6, KLF8, KLHL15, KLHL24, LRRC58, MAGT1, MAPK1IP1L, MED13, MEX3D, MGAT4A, MYNN, NAV1, NEK7, NETO2, NFAT5, NFE2L2, NOTCH1, NUCKS1, OCLN, ONECUT2, PAG1, PARP8, PDLIM5, PEX3, PFDN4, PHC3, PIWIL1, PLXNC1, PPFIA1, PPP3CA, PRKACB, PSD3, PTGFRN, PUM2, RALGPS2, RBFOX2, RBPJ, RDH10, REEP3, RND3, RNF2, RORA, RTKN2, SCAMP1, SEC24A, SETD2, SGIP1, SGMS2, SH3GLB1, SIX4, SKIL, SLAIN1, SLC17A6, SLC19A2, SLC35A3, SLC38A2, SLC39A14, SLC4A4, SMIM14, SP1, SSBP3, STAM2, STXBP5, SUZ12, SYT14, TAL1, TBL1XR1, TCF4, TM9SF2, TM9SF3, TMEM181, TMEM64, TMX3, TP53INP1, TTC30B, TTPA, UBE2W, USP25, VGLL3, ZC3H12C, ZEB2, ZFHX4, ZFP36L1, ZMAT3, ABCG2, ACPP, ACVR2A, ADAM22, ADD3, ADK, ANAPC16, ANKRD12, ANKRD27, ANKRD50, APAF1, ARL13B, ARL8B, ARPP19, BACH2, BAG4, C6orf47, CAMK4, CDC42BPA, CDK19, CDK6, CHIC1, CLOCK, CNOT6L, COL11A1, CPEB3, CPNE4, CRLF3, DCP2, DCUN1D4, DCX, DLX5, DTWD2, ECHDC1, EFR3A, EGR3, EIF2AK3, ELMSAN1, ELOVL5, EOGT, ETV1, FAM126B, FGD4, FRS2, GABRA1, GRB10, GRIP1, HDAC4, IDE, IGSF11, IKZF2, JAG1, KCNA1, KCTD12, KCTD5, KDM5A, KIAA1211, KLF9, KMT2E, KPNA1, LCA5, LIMCH1, MBTD1, MDFIC, MED14, MTF1, MYO6, MYOT, NAMPT, NEDD4L, NEUROD1, NOTCH2, NR4A3, NT5E, NUDT21, NXT2, PAK2, PAN3, PCDHA1, PCDHA10, PCDHA11, PCDHA12, PCDHA13, PCDHA2, PCDHA3, PCDHA4, PCDHA5, PCDHA6, PCDHA7, PCDHA8, PCDHAC1, PCDHAC2, PDAP1, PEX5L, PMP22, PPAT, PRKAR2B, PRKCI, PRPF4B, PRR14L, PRR3, PRRC1, PRRC2C, PTBP3, PTPRK, RAB14, RAB30, RAD21, RERE, RFX3, RSBN1, RYBP, SGTB, SIRT1, SKIDA1, SLC5A3, SLC7A11, SLC7A2, SMIM17, SMPD3, SNX18, SORBS2, SOX21, SOX6, SP3, SPTBN1, SPTY2D1, SRF, ST8SIA4, STX17, TENM1, TFAP2C, TFDP1, TLL1, TMEM120B, TMEM135, TMEM236, TNRC18, UBE2D3, UBR3, VAPA, ZBTB44, ZBTB7A, ZFP36L2, ZIC3, ZNF148, ZNF608, ZNF652, ZNRF2, 37104, 38200, 39326, 40422, AAK1, ABCA1, ABCB10, ABHD18, ACAP2, ACER3, ACSL6, ACTL6A, ACTR3, ACVR1, ACVR2B, ADAM17, ADAMDEC1, ADCY7, ADGRB3, AGTR2, AHCTF1, AHDC1, AKAP1, AMOT, ANKRD10, ANKRD13A, ANKS1B, ANXA4, AP1S3, AP3B1, AP4E1, APPBP2, APPL1, AREG, ARF3, ARGLU1, ARHGEF26, ARL5B, ARMCX3, ASB8, ASH1L, ATP13A3, ATP6AP2, ATP6V1C1, ATXN2L, AVL9, B3GALNT1, B3GALT2, B3GLCT, BAG2, BCAT1, BCL10, BCL11A, BCL2L11, BCL2L2, BICRAL, BMP3, BRAF, BRD3, BRPF3, BTBD7, BTC, C18orf25, C21orf91, C2CD3, C3orf70, C3orf80, C5orf63, C9orf64, CA13, CACNA2D3, CACUL1, CALU, CAMLG, CARF, CAV1, CCBE1, CCDC148, CCDC158, CCDC34, CCNA2, CCP110, CD24, CDC5L, CDCA4, CDH7, CDKL5, CDV3, CEBPB, CELF4, CHD1, CHD6, CHST9, CKAP5, CLCC1, CLCN3, CLEC12B, CNTNAP2, COA5, COL12A1, CPEB1, CPNE3, CPNE8, CRY1, CSNK1G1, CSPG5, CSRNP3, CUL5, CXCL14, CXCL3, CXCL9, CXCR4, CYB5R4, CYBRD1, CYSLTR1, DBT, DCUN1D1, DDX60, DENND1B, DERL1, DESI2, DHRS7, DIPK2B, DKK2, DNAH10, DNAH12, DNAH8, DNAJB4, DNAJC15, DOCK4, DPYS, DSC3, DUSP8, DUT, DYNC1LI2, DYRK3, EBF1, ECPAS, ECT2, EEF1A1, EHMT1, EIF4E3, ELAVL3, ELP4, EMC8, EPB41L5, EPCAM, EPHA7, ERC2, EREG, ERICH2, ERO1B, EYA1, EZH2, FABP7, FAM102B, FAM135A, FAM155A, FAM160A2, FAM160B1, FAM161A, FAM172A, FAM199X, FAM76B, FAM98A, FBXO22, FBXO32, FBXO42, FECH, FKBP5, FLRT3, FLVCR1, FNDC3A, FNIP2, FOXP1, FSTL5, FZD3, FZD6, G0S2, GABRB3, GALNT13, GALNT4, GASK1B, GATA2, GBF1, GDNF, GK, GK5, GNA13, GNAI2,*

	GNL3L, GOLGA3, GOLPH3, GPR137C, GPR183, GRIA2, GRIK2, GRM5, GRTP1, GSK3B, GSPT1, GTF2A2, GTF2H1, GTF3C3, GUCY1B1, HACD4, HECW2, HERC4, HEXIM1, HFM1, HLTF, HNRNPA2B1, HNRNPH3, HNRNPU, HNRNPUL1, HOMER1, HSPE1-MOB4, ICE2, ICK, ID2, ID3, IDH3A, IFNGR2, IGFBP1, IGFBP7, IL22RA2, IL26, IL5RA, IL6ST, ILF3, IMPAD1, ING5, INPP5A, INTS2, IRAK1BP1, IRF1, IRF2BP2, IRX5, IVNS1ABP, JADE3, KANK1, KARS, KAT2B, KATNBL1, KCNN3, KCNQ5, KCNT2, KCTD18, KDSR, KHDC4, KHSRP, KIF5B, KITLG, KLHDC1, KMT5B, KMT5C, KPNA4, LARS, LATS2, LCOR, LDB2, LDB3, LHFPL3, LHX9, LIN54, LMBR1, LNPK, LPAR1, LRCH2, LRIT3, LRP8, LRRC49, LRRN1, LSM8, LUC7L3, LUZP2, LY75, LYRM7, MAF, MAGI3, MAML1, MAP3K20, MAPK1, MARCKSL1, MAST3, MBD2, MBTPS2, MCM6, MCUR1, MDM4, MED13L, MEF2A, MEGF11, MFN1, MFSD14B, MFSD8, MICU3, MID2, MIGA1, MKX, MMS22L, MOB1B, MOB4, MOSPD1, MPP6, MRPL19, MTA1, MTM1, MTURN, MYLIP, MYLK, MYT1L, MZT1, NAA25, NAALADL2, NADK, NADK2, NAGA, NCAM1, NCKAP1, NCL, NCOA3, NECTIN3, NELFA, NEMP2, NEO1, NEURL1B, NF1, NFIB, NOL4L, NOTUM, NPTN, NR2C2, NR4A2, NRARP, NRIP1, NTRK2, NUB1, NUDCD1, NUP205, NXF1, OAZ1, OGFRL1, OGN, OMA1, OSBPL2, OSBPL3, OSBPL6, OSGIN2, OTUD7B, OTULIN, PABPC4, PAFAH1B2, PAK5, PARD3B, PARD6B, PARP12, PCDH15, PCDH20, PCDHB9, PCF11, PCLO, PCNX1, PCYT1B, PDCL, PDE4B, PDSS1, PECR, PELI2, PFKFB4, PHACTR2, PHIP, PHLDB1, PIAS3, PIGK, PKIG, PLAC8, PLCXD3, PLEKHJ1, PLK2, PLOD2, PLPPR4, PLPPR5, PLSCR4, PM20D2, PNISR, POC1B-GALNT4, POLH, POLR2E, POLR2H, POTEF, POU2F1, PPEF2, PPIL4, PPIP5K2, PPM1A, PPM1D, PPP1CC, PPP1R2, PPP1R21, PPP1R27, PPP2R2A, PPP6C, PRDX6, PREX2, PRKAA1, PRKAA2, PRKD3, PRND, PROK2, PROM1, PROSER1, PRPF39, PRRG3, PRRG4, PRTFDC1, PRTG, PSMA2, PSMD11, PSMD5, PTPN2, PTPRE, PTPRG, PTRH1, PVR, PYROXD1, RAB5B, RAB5IF, RAG1, RAI14, RAPH1, RB1CC1, RBM27, RBM39, RBM41, RBM6, RBMS2, RC3H2, RCBTB2, RCHY1, RELCH, RELL1, RETREG1, RHBDL2, RHPN2, RIC1, RNF146, RNF212B, RNF8, RPS6KA5, RPS6KA6, RSPH4A, RUNDC3B, RUNX1, RUNX2, RWDD4, RXYLT1, SAMD12, SAMD8, SAR1A, SAR1B, SAV1, SCAF4, SCAI, SCAP, SCML1, SCN2A, SCN7A, SCN9A, SDK1, SEC31A, SEC61A2, SEC61B, SERBP1, SERPINE2, SERTAD2, SF3A1, SF3A3, SFMBT1, SFRP5, SGMS1, SH3GLB2, SHISA3, SIM2, SIMC1, SIN3A, SINHCAF, SKI, SLAIN2, SLC11A2, SLC20A2, SLC2A13, SLC30A4, SLC30A7, SLC35D1, SLC35E2A, SLC35F1, SLC35F3, SLC6A15, SLC9B1, SLFN5, SMAD1, SMAD2, SMAD5, SMARCAD1, SMARCE1, SMC6, SMG1, SNW1, SNX16, SNX9, SOCS4, SOCS6, SON, SORCS1, SORCS3, SOS2, SOX5, SOX8, SPATA6, SPPL2A, SPRED1, SPRED2, SRSF10, SRSF2, SS18, SSH1, SSH2, SSR1, ST13, STAM, STAT3, STAT5B, STK17B, STK35, STRBP, SUB1, SUMO1, SUMO2, SUPT3H, SYNE3, SYNJ2BP, SYNM, SYT4, TAB3, TAF2, TASOR, TBR1, TCEA1, TCEAL8, TENT2, TENT4B, TFDP3, TGDS, TJP1, TM9SF1, TMA16, TMED7-TICAM2, TMEM127, TMEM150A, TMEM170A, TMEM196, TMEM206, TMEM56, TMEM67, TMEM68, TMEM87A, TMPO, TMPRSS11D, TNFAIP6, TNRC6B, TNRC6C, TOB2, TOLLIP, TOX, TOX3, TPGS2, TPST2, TRAK2, TRIM36, TRIM65, TRIO, TRIP11, TRPM7, TRPM8, TRPS1, TSHZ1, TSPAN2, TSPAN9, TTC14, TTC28, TTC3, TTC30A, TTC32, TUBE1, TULP4, TUSC3, TUT4, TUT7, TWF1, TWSG1, TXN, UBE2B, UBE2D1, UBE2H, UBE2K, UBE4A, UBQLN1, UBR2, UBR5, UNC5D, UNK, USF3, USH2A, USP12, USP24, USP44, USP46, USP9X, UTP25, VCAN, VEZF1, VIM, VWC2, WDR26, WIZ, WWTR1, XPO4, XPOT, XPR1, XRN1, YAP1, YES1, YTHDC2, YTHDF1, YTHDF2, YWHAQ, ZBTB2, ZBTB25, ZBTB6, ZCCHC18, ZCCHC3, ZCCHC8, ZDHHC20, ZDHHC3, ZFAND4, ZFC3H1, ZHX1, ZMYM3, ZNF112, ZNF236, ZNF322, ZNF326, ZNF34, ZNF362, ZNF423, ZNF503, ZNF678, ZNF713, ZNF714, ZNF737
**hsa-miR-1246**	DDX3X, MIER3, ANTXR2, GMFB, SLC12A2, TAF9B, ESM1, RTKN2, SCEL, SLC38A2, ADAM22, DYRK1A, EIF2AK3, GPR85, KDM5A, KLHL14, MYOT, NDFIP1, RAB14, REPS2, SORBS2, ACE2, ADAT2, ANKDD1B, ATP2B1, C12orf71, C15orf53, CDO1, COL6A6, CREBL2, DLG1, DUSP18, DYNC1I1, ELF5, FAM45A, FAM53C, FPGT, GLRB, HECTD2, HLF, HTR5A, ISCA1, LYPD1, MIER1, MRPS14, PANX1, PLEKHB2, SCN3A, SEPHS1, SHISA9, TBCK, TNFRSF8, ZCCHC14, ZNF425
**hsa-mir-125a**	NUFIP2, ZNF704, CREB1, DACH1, LARP4, PEAK1, TAOK1, BNC2, FXR1, KDM4A, MAPK9, SGK1, ACPP, CHIC1, CPNE4, KLF9, MAP3K12, MAPK10, NLK, NSG2, RGS7BP, ANKRD45, ARL1, BAZ2A, BOK, BRCA1, CCDC178, CERS5, CHGB, CNOT4, CPA3, CUX2, CYB561D1, DHX57, DTWD1, EMC1, FUT6, GLG1, GMNC, GPRC5A, HEYL, HTR4, IFIT2, IGF2BP2, INHBC, INMT, ITGAV, JMJD8, KRTAP1-1, LAIR1, LIMD1, MAB21L2, MEAF6, MTAP, MTFR1, NARS, NIT1, OVOL1, OXLD1, PCDH8, PGM3, POP1, PRDM5, PRKAG1, PTCH1, RNF185, S100A7A, SEC14L2, SLC9A7, STAP1, TDRP, THUMPD3, VPS39, WDFY2, ZNF287
**hsa-miR-125a-5p**	ATXN1, INO80D, ZNF704, BNIP2, CBFB, CCNJ, DICER1, ETS1, GJC1, NRXN1, SEL1L, TAF9B, UBE2R2, BAP1, BTG2, DAAM1, DENND6A, EIF2B5, HAPLN1, KIAA1841, LCLAT1, MBOAT2, PHACTR3, PI4K2B, PRDM1, RBM7, RORA, SLC4A4, SYVN1, TRAF6, TTPA, ZSWIM4, ABHD3, ANAPC16, ANKRD50, BAG4, BAK1, BMF, C6orf47, CDK19, DIRAS1, DTX4, GGA2, GRB10, IGSF11, KCNA1, KCNK10, KIAA0319L, MAP3K11, MAPRE2, MCL1, MTF1, ORC2, PPAT, PRRC1, RAP1A, RBAK, REST, RFX3, RHOQ, RYBP, SEMA4C, SH3BP5L, SLC25A15, SLC39A9, SNX18, SRF, TDG, TMEM120B, TMEM135, TNFAIP3, UBN1, ZBTB7A, ZNF652, ZNRF3, ABHD6, ABTB1, ACER2, ACHE, AIFM1, ALPK3, ANKRD33B, ARID3B, ASIC1, ATP10D, BLZF1, C19orf38, C1orf210, CACNB3, CDC37L1, CDC42BPG, CDC42SE1, CDH5, CDR2L, CEP85, CGN, CHTF8, CPSF6, CRB2, CYP24A1, DHX33, DIS3L2, DOCK3, DPH2, DRAM2, DUS1L, DUSP6, E2F2, EBF4, EIF1AD, EIF4EBP1, ENPEP, ENPP1, ESRRA, EVA1A, FAM118A, FAM169B, FBXW4, FRMD5, FUT4, GALNT14, GCNT1, GOLGA5, HCN3, HIC2, HIF1AN, HINFP, IER2, IER3IP1, IL16, IL6R, INTS7, IRF4, IST1, ITGA8, KCNIP3, KCNS3, KCTD15, KCTD21, KHNYN, KIAA1522, KLC2, KLF13, LBH, LFNG, LGI2, LIN28A, LIN28B, LIPA, LRP4, LRRC10B, LRRC8B, M6PR, MAMDC2, MAN1B1, MAP3K10, MAP3K9, MAPK12, MFHAS1, MFSD9, MKNK2, MMP11, MORC2, MSI1, MSRB3,

	MXD4, MYO18A, MYT1, NBEAL2, NCAN, NCKAP5L, NCOR2, NECAB3, NEU1, NIPA1, NIPAL4, NPL, NR6A1, NT5DC1, NUP210, OLFML2A, OSBPL9, PAFAH1B1, PCTP, PDE7A, PDK3, PHF20, PLEKHA8, PLEKHM3, PMM2, PPME1, PPP1R37, PPP2R5C, PRSS35, QSOX2, RAB3D, RABEP2, RAPGEF5, RASGEF1A, RASGRF1, RASGRF2, RASSF3, RBM20, RFXANK, RPS6KA1, RREB1, RUFY3, SAMD10, SARM1, SBNO1, SCARB1, SCLY, SCN2B, SEMA4B, SEMA4D, SEMA4F, SERTAD3, SGPL1, SH3TC2, SLC16A6, SLC25A35, SLC26A6, SLC35A4, SLC38A9, SLC46A3, SLC6A17, SLC7A1, SLITRK6, SMURF1, SSTR3, STARD13, SULT4A1, SUN1, SUV39H1, TAZ, TBC1D1, TBC1D16, TBX4, TLE3, TMEM132E, TMEM161B, TMEM168, TMTC2, TNFSF4, TOMM40, TOR2A, TRIAP1, TRIM71, TSEN54, TSNARE1, TTC7A, UBE2G1, UBR7, UCK2, ULK3, USP2, USP38, VCPIP1, VDR, VPS36, VPS4B, VTCN1, WARS, XKRX, ZBTB37, ZFYVE1, ZKSCAN5, ZNF385A, ZNF691, ZNF827, ZSCAN29, ZSWIM5, ZSWIM6
**hsa-miR-143-3p**	INO80D, CBFB, GLI3, GXYLT1, PTPN4, SECISBP2L, SMNDC1, VASH1, COL25A1, DIP2B, FNDC3B, FSCN1, HIPK2, HTR2C, KRT80, LRP12, NFATC1, PPM1E, SIX4, SLC38A2, SRSF11, TRAPPC8, TTPA, WWC3, ADD3, AKAP13, COL1A1, COL5A3, EPM2AIP1, ETV6, FRS2, GABARAPL1, KCNK10, MSI2, MYBL2, MYO6, PAN3, SH3PXD2A, SLC16A2, SLC25A15, SLC7A11, ST8SIA4, STRN3, TET1, UBXN2B, ZBTB44, ABHD14A, ABHD17B, ABL2, AFF1, AHCYL1, ARHGAP26, ASAP3, ATP10A, ATP6V1A, BRD2, CACHD1, CACNA1A, CHST10, CPD, CRELD1, EFS, EXOC6B, FADS6, FAM117A, FAR1, FBXO46, FGF1, FGF9, FNDC5, FOSL2, GDF10, GIGYF2, GOLM1, IGFBP5, ITGB8, ITM2B, KCND1, KIF3B, KRAS, LIMK1, LMO4, MAB21L1, MAP3K7, MAPK7, MEDAG, MOGS, NECAP1, NPR3, NUAK2, PC, PDIA6, PHF6, PSME4, RAB11FIP1, RBM24, SCAMP4, SLC30A8, SLC39A11, SLC4A8, SOBP, STAC, STOX2, SVIL, SYT3, THRB, TM2D2, TSHR, TUB, UBE2E3, VAPB, ZNF583
**hsa-mir-143-5p**	DDX3X, MIER3, SESN3, CASK, MAP3K2, SATB1, STRN, ARID4B, C5orf24, ESRRG, GLCE, LARP4, MMD, PTPN4, RAP2C, RC3H1, ASF1A, CDK14, FAM126A, FEZ2, HIPK2, KBTBD2, NELL2, PTHLH, RAPGEF6, RBPJ, RORA, SCN5A, SIX4, TOPORS, USP14, ZC3H12C, ZFP36L1, ZNF626, ZNF780B, ABCG2, ABHD10, ARL8B, DYRK1A, ELAVL4, EOGT, GPR85, HAS3, IKZF2, KDM2B, KIAA0319L, KPNA1, MAP3K11, MAP3K12, PRPF4B, RAD21, RHOQ, SKIDA1, SLC7A2, SPTBN1, TFDP1, VSNL1, ZNF138, ZNRF2, ADRB1, ANKRD11, BSPRY, BTBD2, BTN2A2, C12orf4, CADPS, CARD10, CDK12, CITED2, CORO2A, CRYBG3, DPH3, EMC2, ERP27, ESYT1, FAM111B, FAM81A, FMR1, G3BP2, GLRA2, HBS1L, HIF1A, HMGA2, ISL1, KCNJ2, LAMP1, LAPTM4A, LRCH1, MAP3K4, MAT2A, MKKS, MR1, NPLOC4, NPNT, NUAK1, PDS5A, PDX1, POLR3D, PPP2CA, RAB12, RALGPS1, RNF44, SACS, SGCZ, SNX3, STMN1, SUMF1, TAX1BP1, TBPL1, TEAD1, TEX2, TFB1M, THRA, THY1, TMEM260, TPPP, TTC38, UBE2Q2, VWC2L, WBP11, ZBTB18, ZNF460, ZNF85
**hsa-miR-200b-3p**	NUFIP2, PRKG1, BRWD3, FSTL1, MBNL3, PTAR1, SESN3, ATL2, ATXN1, CAB39, CAMSAP2, CNOT6, CTDSPL2, DGKH, EGLN1, ELL2, FOXN2, FRMD4B, GPM6A, NFIA, PPP1R9A, SLC4A7, STRN, TMEFF2, VEGFA, WIPF1, ZFX, AGFG1, ANK3, ARID4B, CCNJ, CDH20, CEP350, CEP97, CHN2, CLASP1, CYTH1, CYTH3, DACH1, DNMT3B, ESRRG, ETS1, FBXO30, GJC1, GLI3, GOLGA7, GPR158, GXYLT1, IMMP2L, KLF4, LRP1B, MAP4K3, MIB1, MMD, NOVA1, PDS5B, PHTF2, PIK3CA, PPP2R5E, RAP2C, REEP1, RPS6KA3, SDC2, SECISBP2L, SGCE, TBC1D12, TMX4, TRIM33, UBE2R2, ULK2, VASH1, ZC3H6, ZEB1, ACVR1C, ARL5A, ATAD2B, ATP11C, ATP6V0A2, BAP1, BCL11B, BNC2, C11orf87, C16orf72, CCNYL1, CHSY1, CKAP4, CLASP2, COPS8, CORO1C, CPED1, DNAJB14, DTNA, EFNB2, EIF2B5, ELAVL2, EVI5, FAT3, FBXO33, FBXW7, FERMT2, FEZ2, FNDC3B, FSCN1, GIT2, GNAQ, HIPK1, HIPK3, HMBOX1, HOOK1, JAZF1, KDELC1, KHDRBS1, KLF6, KRT80, LOX, MAP3K1, MAP4K4, MAPRE1, MBOAT2, MED13, MEX3D, NOTCH1, NOVA2, OCLN, OSBPL11, OTUD4, OXR1, PAG1, PDIK1L, PHACTR3, PI4K2B, PLXNC1, PPFIA1, PRDM1, PRKACB, PTHLH, PTPN14, PUM2, QKI, RBFOX2, RDH10, RND3, RNF2, RPS6KB1, SCAMP1, SCN5A, SGIP1, SLC39A14, SMARCD1, SPAG9, SUZ12, SYVN1, TBL1XR1, TMOD3, TRAPPC8, TSC22D2, UBE2W, USP25, USP6NL, WASF3, WWC3, XKR4, ZC3H4, ZEB2, ZMAT3, ZSWIM4, A1CF, ADAMTS3, ADIPOR2, AFF3, AMFR, AP1S2, APOO, ARHGAP20, ARHGAP6, ARIH1, ARL2BP, ASAP1, B3GNT2, BDP1, BICC1, CASR, CASZ1, CBL, CBX4, CCDC177, CCDC82, CDH11, CDK17, CDYL, CDYL2, CECR2, CEP41, CEP85L, CFL2, CHMP5, CHRDL1, CLIC4, CLIP1, CNEP1R1, CNKSR3, COL4A3BP, CRKL, CRTAP, CSMD3, CSNK1G3, DCBLD2, DENND5A, DENND5B, DESI1, DGKA, DIXDC1, DLC1, DNAJB5, DNAJB9, DNAJC3, DPY19L1, DUSP1, DZIP1, ELK3, ELK4, ELMOD2, EPS8, ERG, ERRFI1, FAM118B, FAM8A1, FARP1, FIGN, FLI1, FLII, FN1, FOXF1, FOXG1, FRMD6, GABBR2, GATA4, GLCCI1, GOLGA1, GPATCH8, HDHD2, HMGB3, HNRNPD, HS2ST1, HS3ST1, HS3ST3A1, HSPA13, IER5, IGF2R, INTS8, JAKMIP2, JKAMP, JUN, KANK2, KCND2, KDR, KIAA0355, LBR, LRP1, LRRC8A, MAP2, MARCH6, MARCKS, MATR3, MCFD2, MFAP5, MGAT2, MIEF1, MPRIP, MSL2, MSN, MTF2, MTSS1L, MYB, MYZAP, NAB1, NANOS1, NBR1, NCOA2, NCS1, NEDD1, NOG, NPC1, NR3C1, NR5A2, NRBP1, NRG1, NTF3, NUDT4, NYAP1, OSTM1, PCDH19, PCMTD1, PHF21B, PI4KB, PIKFYVE, PITPNM3, PKD1, PKIA, PLCL1, PMAIP1, POLK, PPM1F, PPP1R10, PPP1R18, PPP1R9B, PPP4R2, PRDM16, PSAT1, PSIP1, PTPN12, PTPN21, RAB11FIP2, RAB21, RANBP10, RANBP9, RAP1B, RASA2, RASSF8, RBFOX3, RECK, RGL1, RIMS2, RIPK2, ROCK2, RTF1, RUSC2, S100PBP, SBSPON, SCD, SCN8A, SCRT2, SEC23A, SEMA3F, SEMA6D, SERINC1, SERPINI1, SESN1, SFXN1, SIX1, SLC14A1, SLC1A2, SLC24A4, SLC35E2B, SLC6A1, SLC6A11, SLIT2, SLITRK1, SLK, SOX2, SRGAP1, SULF1, SYDE1, SYNJ1, TAF12, TBK1, TCAIM, TFAP2A, THAP1, TIMP2, TLN1, TLN2, TMEM136, TMEM164, TMEM17, TOB1, TRHDE, TSC22D1, TUBB, UHRF1BP1, USP27X, VASH2, VAT1L, VLDLR, WASF1, WDR82, XKR8, YPEL2, YWHAB, YWHAG, ZBTB38, ZCCHC24, ZFAND6, ZFPM2, ZNF131, ZNF217, ZNF532, ZNF711, ZYG11B

**hsa-miR-200b-5p**	*MIER3, AHR, NFIA, KLF12, AMMECR1, ATP6V0A2, COPS8, EID1, EPHA5, HCFC2, PPM1E, SGK1, ZC3H12C, ARL13B, BBX, CRLF3, EPM2AIP1, KCTD12, KDM2B, KMT2E, LIMCH1, MDFIC, NAMPT, NDFIP1, NDFIP2, NSG2, ORC2, PAPOLG, SHPRH, SP3, UBR3, ZNF138, ZNF254, AGPAT5, ALX4, ARC, ASNSD1, ATAD2, ATG10, ATL3, BTBD1, C11orf53, C18orf54, CHD7, CRIM1, DNAJC27, EIF1AX, EIF4ENIF1, ETFDH, FGF13, FOXC1, FOXD1, GOLGA7B, GRHL1, HAUS6, HIVEP3, HOXA10, HPDL, IFT52, INSIG2, KIAA1217, KLF7, KLHL5, LMTK2, LRRC57, MOBP, MYBL1, NCF2, NR4A1, PCDHB6, PKP2, PPRC1, RAB11B, RAB1A, RANBP3, RUFY2, SCOC, SEMA3E, SERINC3, SH3RF1, SLC22A15, SLC25A40, SNX4, SULT1C2, THAP5, TIAM2, TMEM39A, TXNDC17, TXNRD3, USP53, ZNF208, ZNF367, ZNF440, ZNF493, ZNF675, ZNF763, ZNF770, ZNF91, ZNF99*
**hsa-miR-203a-3p**	*NUFIP2, PRKG1, ZBTB20, MBNL3, AHR, ANTXR2, CAB39, CASK, DGKH, ELL2, MAP3K2, RFX7, CADM2, EIF5A2, GUCY1A2, GXYLT1, HOOK3, MBNL2, MYEF2, OSBPL8, PDE4D, PIK3CA, PTP4A1, ROBO2, SLC12A2, THSD7A, TNPO1, YTHDF3, AFF4, ARHGAP42, CDH10, COPS7B, CREBRF, DDX6, DENND6A, DIP2B, DNAJB14, EIF4E, FAT3, GPR180, KHDRBS1, MAP3K1, PRICKLE2, RAB10, RTKN2, SGMS2, SLC4A4, SLC7A14, TCF4, TRPV3, ABCE1, ACVR2A, ADAMTS17, ADK, BBX, CLOCK, COL4A4, CSRNP2, DCP2, DLX5, EGR3, ETV1, FAM126B, GABARAPL1, GABRA1, KIAA1211, MED14, MSI2, NEDD4L, NLK, NUDT21, PAQR3, PDAP1, PEX5L, PHLDA1, PRKCB, PRKCI, ROBO1, SESTD1, SGTB, SLC39A9, SPARC, VAPA, VSNL1, ZMIZ1, ZNF148, ZNF608, ACO2, ADAMTS5, ANKRD52, ATF2, ATG14, BCL7A, C11orf91, CNNM3, COL17A1, CSN2, DGKB, DLG5, DR1, DUSP5, E2F3, ETS2, FAM84A, FOXK1, FUBP3, GPATCH1, GRHL3, HNRNPUL2, ID4, IL24, INSIG1, KAT6A, KIF2A, KRT1, KRT35, KRT85, LNX2, MAPK8, MBNL1, MCTP1, MCTS1, MICAL2, NAA30, NEMF, NFIL3, NR1D2, NSG1, OPA1, PAPSS2, PDGFD, PHF12, PLD1, PPP1R12A, PRPS2, PTPN3, RAB27B, RAPGEF1, RASAL2, RBM47, SCGB2A1, SEC62, SEMA3A, SEMA5A, SMC5, SNAI2, SNTB2, SP4, SRA1, SRC, TADA2B, TARDBP, TEDDM1, TFDP2, TMEM100, TMEM69, TNFSF15, TTC39A, UBR1, UPF2, USP8, WDR37, XRN2, YAF2, ZNF197, ZNF281*
**hsa-mir-203a-5p**	*WIPF1, MAP4K3, ZC3H6, BTG2, CREBRF, CSGALNACT2, DIP2B, DTNA, KBTBD2, PPFIA2, PTPN14, TSC22D2, ABHD3, APAF1, C1QTNF6, CNOT6L, ELOVL5, EPC1, FRAS1, JAG1, LASP1, NT5E, PHLDA1, UBE2D3, ZNRF3, AARS, ABCC4, ABHD12B, ANKRD44, ARMC1, CCDC113, CCER1, CCL2, CCNG1, CDH6, CDKL1, CDKL2, COLQ, FAM102A, GRASP, IGF1R, KLF11, KRIT1, MEX3C, MICAL3, NBPF1, PFKFB3, PPTC7, RNF11, SH3BGRL, TBP, TUBD1, ZBTB4, ZNF800*
**hsa-mir-219b**	*FOXN2, SATB1, ESRRG, PEAK1, PHTF2, ZFHX3, ALCAM, CSGALNACT2, NAV1, RSRC1, UNC5C, YOD1, ABHD10, ECHDC1, EFR3A, EOMES, KLF5, NEUROD1, PAQR3, PLEC, PRKAR2B, SLC6A14, SOX21, TENM1, UBN1, ZIC3, ZNF254, ARHGAP28, CAMK1D, CLEC4E, DPP10, EPHA4, EPHB1, FMNL2, FNBP4, GEMIN2, GJB7, GRID2, IBTK, IPO5, IQGAP2, KRTAP6-3, MPZ, OGFOD1, PACRG, RAB6A, SAMD13, SAMD9, SOD3, STAR, TCP1, TNFSF13B, ZNF561*
**hsa-mir-29c**	*HEBP2, RPA3, TCEA3, TMEM98*
**hsa-miR-29c-3p**	*PRKG1, ZBTB20, BRWD3, DDX3X, FSTL1, ANTXR2, CAMSAP2, DGKH, NFIA, RFX7, VEGFA, ZFX, ZNF704, CCNJ, CCNT2, CEP97, COL19A1, DICER1, DNMT3B, GAB1, KLF4, PGAP1, RNF138, STX16, TMEM65, USP37, VASH1, ZMYM2, ANKRD13C, ATAD2B, BTG2, C16orf72, CCNYL1, CCSER2, CHSY1, CMPK1, CNR1, COL25A1, COL4A1, CSGALNACT2, DAAM1, DCAF7, DENND6A, EML5, FAM168B, FBXW7, FERMT2, GNB4, GPATCH2, IL1RAP, IREB2, JAZF1, KDELC1, LOX, MAP4K4, MAPRE1, MYCN, N4BP2L1, NAV1, NFAT5, NPAS3, OTUD4, PAIP2, PDIK1L, PPM1E, RNF19A, SGK1, SGMS2, SH3GLB1, SP1, TCF4, TMOD3, USP34, USP6NL, XKR4, ZBTB10, ZFP36L1, ABCE1, ADAMTS17, AKAP13, ANKRD27, ARPP19, BACH2, BAK1, BMF, C1QTNF6, CAMK4, CDC42BPA, CDK6, COL11A1, COL1A1, COL4A4, COL5A3, CPEB3, CSRNP2, DCUN1D4, DCX, DLG2, DTWD2, DTX4, ELMSAN1, EOMES, EPC1, ETV6, FGD4, FRAS1, GRIP1, HAS3, HDAC4, KCTD5, LASP1, MAPK10, MAPRE2, MBTD1, MCL1, MYBL2, NOTCH2, NREP, PAN2, PAPOLG, PCDHA1, PCDHA10, PCDHA11, PCDHA12, PCDHA13, PCDHA2, PCDHA3, PCDHA4, PCDHA5, PCDHA6, PCDHA7, PCDHA8, PCDHAC1, PCDHAC2, PMP22, PRR14L, PRR3, PRRC2C, PTBP3, PTPRK, RAB30, RAP1A, RBAK, REPS2, RERE, REST, ROBO1, SESTD1, SH3BP5L, SH3PXD2A, SHPRH, SIRT1, SLC6A14, SMIM17, SMPD3, SPARC, STX17, TDG, TET1, TFAP2C, TLL1, TMEM236, TNFAIP3, TNRC18, ZMIZ1, ADAMTS10, ADAMTS2, ADAMTS7, ADAMTS9, AKAP5, AKT3, AMER1, AMMECR1L, ANKRD13B, ARHGEF10, ARVCF, ASAP2, ASXL3, ATP1B4, ATP2B4, ATRN, B3GNT5, BCORL1, BLMH, C5orf15, CAV2, CBX6, CCSAP, CD276, CDC42, CLDN1, CLEC2L, COL15A1, COL1A2, COL22A1, COL27A1, COL2A1, COL3A1, COL4A2, COL4A5, COL4A6, COL5A1, COL5A2, COL6A3, COL7A1, COL9A1, CPS1, CPSF7, CREB5, CRISPLD1, CUEDC1, DAAM2, DGKD, DIO2, DNMT3A, DOLPP1, DOT1L, DPYSL3, DPYSL5, DYNLT1, EFNA5, EIF3J, EIF4E2, ELF2, ELN, ELOVL4, EML4, EML6, ENHO, ENPP2, ERCC6, ERLIN2, ERP44, FAM13B, FAM167A, FAM57B, FBN1, FBXW9, FEM1B, FOXJ2, FRAT2, FREM2, GID8, GNG12, GPCPD1, GPR37, GPX7, GXYLT2, HAPLN3, HBEGF, HBP1, HIF3A, HMCN1, HRK, HS3ST3B1, ICOS, IFFO1, IFI30, IGF1, INA, ING2, IQCJ-SCHIP1, ISG20L2, JARID2, JMY, KCTD20, KDM4B, KDM5B, KIAA0895, KIAA1549, KIF26A, KIF26B, KLHL28, KNOP1, LAMA2, LAMC1, LDLRAD3, LIF, LIN7A, LOXL2, LOXL4, LPL, LSM11, LYSMD1, MAPKBP1, MARCH1, MED12L, METAP2, MEX3B, MFAP3, MGA, MLXIP, MOB1A, MORF4L1, MTMR4, MXD1, NAPB, NASP, NAV2, NAV3, NCKAP5, NKIRAS2, NKRF, NLRX1, NSD1, NUP160, OSTC, PALM, PARG, PCDHA9, PCGF3, PCSK5, PGAP2, PI15, PIK3R1, PLP1, PPIC, PPP1R13B, PPP1R15B, PRKAB2, PRKRA, PRPF40A,*

	PTEN, PXDN, RAP1GDS1, RAPGEFL1, RARB, REL, REV3L, RHOBTB1, RLF, RMND5A, RNF39, SAMD4A, SCML2, SENP1, SERINC5, SERPINH1, SETDB1, SETDB2, SH3PXD2B, SHROOM2, SIDT1, SIDT2, SLC16A14, SLC30A3, SLC5A8, SLC7A6, SMS, SMTNL2, SNX24, SS18L1, STMN2, STRN4, TAF11, TAF5, TET2, TET3, TFEB, TFEC, TIMM8B, TMEM169, TMEM178B, TMEM183A, TMTC3, TNFRSF1A, TPK1, TRAF3, TRAF4, TRIB2, TRIM63, TTC9, TUBB2A, UBFD1, VPS37C, WBP1L, WDFY1, XKR6, XKR7, YBX3, ZBTB34, ZBTB5, ZDHHC21, ZFP91, ZHX3, ZNF282, ZNF469, ZNF512B
**hsa-miR-375-3p**	*ZBTB20, ATXN7, DIP2C, POU3F1, QKI, RBPJ, SPAG9, ELAVL4, KLF5, SLC16A2, ZFP36L2, CREBZF, HNF1B, NFIX, PLEKHA3, SOCS5, TSC1, UBE3A*
**hsa-miR-429**	*NUFIP2, PRKG1, ZBTB20, BRWD3, FSTL1, MBNL3, PTAR1, SESN3, ATL2, ATXN1, CAB39, CAMSAP2, CNOT6, CTDSPL2, DGKH, EGLN1, ELL2, FOXN2, FRMD4B, GPM6A, NFIA, PPP1R9A, SLC4A7, STRN, TMEFF2, VEGFA, WIPF1, ZFX, AGFG1, ANK3, ARID4B, CCNJ, CDH20, CEP350, CEP97, CHN2, CLASP1, CYTH1, CYTH3, DACH1, DNMT3B, ESRRG, ETS1, FBXO30, GJC1, GLI3, GOLGA7, GPR158, GXYLT1, IMMP2L, KLF4, LRP1B, MAP4K3, MIB1, MMD, NOVA1, PDS5B, PHTF2, PIK3CA, PPP2R5E, RAP2C, REEP1, RPS6KA3, SDC2, SECISBP2L, SGCE, TBC1D12, TMX4, TRIM33, UBE2R2, ULK2, VASH1, ZC3H6, ZEB1, ACVR1C, ARL5A, ATAD2B, ATP11C, ATP6V0A2, BAP1, BCL11B, BNC2, C11orf87, C16orf72, CCNYL1, CHSY1, CKAP4, CLASP2, COPS8, CORO1C, CPED1, DNAJB14, DTNA, EFNB2, EIF2B5, ELAVL2, EVI5, FAT3, FBXO33, FBXW7, FERMT2, FEZ2, FNDC3B, FSCN1, GIT2, GNAQ, HIPK1, HIPK3, HMBOX1, HOOK1, JAZF1, KDELC1, KHDRBS1, KLF6, KRT80, LOX, MAP3K1, MAP4K4, MAPRE1, MBOAT2, MED13, MEX3D, NOTCH1, NOVA2, OCLN, OSBPL11, OTUD4, OXR1, PAG1, PDIK1L, PHACTR3, PI4K2B, PLXNC1, PPFIA1, PRDM1, PRKACB, PTHLH, PTPN14, PUM2, QKI, RBFOX2, RDH10, RND3, RNF2, RPS6KB1, SCAMP1, SCN5A, SGIP1, SLC39A14, SMARCD1, SPAG9, SUZ12, SYVN1, TBL1XR1, TMOD3, TRAPPC8, TSC22D2, UBE2W, USP25, USP6NL, WASF3, WWC3, XKR4, ZC3H4, ZEB2, ZMAT3, ZSWIM4, A1CF, ADAMTS3, ADIPOR2, AFF3, AMFR, AP1S2, APOO, ARHGAP20, ARHGAP6, ARIH1, ARL2BP, ASAP1, B3GNT2, BDP1, BICC1, CASR, CASZ1, CBL, CBX4, CCDC177, CCDC82, CDH11, CDK17, CDYL, CDYL2, CECR2, CEP41, CEP85L, CFL2, CHMP5, CHRDL1, CLIC4, CLIP1, CNEP1R1, CNKSR3, COL4A3BP, CRKL, CRTAP, CSMD3, CSNK1G3, DCBLD2, DENND5A, DENND5B, DESI1, DGKA, DIXDC1, DLC1, DNAJB5, DNAJB9, DNAJC3, DPY19L1, DUSP1, DZIP1, ELK3, ELK4, ELMOD2, EPS8, ERG, ERRFI1, FAM118B, FAM8A1, FARP1, FIGN, FLI1, FLII, FN1, FOXF1, FOXG1, FRMD6, GABBR2, GATA4, GLCCI1, GOLGA1, GPATCH8, HDHD2, HMGB3, HNRNPD, HS2ST1, HS3ST1, HS3ST3A1, HSPA13, IER5, IGF2R, INTS8, JAKMIP2, JKAMP, JUN, KANK2, KCND2, KDR, KIAA0355, LBR, LCA5, LRP1, LRRC8A, MAP2, MARCH6, MARCKS, MATR3, MCFD2, MFAP5, MGAT2, MIEF1, MPRIP, MSL2, MSN, MTF2, MTSS1L, MYB, MYZAP, NAB1, NANOS1, NBR1, NCOA2, NCS1, NEDD1, NOG, NPC1, NR3C1, NR5A2, NRBP1, NRG1, NTF3, NUDT4, NYAP1, OSTM1, PCDH19, PCMTD1, PHF21B, PI4KB, PIKFYVE, PITPNM3, PKD1, PKIA, PLCL1, PMAIP1, POLK, PPM1F, PPP1R10, PPP1R18, PPP1R9B, PPP4R2, PRDM16, PSAT1, PSIP1, PTPN12, PTPN21, RAB11FIP2, RAB21, RANBP10, RANBP9, RAP1B, RASA2, RASSF8, RBFOX3, RECK, RGL1, RIMS2, RIPK2, ROCK2, RSPRY1, RTF1, RUSC2, S100PBP, SBSPON, SCD, SCN8A, SCRT2, SEC23A, SEMA3F, SEMA6D, SERINC1, SERPINI1, SESN1, SFXN1, SIX1, SLC14A1, SLC1A2, SLC24A4, SLC35E2B, SLC6A1, SLC6A11, SLIT2, SLITRK1, SLK, SOX2, SRGAP1, SULF1, SYDE1, SYNJ1, TAF12, TBK1, TCAIM, TFAP2A, THAP1, TIMP2, TLN1, TLN2, TMEM136, TMEM164, TMEM17, TOB1, TRHDE, TSC22D1, TUBB, UHRF1BP1, USP27X, VASH2, VAT1L, VLDLR, WASF1, WDR82, XKR8, YPEL2, YWHAB, YWHAG, ZBTB38, ZCCHC24, ZFAND6, ZFPM2, ZNF131, ZNF217, ZNF532, ZNF711, ZYG11B,* LARP1B, NDN, PPP1CB
**hsa-mir-7-1**	*NUFIP2, PRKG1, ZBTB20, BRWD3, DDX3X, FSTL1, MBNL3, MIER3, PTAR1, SESN3, AHR, ANTXR2, ATL2, CAB39, CAMSAP2, CASK, CNOT6, CTDSPL2, EGLN1, ELL2, FOXN2, FRMD4B, GPM6A, INO80D, MAP3K2, PPP1R9A, RFX7, SATB1, SLC4A7, TMEFF2, VEGFA, WIPF1, ZFX, AGFG1, ANK3, ATXN7, BNIP2, C5orf24, CACNB4, CADM2, CBFB, CCNT2, CDH20, CEP350, CHN2, CLASP1, COL19A1, CREB1, CYTH1, DICER1, DPYSL2, EIF5A2, FBXO28, FBXO30, GAB1, GLCE, GMFB, GOLGA7, GPR158, GUCY1A2, HOOK3, IMMP2L, KLF12, LARP4, LRP1B, MBNL2, MIB1, MYEF2, NOVA1, NRXN1, OSBPL8, PDE4D, PDS5B, PEAK1, PGAP1, PPP2R5E, PTP4A1, PTPN4, RBMS3, RC3H1, REEP1, RNF138, ROBO2, RPS6KA3, SDC2, SEL1L, SGCE, SLC12A2, SMNDC1, STX16, TAF9B, TAOK1, TBC1D12, THSD7A, TMEM65, TMX4, TNPO1, USP37, YTHDF3, ZEB1, ZFHX3, ZMYM2, ABCB1, ABHD13, AEBP2, AFF4, ALCAM, ALDH1L2, AMMECR1, ANKRD13C, ARHGAP12, ARHGAP42, ASF1A, ATAD1, BAZ1A, CBX5, CCDC50, CCDC88A, CCSER1, CCSER2, CDC42SE2, CDC73, CDH10, CDK14, CMPK1, CNR1, CNTLN, COL4A1, COPS7B, CPEB2, CTBP2, DCAF7, DDX6, DIP2C, EEA1, EID1, EIF4E, EML5, EPHA5, ESM1, ETNK1, EXD1, FAM126A, FAM19A2, FAT4, FBXO9, FGF14, FGFR1OP2, FGL2, FXR1, GNB4, GPATCH2, GPC4, GPR137B, GPR180, HAPLN1, HCFC2, HCN1, HDAC9, HMGCLL1, HTR2C, IL1RAP, IPMK, IREB2, ITGB1, KCNK2, KDM4A, KDM4C, KIAA1841, KLF8, KLHL15, KLHL24, LCLAT1, LRP12, LRRC58, MAGT1, MAPK1IP1L, MAPK9, MGAT4A, MYCN, MYNN, N4BP2L1, NEK7, NELL2, NETO2, NFATC1, NFE2L2, NPAS3, NUCKS1, ONECUT2, PAIP2, PARP8, PDLIM5, PEX3, PFDN4, PHC3, PIWIL1, POU3F1, PPFIA2, PPP3CA, PRICKLE2, PSD3, PTGFRN, RAB10, RALGPS2, RAPGEF6, RBM7, REEP3, RNF19A, RSRC1, SCEL, SEC24A, SETD2, SKIL, SLAIN1, SLC17A6, SLC19A2, SLC25A16, SLC35A3, SLC7A14, SMIM14, SNCA, SRSF11, SSBP3, STAM2, STXBP5, SYT14, TAL1, TM9SF2, TM9SF3, TMEM181, TMEM64, TMX3, TOPORS, TP53INP1, TRAF6, TRPV3, TTC30B, UNC5C, USP14, USP34, VGLL3, VPS26A, YOD1, ZBTB10, ZFHX4, ZNF626, ZNF780B, ACTA1, ACTR10, ACTR2, ACTR8, ACYP2, ADNP2, AGMO, AKAP6, AMER2, ANGPTL3, ANKRD46, APC, AQP4, AQR, ARHGAP44, ARHGAP5, ARHGEF12, ARMCX1, ART3, ASB5, ASB7, ASB9, ASTN1,*

	ATP1A2, ATP8A1, ATRX, AZIN1, BCAP29, BCCIP, BCL2L1, BCLAF1, BDNF, BEND4, BHLHE22, BMPR1B, BTBD10, BTF3L4, BTN3A1, BTN3A2, BZW1, C10orf88, C12orf29, C12orf56, C18orf63, C2orf69, C5orf47, CACNA1C, CALCR, CAMTA2, CAPRIN1, CBLL1, CBLN4, CCDC126, CCNG2, CCNY, CD2AP, CDC23, CDC27, CDH19, CDH8, CDK1, CDKN2B, CEP170, CFHR2, CHM, CHRNA6, CLCA4, CLDN11, CNOT7, COPB1, CPE, CPEB4, CRABP2, CREBBP, CSRNP1, CTLA4, CTNND2, CXADR, CXCL2, CXorf56, CYP26B1, DACT1, DBI, DCAF12L1, DCAF13, DCUN1D3, DCUN1D5, DEK, DLG2, DLX2, DMRT1, DMXL2, DNAH14, DOK5, DPY19L3, EBF2, EDEM3, EDIL3, EEF1E1, EIF3A, ELF1, ENO2, EPB41L3, EPS15, ERI1, EXOC5, EXOC6, FAM13C, FAM169A, FAM184A, FAM19A4, FAM91A1, FAN1, FAXC, FBXL17, FBXL3, FBXO45, FER, FGF2, FGF7, FMN1, FNIP1, FPR3, FRMD3, FSD1L, FZD8, G3BP1, GABPB1, GABRA4, GDF6, GGPS1, GJA1, GMEB1, GOLGA6L9, GOPC, GPR17, GPR19, GPR22, GRIA1, GTF2A1, GTF2H3, GULP1, GZF1, HDAC2, HERC2, HINT3, HIVEP2, HNRNPA3, HNRNPR, HOXA5, HP1BP3, HSD11B1, HSP90AA1, HSPA5, HTATSF1, IDH1, IFT57, IL13RA1, IMPACT, ITGA4, ITGB6, ITIH6, ITPR2, KAT6B, KCNC2, KCNH5, KCTD9, KHDRBS2, KHDRBS3, KIAA0232, KIF5C, KLF3, KLHL2, KSR2, LMAN1, LRBA, LRRCC1, LRRTM2, MACROD2, MANEA, MAP3K5, MAP7, MAT1A, MB21D2, MBLAC2, MCC, MCEE, MCOLN2, MCU, MECOM, MED12, MED6, MEF2D, MFAP4, MIPOL1, MLF1, MORF4L2, MPC1, MPL, MSL3, MSX1, MTDH, MTSS1, MUC13, MVB12B, MYSM1, NAA15, NANP, NAP1L1, NAP1L2, NCAPG2, NETO1, NEUROD6, NHSL1, NIPBL, NKAIN2, NMT2, NOD1, NRXN3, NTN4, NUDT5, NXPH2, OLA1, OPCML, OR2L13, OTUD6B, OXNAD1, PABPC5, PANK3, PAX3, PBRM1, PCDH11X, PCDH18, PCDH9, PDCD1LG2, PDE3B, PDE5A, PDGFA, PELI1, PER3, PHF1, PHF19, PHF20L1, PHLPP1, PIAS2, PICALM, PIGM, PIK3CB, PJA2, PLD5, PLEKHG4, PLXNA4, PNLIPRP3, PNRC1, POLDIP3, POLR3A, POLR3G, PPARGC1A, PPP3R1, PRICKLE1, PRKAR1A, PRR23B, PSMA5, PTCD2, PTGS2, PTPDC1, PTPRA, PTPRF, RAB18, RAB2A, RAB3C, RAB3IP, RASSF6, RBM12, RBM46, RCSD1, RIF1, RIMBP2, RIMKLB, RMI1, RNF111, RNF139, RORB, RPE, RPGRIP1L, RRM2, RSPO3, RWDD3, SATB2, SCAF8, SCG2, SCYL3, SEC14L1, SEC23IP, SENP6, SENP7, SF3B1, SH3BGRL2, SIAH1, SKAP2, SLC10A7, SLC12A5, SLC13A1, SLC25A24, SLC30A5, SLC39A8, SLC5A7, SLC6A2, SLC7A7, SLC9C1, SLITRK4, SMARCA2, SNTG1, SOS1, SPCS2, SPIN1, SPIN4, SPP1, SPTSSA, SREK1IP1, SYNPO2, TASP1, TBC1D32, TEAD3, TFAP4, TGFBR3, THAP2, THBS2, THEMIS, THRAP3, TIMM21, TMED2, TMEM132C, TMEM170B, TMEM220, TMEM245, TMEM30B, TMEM33, TMEM41B, TMEM47, TMOD2, TMPRSS11A, TNKS, TOR1A, TOR1AIP1, TOR1AIP2, TPD52L1, TRAF7, TRIM2, TRIM32, TRPA1, TRPC1, TRPM6, TTC27, TTYH3, TXLNB, TXNRD1, U2SURP, UBE2E1, UBE2V2, UFM1, UHMK1, UHRF1BP1L, UPRT, USP51, VGLL4, VPS41, WDFY3, WDR48, WDR76, WFS1, ZC3HAV1L, ZCRB1, ZFP42, ZFR, ZFYVE16, ZIC4, ZMYM5, ZMYND11, ZNF23, ZNF264, ZNF273, ZNF280B, ZNF519, ZNF638, ZNHIT6, **NUTF2**
**hsa-mir-7-2**	NUFIP2, PRKG1, ZBTB20, BRWD3, DDX3X, FSTL1, MBNL3, MIER3, PTAR1, SESN3, AHR, ANTXR2, ATL2, CAB39, CAMSAP2, CASK, CNOT6, CTDSPL2, EGLN1, ELL2, FOXN2, FRMD4B, GPM6A, INO80D, MAP3K2, PPP1R9A, RFX7, SATB1, SLC4A7, TMEFF2, VEGFA, WIPF1, ZFX, AGFG1, ANK3, ATXN7, BNIP2, C5orf24, CACNB4, CADM2, CBFB, CCNT2, CDH20, CEP350, CHN2, CLASP1, COL19A1, CREB1, CYTH1, DICER1, DPYSL2, EIF5A2, FBXO28, FBXO30, GAB1, GLCE, GMFB, GOLGA7, GPR158, GUCY1A2, HOOK3, IMMP2L, KLF12, LARP4, LRP1B, MBNL2, MIB1, MYEF2, NOVA1, NRXN1, OSBPL8, PDE4D, PDS5B, PEAK1, PGAP1, PPP2R5E, PTP4A1, PTPN4, RBMS3, RC3H1, REEP1, RNF138, ROBO2, RPS6KA3, SDC2, SEL1L, SGCE, SLC12A2, SMNDC1, STX16, TAF9B, TAOK1, TBC1D12, THSD7A, TMEM65, TMX4, TNPO1, USP37, YTHDF3, ZEB1, ZFHX3, ZMYM2, ABCB1, ABHD13, AEBP2, AFF4, ALCAM, ALDH1L2, AMMECR1, ANKRD13C, ARHGAP12, ARHGAP42, ASF1A, ATAD1, BAZ1A, CBX5, CCDC50, CCDC88A, CCSER1, CCSER2, CDC42SE2, CDC73, CDH10, CDK14, CMPK1, CNR1, CNTLN, COL4A1, COPS7B, CPEB2, CTBP2, DCAF7, DDX6, DIP2C, EEA1, EID1, EIF4E, EML5, EPHA5, ESM1, ETNK1, EXD1, FAM126A, FAM19A2, FAT4, FBXO9, FGF14, FGFR1OP2, FGL2, FXR1, GNB4, GPATCH2, GPC4, GPR137B, GPR180, HAPLN1, HCFC2, HCN1, HDAC9, HMGCLL1, HTR2C, IL1RAP, IPMK, IREB2, ITGB1, KCNK2, KDM4A, KDM4C, KIAA1841, KLF8, KLHL15, KLHL24, LCLAT1, LRP12, LRRC58, MAGT1, MAPK1IP1L, MAPK9, MGAT4A, MYCN, MYNN, N4BP2L1, NEK7, NELL2, NETO2, NFATC1, NFE2L2, NPAS3, NUCKS1, ONECUT2, PAIP2, PARP8, PDLIM5, PEX3, PFDN4, PHC3, PIWIL1, POU3F1, PPFIA2, PPP3CA, PRICKLE2, PSD3, PTGFRN, RAB10, RALGPS2, RAPGEF6, RBM7, REEP3, RNF19A, RSRC1, SCEL, SEC24A, SETD2, SKIL, SLAIN1, SLC17A6, SLC19A2, SLC25A16, SLC35A3, SLC7A14, SMIM14, SNCA, SRSF11, SSBP3, STAM2, STXBP5, SYT14, TAL1, TM9SF2, TM9SF3, TMEM181, TMEM64, TMX3, TOPORS, TP53INP1, TRAF6, TRPV3, TTC30B, UNC5C, USP14, USP34, VGLL3, VPS26A, YOD1, ZBTB10, ZFHX4, ZNF626, ZNF780B, ACTA1, ACTR10, ACTR2, ACTR8, ACYP2, ADNP2, AGMO, AKAP6, AMER2, ANGPTL3, ANKRD46, APC, AQP4, AQR, ARHGAP44, ARHGAP5, ARHGEF12, ARMCX1, ART3, ASB5, ASB7, ASB9, ASTN1, ATP1A2, ATP8A1, ATRX, AZIN1, BCAP29, BCCIP, BCL2L1, BCLAF1, BDNF, BEND4, BHLHE22, BMPR1B, BTBD10, BTF3L4, BTN3A1, BTN3A2, BZW1, C10orf88, C12orf29, C12orf56, C18orf63, C2orf69, C5orf47, CACNA1C, CALCR, CAMTA2, CAPRIN1, CBLL1, CBLN4, CCDC126, CCNG2, CCNY, CD2AP, CDC23, CDC27, CDH19, CDH8, CDK1, CDKN2B, CEP170, CFHR2, CHM, CHRNA6, CLCA4, CLDN11, CNOT7, COPB1, CPE, CPEB4, CRABP2, CREBBP, CSRNP1, CTLA4, CTNND2, CXADR, CXCL2, CXorf56, CYP26B1, DACT1, DBI, DCAF12L1, DCAF13, DCUN1D3, DCUN1D5, DEK, DLX2, DMRT1, DMXL2, DNAH14, DOK5, DPY19L3, EBF2, EDEM3, EDIL3, EEF1E1, EIF3A, ELF1, ENO2, EPB41L3, EPS15, ERI1, EXOC5, EXOC6, FAM13C, FAM169A, FAM184A, FAM19A4, FAM91A1, FAN1, FAXC, FBXL17, FBXL3, FBXO45, FER, FGF2, FGF7, FMN1, FNIP1, FPR3, FRMD3, FSD1L, FZD8, G3BP1, GABPB1, GABRA4, GDF6, GGPS1, GJA1, GMEB1, GOLGA6L9, GOPC, GPR17, GPR19, GPR22, GRIA1, GTF2A1, GTF2H3, GULP1, GZF1, HDAC2, HERC2, HINT3, HIVEP2, HNRNPA3, HNRNPR, HOXA5, HP1BP3, HSD11B1, HSP90AA1,

	HSPA5, HTATSF1, IDH1, IFT57, IL13RA1, IMPACT, ITGA4, ITGB6, ITIH6, ITPR2, KAT6B, KCNC2, KCNH5, KCTD9, KHDRBS2, KHDRBS3, KIAA0232, KIF5C, KLF3, KLHL2, KSR2, LMAN1, LRBA, LRRCC1, LRRTM2, MACROD2, MANEA, MAP3K5, MAP7, MAT1A, MB21D2, MBLAC2, MCC, MCEE, MCOLN2, MCU, MECOM, MED12, MED6, MEF2D, MFAP4, MIPOL1, MLF1, MORF4L2, MPC1, MPL, MSL3, MSX1, MTDH, MTSS1, MUC13, MVB12B, MYSM1, NAA15, NANP, NAP1L1, NAP1L2, NCAPG2, NETO1, NEUROD6, NHSL1, NIPBL, NKAIN2, NMT2, NOD1, NRXN3, NTN4, NUDT5, NXPH2, OLA1, OPCML, OR2L13, OTUD6B, OXNAD1, PABPC5, PANK3, PAX3, PBRM1, PCDH11X, PCDH18, PCDH9, PDCD1LG2, PDE3B, PDE5A, PDGFA, PELI1, PER3, PHF1, PHF19, PHF20L1, PHLPP1, PIAS2, PICALM, PIGM, PIK3CB, PJA2, PLD5, PLEKHG4, PLXNA4, PNLIPRP3, PNRC1, POLDIP3, POLR3A, POLR3G, PPARGC1A, PPP3R1, PRICKLE1, PRKAR1A, PRR23B, PSMA5, PTCD2, PTGS2, PTPDC1, PTPRA, PTPRF, RAB18, RAB2A, RAB3C, RAB3IP, RASSF6, RBM12, RBM46, RCSD1, RIF1, RIMBP2, RIMKLB, RMI1, RNF111, RNF139, RORB, RPE, RPGRIP1L, RRM2, RSPO3, RWDD3, SATB2, SCAF8, SCG2, SCYL3, SEC14L1, SEC23IP, SENP6, SENP7, SF3B1, SH3BGRL2, SIAH1, SKAP2, SLC10A7, SLC12A5, SLC13A1, SLC25A24, SLC30A5, SLC39A8, SLC5A7, SLC6A2, SLC7A7, SLC9C1, SLITRK4, SMARCA2, SNTG1, SOS1, SPCS2, SPIN1, SPIN4, SPP1, SPTSSA, SREK1IP1, SYNPO2, TASP1, TBC1D32, TEAD3, TFAP4, TGFBR3, THAP2, THBS2, THEMIS, THRAP3, TIMM21, TMED2, TMEM132C, TMEM170B, TMEM220, TMEM245, TMEM30B, TMEM33, TMEM41B, TMEM47, TMOD2, TMPRSS11A, TNKS, TOR1A, TOR1AIP1, TOR1AIP2, TPD52L1, TRAF7, TRIM2, TRIM32, TRPA1, TRPC1, TRPM6, TTC27, TTYH3, TXLNB, TXNRD1, U2SURP, UBE2E1, UBE2V2, UFM1, UHMK1, UHRF1BP1L, UPRT, USP51, VGLL4, VPS41, WDFY3, WDR48, WDR76, WFS1, ZC3HAV1L, ZCRB1, ZFP42, ZFR, ZFYVE16, ZIC4, ZMYM5, ZMYND11, ZNF23, ZNF264, ZNF273, ZNF280B, ZNF519, ZNF638, ZNHIT6
**hsa-miR-7-5p**	PTAR1, ATXN1, SATB1, STRN, ZNF704, CACNB4, CYTH3, DACH1, DPYSL2, FBXO28, GJC1, GLI3, KLF12, KLF4, PDE4D, RBMS3, TRIM33, ULK2, CKAP4, CLASP2, FAM168B, HCN1, KBTBD2, NFAT5, NOVA2, OSBPL11, OXR1, RPS6KB1, SH3GLB1, SLC25A16, SMARCD1, SNCA, SP1, VPS26A, WASF3, ZC3H4, ANKRD12, DIRAS1, GGA2, IDE, KLHL14, NDFIP2, NR4A3, NREP, NXT2, PAK2, PAN2, PLEC, PRKCB, RGS7BP, RSBN1, RSPRY1, SEMA4C, SLC5A3, SOX6, SPTY2D1, STRN3, UBXN2B, 39692, ADAM11, ADCY9, ANKFY1, ARF4, ARID2, ARID4A, ASXL1, ATP2B2, BLOC1S4, C1orf21, C1orf226, C5orf22, CACNG7, CADM3, CCDC43, CCL16, CGGBP1, CHAMP1, CHD3, CHSY3, CNN3, CNPPD1, CTSB, DDIT4, EGFR, EIF4EBP2, EPHA3, EXOSC2, FAM131B, FAM168A, FBXL7, FNDC4, FOXN3, GAL3ST3, GALNT3, GATA6, GRIN2A, GRP, HDLBP, HERPUD2, HMG20A, HPCAL4, HS3ST5, IDS, IGLON5, IPO11, IRS1, IRS2, KDM3B, KIF13A, KIF16B, LEMD3, LRRC1, LRRC59, MAFG, MAPKAP1, MEGF9, MIS12, NR2C1, OGT, PARP1, PDE4A, PFN2, PIGH, PIK3CD, PIK3R3, PLCB1, PLP2, PLXNA1, POLE4, PPP4R1, PSME3, RAB11FIP5, RAF1, RB1, RBFOX1, RNF141, RNF144A, RRAS2, RYK, SHANK2, SLC6A9, SOCS2, SPATA2, SQSTM1, SRRM3, TAB2, TCF12, TFRC, TMED9, TNK2, TRMT13, UBLCP1, UBQLN4, VDAC1, VDAC3, WDR47, XPO7, ZBTB22, ZNF395, ZNF609, ZNF805
**Genes potentially regulated by miRNAs whose levels are increased in Nuli**	
**hsa-let-7f-1**	AKAP6, COL4A3BP, CPEB2, EEA1, GTF2I, HECTD2, IKZF2, RICTOR, TGFBR3, 40057, AAK1, ADRB2, AKAP10, AKAP13, AKAP9, AP1S3, APOOL, ARAP2, ARFGEF2, ARHGAP20, ARID1B, ARID4B, ASAH1, ASAP2, ATRX, BAG2, BCL6, BHLHE40, BMPER, BMPR2, BTBD3, CALN1, CCL7, CEP41, CHD1, CLDN12, CNOT6, CNOT6L, COL19A1, COL1A2, COL4A1, CPEB3, CRISPLD1, CTTNBP2NL, DCUN1D4, DDX21, DKK3, DOCK11, DUSP6, DYNC2LI1, E2F5, EBF3, ELK4, EPHB4, ERF, FAM126B, FBXO34, FGD6, FLI1, FNDC3A, FNDC3B, FOXN2, FRMD4B, FSTL5, FUT9, GATA3, GNAQ, GOLIM4, GP1BA, GRHL3, HIP1, HIPK3, HMGXB4, HOOK3, HOXA9, HSPA14, IGF1R, INO80D, IPMK, IRX3, ITM2B, JAG2, KIAA1217, KIF3A, KLF3, KLHL2, KMT2C, LCOR, LEMD3, LIX1L, LRCH4, LRP1B, LRP6, LSM14A, LYSMD3, MAP2, MAPKAPK5, MBNL1, MBNL2, MEF2C, MEF2D, MMS22L, MON2, MYCBP2, MYCN, MYT1L, N4BP2, NAA20, NADK2, NASP, NCKAP1, NKTR, NRP1, NUP98, OLFML2B, OTX2, PAK2, PCSK2, PDE10A, PIK3C2A, PNISR, PPFIA2, PPP1R15B, PPP4R2, PRDM16, PRPF38B, PRR12, PUM2, QKI, RAB11FIP3, RAB14, RAB2A, RAB3GAP2, RAD21, RALGDS, RBM12B, RC3H1, RDX, RORA, SALL3, SATB1, SECISBP2L, SEMA3C, SEPHS1, SET, SFPQ, SH3GL3, SLAIN2, SLC2A13, SLC6A14, SLC8A1, SMARCA5, SOX9, SPOCK3, STK40, TASP1, TCF3, TET3, TFRC, TLE1, TLE4, TMEM132B, TMTC2, TOB1, TVP23B, U2SURP, UBE2B, UBE2H, UBE3C, UBR1, ULBP1, USP24, USP25, USP32, UTRN, VAV3, VCL, WAC, ZBTB39, ZMYM6, ZNF292, ZNF326, AASDH, ABI1, ACBD3, ACTL6A, ACTR3, ACTR3C, ACVR1, ACVR2B, ADAM28, AFF3, AGTR1, AHR, AKAP12, AKTIP, ALDH1L2, ALKBH8, AMMECR1, ANAPC13, ANK3, ANKRD44, ANO4, ANO5, APLF, APP, APPL1, ARHGAP12, ARHGAP44, ARHGEF28, ARHGEF33, ARID4A, ARIH1, ARNTL, ASB11, ATAD5, ATF2, ATG2B, ATL1, ATP6V1H, AUTS2, B3GNT2, BASP1, BAZ1B, BCAP29, BDNF, BLOC1S4, BMP3, BRD1, BRF2, BRIP1, BRWD3, BST1, BTAF1, BTF3L4, BTNL9, C11orf54, C11orf58, C18orf63, C1QBP, C1QTNF7, C5orf58, C6orf106, C7orf57, CAB39, CACNA1C, CACNA2D1, CADM1, CARF, CCDC102B, CCDC126, CCNA2, CCNE2, CCNY, CD38, CDC7, CDH20, CDK19, CECR2, CENPE, CEP192, CEP57, CEP76, CEPT1, CFTR, CHD3, CLASP2, CLDN1, CLDN16, CLIP1, CLOCK, CMPK1, CNNM4, CNTN1, COG7, COL11A1, COLCA2, CORO1C, CPNE2, CREBRF, CREBZF, CRY1, CTNNB1, CTR9, CTU2, CUL1, CWC22, CYP7B1, DAAM1, DACH1, DACT1, DCAF6, DCAF7, DCBLD2, DCC, DCLK3, DCLRE1B, DENND4A, DERL1, DGKH, DLL1, DLL4, DLX2, DMTF1, DNAJB5, DNAJC6, DNALI1, DNPEP, DOCK1, DOCK10, DPM1, DR1, DSG2, DVL2, E2F8, EDIL3, EFNB1, EFR3A, EGFL6, EIF1AD, EIF4A2, EIF4ENIF1, EIF4G3, ELAVL2, ELOVL2, ELOVL7, EMP2, EPS15L1, ERI1,

	ETS2, F2RL1, F3, FA2H, FAM117B, FAM126A, FAM19A4, FAM204A, FAM3C, FAM76B, FAT3, FBLN5, FBN1, FBXL3, FBXO33, FBXO43, FBXO45, FGF7, FGFR2, FLT3LG, FNBP4, FOXO1, FOXP1, FREM2, FRMD6, FRS2, FSCN1, FSHB, FYTTD1, FZD6, GABRG1, GDAP1, GDAP2, GIGYF2, GLCCI1, GLS, GNA13, GNAI3, GNAL, GNB4, GOLGA2, GPR158, GPR37, GPRIN3, GRK5, GRM3, GRM5, GSK3B, GTF2A1, GTF3C3, GULP1, HEATR5B, HECTD1, HERC2, HES1, HIC1, HIVEP2, HMGCR, HNRNPA1, HNRNPA1L2, HNRNPA2B1, HNRNPD, HS6ST2, HTR2A, ICK, ID1, ID4, IER5, IGFBP1, IMPACT, IREB2, ISM1, ITCH, ITGA6, ITGAV, ITM2C, JAG1, JAK2, JPH1, KBTBD7, KCNJ15, KDM2B, KDM6A, KIAA0408, KIAA1109, KIAA1211, KIF11, KIF21A, KIF2A, KIN, KLF12, KLF2, KLF4, KLHDC2, KPNA3, KRAS, KRBOX4, L3HYPDH, LAMP2, LGALSL, LGR4, LHFPL3, LILRA1, LIN54, LIN9, LIPG, LRRC1, LRRC32, LRRC4, LRRTM4, LTN1, LY75, LYPLAL1, MAGI1, MALT1, MAN1A1, MAP2K3, MARC1, MARCH6, MARK1, MAST4, MATN2, MBLAC2, MECOM, MED14, MED6, MEX3B, MIF4GD, MLLT10, MLLT6, MMAB, MMP20, MNX1, MPC2, MPP6, MRPL44, MSANTD2, MTF1, NAA16, NAA25, NBEA, NCOA2, NDC1, NEUROG2, NF1, NFAT5, NFYB, NGDN, NIN, NLGN1, NOL4, NPY, NPY1R, NR2F2, NR3C1, NR4A3, NR5A2, NRBF2, NRP2, NTN4, NUDT11, NUDT12, NUDT4, NUFIP2, NUP35, OLIG3, OPA1, OTUD4, OTUD6B, OXR1, PAN3, PANK3, PARD6B, PARPBP, PAXBP1, PCDH8, PDAP1, PDLIM5, PDZRN3, PEAK1, PECR, PEX5L, PFKFB3, PHACTR2, PHC3, PHIP, PHYHIPL, PI15, PIAS1, PIK3R4, PIKFYVE, PLAG1, PLCB4, PLCL2, PLEKHA6, POLR2K, POU4F2, PPM1A, PPM1D, PPM1L, PPP1R2, PPP1R21, PPP1R3F, PPP3CA, PRKAA1, PRPF8, PRRC1, PSD2, PSD3, PSIP1, PTBP3, PTCH1, PTER, PTMS, PTPRE, PUM1, RAB10, RAB11B, RAB11FIP2, RAB6B, RABGGTB, RAPH1, RASEF, RBM22, RBPJ, REV3L, RFC3, RFX1, RHOA, RHOBTB3, RHOT1, RIF1, RIMKLB, RIPPLY2, RNF138, RNF149, RNF180, RNF216, RNF24, ROCK1, RPRD1A, RPRD1B, RPS16, RSF1, RTN1, SAMD12, SBF2, SCAI, SCAMP1, SCARF1, SCN2A, SCN7A, SDC2, SEC22C, SGTB, SHOC2, SLC18A2, SLC34A2, SLC35A3, SLC39A10, SLC39A6, SLC4A5, SLC6A11, SLC6A17, SLC6A4, SMARCA2, SNAPC1, SNCAIP, SOGA3, SORBS1, SOS2, SOWAHC, SP4, SP8, SPAST, SPEN, SPESP1, SPOPL, SPRED1, SPRY1, SPRY2, SPTB, SRBD1, SRSF1, SRSF11, SRSF2, ST5, STK24, STK38L, STON2, SUN1, SV2B, SYF2, SYNGR3, TAF2, TAF4B, TAOK1, TBC1D2B, TBR1, TCERG1L, TCF20, TEAD1, TET2, TGFB3, THRB, TIGD7, TM4SF4, TMED7, TMTC4, TNS3, TRA2A, TRHDE, TRIM6, TRIM63, TRPM7, TSHZ3, TSLP, TTC30A, TTF2, TWISTNB, TXNRD3NB, UBE2Q2, UBE2W, UBFD1, UBL3, UBN1, UBN2, UBQLN1, UBTF, UBXN7, ULK1, UNC119B, USP12, USP34, VAPA, VEZF1, VMA21, WASF3, WBP2NL, WDR26, WIPI1, WNT5B, WWC1, WWC2, XPO1, YTHDC1, YTHDF3, YY1, ZBTB14, ZBTB18, ZC3H11A, ZDHHC20, ZFPM2, ZFY, ZFYVE16, ZFYVE21, ZIC1, ZMIZ1, ZMYM4, ZMYND8, ZNF236, ZNF302, ZNF367, ZNF430, ZNF501, ZNF518B, ZNF569, ZNF780A, ZNF800, ZNF827
**hsa-let-7f-2**	*AKAP6, COL4A3BP, CPEB2, EEA1, GTF2I, HECTD2, IKZF2, RICTOR, TGFBR3, 40057, AAK1, AKAP13, AKAP9, AP1S3, APOOL, ARAP2, ARFGEF2, ARHGAP20, ARID1B, ARID4B, ASAH1, ASAP2, ATRX, BAG2, BCL6, BHLHE40, BMPER, BMPR2, BTBD3, CALN1, CEP41, COL19A1, CRISPLD1, CTTNBP2NL, DCUN1D4, DDX21, DKK3, DOCK11, DUSP6, DYNC2LI1, E2F6, EBF3, ELK4, EPHB4, ERF, FAM126B, FBXO34, FLI1, FOXN2, FSTL5, FUT9, GATA3, GNAQ, GOLIM4, GP1BA, GRHL3, HAS2, HIPK3, HMGXB4, HOOK3, HOXA9, HSPA14, INO80D, IPMK, IRX3, ITM2B, JAG2, KIAA1217, KIF3A, KLF3, KLHL2, KMT2C, LCOR, LEMD3, LIX1L, LRCH4, LRP1B, LRP6, LSM14A, LYSMD3, MAP2, MAPK8, MAPKAPK5, MBNL1, MBNL2, MEF2D, MMS22L, MON2, MYCBP2, MYT1L, N4BP2, NAA20, NADK2, NASP, NCKAP1, NKTR, NRP1, NUP98, OLFML2B, OTX2, PAK2, PCDH17, PCSK2, PDE10A, PIK3C2A, PLXNC1, PNISR, PPFIA2, PPP1R16B, PPP4R2, PRDM16, PRR12, PUM2, QKI, RAB11FIP3, RAB14, RAB2A, RAB3GAP2, RAD21, RALGDS, RANBP2, RBM12B, RBM27, RC3H1, RORA, SATB1, SECISBP2L, SEMA3C, SEPHS1, SET, SFPQ, SH3GL3, SLAIN2, SLC2A13, SLC6A14, SLC8A1, SMARCA5, SOX9, SPOCK3, STARD13, TASP1, TCF3, TFRC, TLE1, TLE4, TMEM132B, TMTC2, TOB1, TVP23B, U2SURP, UBE2B, UBE2H, UBE3C, UBR1, ULBP1, USP25, USP32, VCL, WAC, ZMYM6, ZNF292, ZNF326, ABL1, ACSL3, ADAMTS5, ADARB2, ANKS1A, ANP32A, ANXA1, ARAP1, AREG, ART3, ASXL1, ATP7A, ATXN3, AXIN2, BACH2, BAZ1A, BBX, BEND7, BEX4, BMP7, BRINP1, C18orf25, C1orf52, CAV2, CBFA2T2, CBX3, CCDC73, CCDC91, CDKL3, CEP70, CFL2, CNR1, CNTN3, CNTN6, CPD, CREB5, CREBBP, CSNK1G3, CSPP1, CXXC4, CYP26B1, DAGLA, DAZ1, DAZ2, DAZ3, DAZ4, DCP2, DEPDC7, DLX6, DNAJB4, DOCK3, DPP8, DST, EDN2, EFNA1, EGLN1, ENAM, ENC1, ENPP3, EPG5, EPN2, ERCC3, EYA1, EYA4, EYS, FAM221A, FERMT2, FGF13, FJX1, FOXE1, FOXF1, FOXO3, FRMPD4, GABRA1, GABRA4, GABRB2, GCC2, GOLPH3L, GPR18, GPR63, GRASP, GRIK4, GRIP1, GRM1, HMGB3, HNRNPDL, HOXA10, HOXC6, HSPE1-MOB4, HVCN1, IAPP, INPP5B, INSM1, IQGAP1, IQGAP2, IRF2BP2, IRF5, IRX1, IRX5, ITGA4, KCNG3, KHDRBS3, KIAA0319, KIAA1191, LANCL2, LGR5, LHX2, LMO3, MAGI3, MAP2K4, MAP3K5, MBNL3, MDM1, MED13L, MED23, MEOX2, MOB4, MPZ, MS4A13, MTFR1, MTMR12, MTOR, MYO1E, NAA15, NOTO, NRIP1, NUP133, NUP153, NUP160, NXT2, OTUD1, PAH, PCDH20, PCLO, PCM1, PDCD10, PDE11A, PDE7A, PHF12, PHF20L1, PHF3, PLEKHG1, PLSCR1, POLI, PPP2CB, PRR14L, PSME4, PTGER3, PTGFR, PTPN3, RAB18, RAP1B, RAPGEF5, RASA1, RBL2, RBPMS, RECK, RGS4, RNF182, RNF220, ROBO2, RRAS, SALL1, SAMD4A, SASH1, SEC11C, SERINC3, SETD9, SFMBT2, SFSWAP, SGCD, SGCG, SLC17A6, SLC1A2, SLC22A10, SLC30A5, SLC35E1, SLC7A11, SMARCA4, SMIM8, SMOC1, SNX3, SOCS7, SORT1, SPINK5, SSBP2, STRN4, STXBP1, STYX, TCF7L2, TEK, TENM1, TGDS, TIMP3, TM2D1, TMCC1, TMEM209, TMF1, TNFRSF11B, TOR1AIP2, TP53INP1, TRAM1, TRAPPC4, TRIM13, TRIM61, TRIO, TRMT1L, TSPAN12, TXLNG, UBE2O, USP7, VCAM1, VKORC1L1, VNN1, WDR72, WDR82, WT1, XIAP, ZC2HC1A, ZCCHC24, ZFX, ZIK1, ZNF441, ZNF570, ZNF619, ZNF649, ZNF830, ZNRF3, ZSWIM6*

**hsa-let-7f-5p**	AKAP6, COL4A3BP, CPEB2, EEA1, GTF2I, HECTD2, IKZF2, RICTOR, TGFBR3, ADRB2, CCL7, CLDN12, CNOT6L, COL1A2, COL4A1, CPEB3, E2F5, E2F6, FGD6, FNDC3A, FNDC3B, FRMD4B, HAS2, HIP1, IGF1R, MAPK8, MEF2C, MYCN, PLXNC1, PPP1R15B, PPP1R16B, PRPF38B, RANBP2, RDX, SALL3, STARD13, STK40, TET3, USP24, UTRN, VAV3, ZBTB39, ZNF689, 38200, ABCB9, ABCC5, ABHD17C, ABL2, ACER2, ACSL6, ACVR1C, ACVR2A, ADAMTS15, ADAMTS8, ADRB3, AEN, AGAP1, AHCTF1, AMOT, AMT, AP1S1, APBB3, ARG2, ARHGAP28, ARHGEF38, ARID3A, ARID3B, ARL5A, ASAP1, ATP2A2, ATP8B4, B3GNT7, BACH1, BEGAIN, BEND4, BIN3, BSN, BZW1, C14orf28, C15orf39, C19orf47, C20orf194, C5orf51, C8orf58, CADM2, CASP3, CBX5, CCDC71L, CCND2, CCNJ, CCR7, CDC25A, CDC34, CDKN1A, CEP135, CERCAM, CHD4, CLCN5, CLP1, CNTRL, COIL, COL27A1, COL3A1, COL4A2, COL4A6, COL5A2, CPA4, CPEB1, DCAF15, DCUN1D2, DDI2, DDX19A, DDX19B, DHX57, DLC1, DLST, DMD, DNA2, DNAJA2, DNAJC1, DPH3, DTX2, DTX4, DUSP1, DUSP22, DVL3, E2F2, EDEM3, EDN1, EEF2K, EFHD2, EIF4G2, ELF4, ENTPD7, EPHA4, ERCC4, ERCC6, FAM104A, FAM135A, FAM189A1, FAM214B, FASLG, FAXC, FGF11, FIGN, FNIP1, FNIP2, FRAS1, FZD3, FZD4, GABBR2, GALC, GALNT1, GALNT2, GAN, GAS7, GATM, GCNT4, GDF6, GJC1, GNG5, GNPTAB, GOLT1B, GPATCH2, GPCPD1, GPR26, GXYLT1, GYG2, HAND1, HDLBP, HDX, HIC2, HIF1AN, HMGA2, HOOK1, HOXA1, HOXD1, HTR1E, IGDCC3, IGDCC4, IGF2BP1, IGF2BP2, IGF2BP3, IL13, IMPG2, INSR, IQCB1, IRS2, ITGB3, KCNC2, KCNJ11, KCTD17, KCTD21, KDM3A, KIAA0930, KIAA1958, KLF8, KLF9, KLHDC8B, KLHL31, LBR, LEPROTL1, LIMD2, LIN28A, LIN28B, LINGO1, LIPH, LIPT2, LPGAT1, LRIG2, LRIG3, MAP3K1, MAP3K9, MAP4K3, MAPK6, MARS2, MBD2, MDM4, MED28, MED8, MEIS2, MIB1, MRS2, MTDH, NAP1L1, NEK3, NGF, NHLRC3, NIPAL4, NME4, NME6, NPEPL1, NR6A1, NRAS, NYNRIN, ONECUT2, OSBPL3, OSMR, P4HA2, PALD1, PAPPA, PARP8, PBX1, PBX2, PBX3, PCDH19, PCGF3, PDE12, PDP2, PDPR, PEG10, PEX11B, PGRMC1, PIGA, PIK3IP1, PLA2G3, PLAGL2, PLEKHA8, PLEKHO1, PLXND1, POGLUT1, POGZ, POLR3D, PRKAR2A, PRLR, PRRX1, PRSS22, PTAFR, PTPRD, PXDN, PXT1, RAB11FIP4, RAB8B, RALB, RASGRP1, RBFOX2, RFX6, RGS16, RNF165, RNF20, RSPO2, RUFY3, SALL4, SCD, SCN11A, SCN4B, SDK1, SEMA4C, SEMA4G, SENP2, SENP5, SESTD1, SFMBT1, SKIL, SLC10A7, SLC16A9, SLC20A1, SLC22A23, SLC25A27, SLC2A12, SLC30A4, SLC35D2, SLC38A9, SLC5A6, SLC5A9, SMARCAD1, SMC1A, SMIM3, SNX16, SNX30, SOCS4, SPRYD4, SRD5A3, SRGAP1, STARD3NL, STARD9, STRBP, STX3, SUB1, TAF9B, TBKBP1, TECPR2, TGFBR1, THOC2, THRSP, TMC7, TMEM167A, TMEM234, TMEM65, TMOD2, TMPPE, TMPRSS2, TNFSF9, TRANK1, TRIM41, TRIM67, TRIM71, TSPEAR, TTLL4, TXLNA, UGCG, UHRF2, USP38, USP44, WDR37, WNT9B, XK, XKR8, XRN1, XYLT1, YOD1, YPEL2, ZBTB5, ZBTB8B, ZFYVE26, ZNF275, ZNF280B, ZNF322, ZNF512B, ZNF516, ZNF583, ZNF644, ZNF710, ZNF784, ZSWIM5
**hsa-miR-339-5p**	AKAP10, CHD1, CNOT6, PCDH17, RBM27, ZNF689, ALKBH1, BACE1, BCL2L11, BEND3, C6orf223, CASD1, CDK14, KIF1B, KLF11, KMT2A, LHX6, MAPRE1, PLEKHH1, SERTAD4, SHISA7, STOX2, TLE3, TMEM119, UBE3A, UNC5C
**Genes potentially regulated by miRNAs whose levels are decreased in NuLi**	
**hsa-mir-146a**	ATXN1, ABL2, ARID4A, ARL8B, ASXL3, BNIP2, C17orf75, C6orf62, CACUL1, CAMK1D, CASP7, CCM2, ETNK1, FAM126B, FOXC1, ILF3, MICU3, NEK7, NUFIP2, PLCXD3, SIX4, SLC16A6, SLC38A1, SORT1, TBC1D5, USH2A, ZEB1, ZFX, ZNF395, ACKR3, ACTN4, ADAMTS17, ADNP2, AFAP1, AHCYL1, ARHGEF12, ATG13, ATP6V1C2, AVPR1A, BMP2, BNC2, C20orf197, C5orf63, CAND1, CD164, CD207, CDCA7, CDKN2AIP, CENPN, CHD2, CLCN6, CLEC6A, CNR2, CORO1C, CPLX2, DIRC1, DIXDC1, DMRT3, DTWD1, DUSP13, EIF4E3, ERC1, ERLIN1, ETS2, EVA1A, FANCL, FOXJ2, FOXN2, FZD4, GCC2, GFRA1, HABP2, HIC2, HIF1A, HMGCR, HOXC4, HSPD1, IDI2, IGF2R, IGSF3, IKZF1, IL17RD, IRGQ, ITGB7, ITGB8, KCNC2, KDELR3, KDM5A, KIDINS220, KLF8, KPNA1, LCOR, LGI2, LHFPL5, LIN28B, LMNB2, LMO4, LRP8, LYN, MAP3K2, MARCH6, MASP1, MBLAC2, MEA1, MED14, MEF2C, MEOX1, MTRNR2L4, MTX3, MYL12A, NEUROD4, NFAT5, NFYA, NHLH2, NNT, NPAS3, NRCAM, NSUN7, NUBP1, ONECUT2, OSBPL3, PAN3, PCDH17, PCSK5, PDS5A, PMEPA1, PPP1R3B, PRR9, PTPDC1, PTPN12, PTPN14, RAG1, RASSF8, RC3H1, RFPL4B, RIOK3, RRS1, RTN4, RUNX2, SERBP1, SERTAD2, SGCG, SH3BGRL2, SHISA6, SLC16A1, SLC24A4, SLC30A4, SLC44A1, SMCO4, SNAI2, SNAP23, SNAP91, SNX27, SPRED1, SPRTN, SPRY3, SPRYD7, STRADA, STXBP6, SV2B, SV2C, TAF11, TAF1B, TAOK1, TBC1D4, TFE3, TMEM154, TMEM232, TMEM59, TMEM87B, TNKS, TRABD, TRAF3IP1, TRDMT1, TRIM34, TRIM36, TRIM39, TRIM66, TRIM6-TRIM34, TXNIP, UBFD1, UNC13A, VAPB, VAV3, WARS2, WDR7, WDR78, WWTR1, XPO4, YTHDF2, ZBED6CL, ZC2HC1C, ZEB2, ZFP1, ZFYVE26, ZHX3, ZKSCAN1, ZNF12, ZNF140, ZNF236, ZNF250, ZNF322, ZNF330, ZNF37A, ZNF441, ZNF709, ZNF71
**hsa-miR-146a-5p**	ABL2, ERBB4, SEC23IP, SORT1, ZBTB2, ZNF652, APPL1, BCORL1, CARD10, CD80, DCAF12, DDHD1, EIF4G2, ESYT2, FBXW2, FLOT2, FZD1, GALNT10, HIPK3, HNRNPD, IRAK1, LRP2, MED1, MMP16, NOVA1, NUMB, PPP1R11, PTPRA, RARB, SEMA3G, SIAH2, SLC10A3, STRBP, TDRKH, TMEM120B, TRAF6, VASN, WWC2, ZFYVE1, ZNF367
**hsa-miR-181a-2-3p**	ARID4A, C17orf75, C2orf69, ILF3, SOX6, TBC1D12, TBC1D5, TTC7B, ZFX, ACVR2B, ADIPOR2, ALG14, ANTXR2, ANXA6, ARID2, ARNTL2, ATXN2, BASP1, BDP1, BLOC1S5, C11orf58, CBR4, CCNC, CEBPZ, CNOT6, COL25A1, CRK, CUL4B, DAZ1, DAZ2, DAZ3, DAZ4, DAZL, DGKG, DISC1, DYRK2, EEF1A1, EXOSC1, FAM122A, FAM227B, FBXO48, FCHO2, FLI1, GARS, GNG4, GPM6B, GRIA1, HEG1, HMGB3, HTR2C, IGF1R, KATNAL1, KLHL15, KLHL3, KPNA6, KRAS, LAMTOR3, MANBA, MAPK8, MBNL1, METTL8, MMD, MZT1, NAMPT, NETO2, NIPBL, NPR3, NRAS, NRP1,

	OGFOD1, OSTC, PELI2, PGAM1, PHIP, PIK3C2A, PLAC8L1, PLLP, PPP1R10, PPP3R1, PPP4R2, PRDM6, PTGES2, QRFPR, QSOX2, RAB9B, RNF121, RNF135, RNF32, RRAGD, SALL1, SCLY, SEL1L, SLC36A1, SLC9A5, SLX4IP, SORBS1, STEAP2, STXBP1, SULF1, SYT1, TFAP2D, TMEM173, TXNDC5, UBLCP1, WDR38, WIPI2, ZBTB20, ZBTB41, ZNF827, ZPBP
**hsa-mir-23b**	*ATXN1, CAMK1D, FOXC1, MAPRE1, USH2A, ABCA1, ANKRD7, BARX2, C3orf56, CCDC97, CCNJL, CHD4, CORT, DSC1, ERC2, FAM49A, GIPC3, GOLGA6L1, GOLGA6L6, GPC6, HIRIP3, IGF2, ITGA5, KCNC4, KCNIP1, KSR1, LIPG, MRPL43, MTMR4, MYEOV, OSGEP, PPA2, PPM1N, PSD3, REG1A, RNF168, SIRPA, SSMEM1, TEC, TMEM127, TMEM140, TMEM217, VSIG1, ZMYM3*
**hsa-miR-23b-3p**	*ATXN1, ARL8B, ASXL3, BNIP2, C2orf69, C6orf62, CACUL1, CASP7, CCM2, ERBB4, ETNK1, FAM126B, MAPRE1, MICU3, NEK7, NUFIP2, PLCXD3, SEC23IP, SIX4, SLC16A6, SLC38A1, SOX6, TBC1D12, TTC7B, ZBTB2, ZEB1, ZNF395, ZNF652, ADAM23, ADNP, AKAP12, ALDH1A2, AMBRA1, ANKHD1, ANKRD50, ANO4, APAF1, ARHGAP20, ARNT, ARNT2, ASAH2B, ASF1A, ATP11B, ATP11C, ATP6V1E1, ATRN, ATXN7, ATXN7L3B, AUH, AUTS2, BACH2, BBX, BICD2, BLCAP, BNIP3L, BORA, BRWD1, BTAF1, C3orf52, C9orf170, CA2, CALCR, CALCRL, CAMSAP2, CAPRIN1, CBFA2T2, CBFA2T3, CBLB, CBLN1, CCDC71L, CCL7, CCNG1, CCNH, CCNT2, CCSAP, CDC40, CDK17, CDS2, CELF2, CEP350, CEP63, CGGBP1, CHST7, CHSY3, CHUK, CLCN3, CLDN12, CLEC1A, CNN2, CNOT6L, CNR1, COG3, COL11A2, COL4A1, COL4A4, CPEB2, CPSF4, CREBZF, CRISPLD1, CRLF3, CSE1L, CSNK1G3, CTCF, CUL3, CXCL12, DCBLD2, DCUN1D1, DHX15, DNAJC6, DOCK3, DOK6, DPP10, DTNA, EBF3, EGR3, ELAVL4, ELF2, ELF4, ELOVL3, ENC1, ENTPD5, EOMES, EPAS1, EPHB2, ESRP1, ESRRG, EXOC3L4, FAS, FBN2, FBXO32, FGD4, FGF2, FMR1, FOSB, FOXP2, FRAT2, FREM1, FRMD5, FUT4, FUT9, FZD5, G3BP2, GAP43, GAS2L3, GGNBP2, GJA1, GLCE, GNG2, GPR22, GPRC5B, GPSM1, GREM2, GRK5, GRM5, GSK3B, GXYLT1, HAS2, HDAC7, HDX, HEXIM1, HMGB2, HNF4G, HOXA1, HOXB5, HOXD10, HS6ST2, HSPA12A, HTR2A, IGSF10, IGSF8, IL12B, IL6R, INPP5A, INTS6, INTU, IPMK, IPO5, IPPK, ISCA2, JARID2, JMJD1C, KCNIP4, KCNK5, KDM4A, KDM6A, KIAA1107, KIAA1109, KLF3, KLHL28, KPNA4, LAMP1, LHFPL2, LIPH, LONRF3, LPAR1, LPGAT1, LPP, LRAT, LRIG1, LYPLA1, MAB21L2, MAGOHB, MAML2, MAP3K1, MAP3K5, MAP4K4, MAP7, MARCKS, MARCKSL1, MBTD1, MCFD2, MDFIC, MED12L, MEF2A, MEIS1, MET, MITF, MPP5, MRC1, MTF1, MTSS1, MYCT1, MYH1, MYH2, N4BP1, NAA50, NACC2, NAP1L5, NCOA1, NCOA2, NCOA6, NDFIP2, NDUFA5, NEDD4L, NEK6, NFIA, NLGN4X, NLK, NOL4, NR6A1, NRK, NRXN1, NRXN3, NUAK1, NUAK2, NUP50, NUS1, ORMDL1, OSBPL8, PAK2, PAK6, PARD6B, PAX9, PCDH19, PDE4B, PDE7A, PDIA6, PDPK1, PGRMC2, PIK3R3, PIP4K2B, PITPNC1, PKDCC, PKIA, PKNOX1, PKP4, PLEKHH2, PLXNC1, PNMA2, PNRC1, PNRC2, POM121C, POU2F1, POU4F2, PPARGC1A, PPIF, PPM1K, PPP1R12A, PPP2R5E, PRDM1, PRDM10, PRELID2, PRKCE, PROK2, PRR14L, PRRG1, PTEN, PTGER4, PTPN4, QSER1, RAB11FIP2, RAB39B, RAB8B, RAD21, RAI14, RALYL, RAP1A, RAP2B, RBM25, RBM47, RBPMS2, RCN1, RCOR1, REEP1, REPS2, RGS8, RNF150, RNF38, ROBO1, ROBO2, RORA, RPRD2, RRAS2, RTF1, RUFY2, SAFB, SAFB2, SATB1, SATB2, SCN2A, SDHD, SEC14L1, SEC24A, SEMA4B, SEMA6D, SERINC5, SESN2, SESN3, SFT2D1, SH3BGR, SH3PXD2A, SHROOM2, SIPA1L1, SLC12A2, SLC1A1, SLC4A4, SLC6A14, SLC7A1, SMS, SNX5, SOCS6, SOWAHC, SPHKAP, SPOCK1, SPOPL, SPSB4, SRPK1, SSBP2, STARD3NL, STAT5B, STK4, STON2, STRN, STT3B, STX12, SWT1, SYNJ1, SYT4, TAB3, TBC1D15, TBR1, TCF24, TET3, TGFBR2, TGIF1, TJP1, TLK1, TMED5, TMEM135, TMEM170A, TMEM33, TMEM38B, TMOD1, TMOD2, TNFAIP3, TNFAIP6, TNKS2, TNPO1, TNRC6A, TNRC6B, TNRC6C, TOP1, TOX, TOX3, TRIB1, TRIM63, TUSC2, TXLNG, TXNRD1, UBE2D1, UBE2K, UBE2O, UBE2R2, UBN2, UHMK1, UQCRFS1, USP53, VCAN, VEPH1, VGLL3, VKORC1L1, VTI1B, WBP2, WBP4, WDR37, WEE1, WHAMM, WNK1, WNK3, XIAP, YOD1, ZBTB18, ZBTB34, ZBTB43, ZBTB44, ZC3H12C, ZCCHC2, ZDBF2, ZFHX4, ZIC1, ZIC4, ZIC5, ZMYM2, ZNF267, ZNF287, ZNF292, ZNF420, ZNF469, ZNF655, ZNF793, ZNF839, ZNRF2*
**Genes potentially regulated by miRNAs whose levels are increased in CuFi and Nuli**	
**hsa-miR-1275**	*SERPINE2, G3BP2, MECP2, YIPF6, AP1G1, CTBS, GPATCH2L, KLHL28, MTMR4, NAALADL2, NUDT15, NXPH2, PALM2, PPM1E, SENP6, SLC30A7, SRGAP1, TACR1, TMEM38B, WIZ, ZBTB33, ZBTB7A, ZFP36L2, ZNF32, ABCF1, ACLY, ACTB, ADAR, AKNAD1, ANGPTL3, APLN, APLNR, ARRB1, ASB11, ASB4, ATCAY, ATP10B, BHLHE41, BRWD3, C12orf75, C19orf12, C8orf37, CA10, CACNA1C, CACNA1D, CACNA1E, CACNA1I, CADM4, CALCOCO1, CAMK1D, CASKIN2, CASP14, CBLN1, CBX6, CCDC148, CD8B, CDK18, CEBPG, CHAF1A, CITED4, CLDN11, CLEC2D, CNTNAP1, COL1A1, COPG1, CPSF7, CTNND2, CYP2C19, DDA1, DDX17, DIRAS2, DUSP8, DVL3, ELFN2, ELK1, ELOVL2, EMILIN3, ENY2, EXOC6B, EXOG, FAM155B, FAM50B, FBN1, FILIP1L, FOXA3, FOXN1, FOXP2, FOXP4, FSTL1, FTO, GAD2, GANAB, GATAD2B, GIMAP1, GNAT1, GPANK1, GPR3, GRIK2, GUCA1B, HMGA1, HMGB3, HMGXB3, HNRNPAB, HSD11B2, IGF1, IGF1R, IGF2, IGF2BP1, IGF2BP3, IL13RA1, INHBC, IP6K3, ISL1, JAZF1, KCNJ10, KCNV1, KCTD20, KRT23, KSR2, LALBA, LAPTM4B, LEFTY2, LIMK1, LPCAT3, LRRC28, LZTS3, MAP1A, MAPK4, MFN2, MKNK2, MPZ, MPZL2, MTHFR, MXD1, MYCT1, NAPA, NBN, NFASC, NFIC, NFIX, NKAPL, NOS1, NOVA2, NSD1, NUAK1, OTOR, OVOL2, P2RX7, PAX5, PCDHB11, PCDHB13, PCDHGA1, PCDHGA10, PCDHGA11, PCDHGA12, PCDHGA2, PCDHGA3, PCDHGA4, PCDHGA5, PCDHGA6, PCDHGA7, PCDHGA8, PCDHGA9, PCDHGB1, PCDHGB2, PCDHGB3, PCDHGB4, PCDHGB6, PCDHGB7, PCDHGC3, PCDHGC4, PCDHGC5, PHYH, PIGG, PINX1, PKM, PKNOX2, PLGLB1, PLGLB2, PLP1, POU3F2, POU4F1, PPP1R3C, PPP2R2D, PPT2, PRKACA,*

	PRKCA, PRPH2, PRR12, PSMC2, PTGIS, RAB11FIP5, RAB3A, RAB3B, RABGGTB, RAD52, RBMX, REG3A, RNF10, RNLS, RPAIN, RSPO2, SAMD4B, SCRT2, SDC3, SDCBP2, SDHAF1, SEC24A, SERTAD2, SETBP1, SH2B1, SHISA7, SIAH1, SIAH2, SIRT2, SLAMF8, SLC10A2, SLC25A42, SLC2A4RG, SLC39A3, SLC45A3, SLC6A17, SLC7A8, SNTB1, SNX29, SOX7, SPA17, SPAG9, SPEN, SPICE1, SPIDR, SPINT1, SPOCK1, SPRED3, SPTAN1, SSR1, SSX2IP, STIM1, STS, STX8, SUCNR1, SYNPR, SYT7, TBC1D15, TBC1D7, TCEANC2, TCP11L1, THUMPD2, TM9SF2, TMEM63B, TMIGD2, TMTC2, TNNI1, TRPC4AP, TSKU, TSPAN11, TTC14, TTC17, UBE2Z, UBTF, UNC13A, UPK2, VAMP2, VDAC2, VPS37B, VPS37D, WDFY1, WDHD1, ZDHHC15, ZFP14, ZNF131, ZNF275, ZNF444, ZNF629, ZSWIM4
**hsa-mir-132**	*WIZ, BTN3A2, CRTC1, DYNC1LI2, FSTL3, IRX2, SENP3*
**hsa-miR-132-3p**	***ACVR2B, AEBP2, NLK, ZBTB20, CBLL1, ETNK1, KDM5A, LIN28B, NOVA1, RB1, ARID1B, C8orf44-SGK3, CACNG2, CALU, CCDC88A, DAAM1, FEM1C, GDF5, HBEGF, MTMR10, NTNG1, OSBPL8, RASA1, RGS7BP, SGK3, SIRT1, SIX4, SRGAP1, TMEM106B, TMEM178B, USP38, ZBTB18, ZNF516, ZNF652, 37104, ADAMTS5, ADCY3, AMD1, ARID2, ATXN1, AZIN1, BOLL, BRI3, BRWD1, CAMSAP2, CC2D1B, CCDC71L, CDK19, CELSR3, CHD1, CLMN, CSDE1, DAZAP2, DCC, DCUN1D4, DPYSL3, DUSP9, DYRK2, E2F5, ELMSAN1, EP300, FAM167A, FAM91A1, FBXL20, FOXO3, GMFB, GPD2, GRM3, GTF2H1, H2AFZ, HHIP, KCMF1, KCNA6, KCNK2, KCNN3, KLF7, L3MBTL3, LEMD3, LSM11, MAP3K3, MAPK1, MED9, MEF2A, MIA3, MUC13, MYCBP2, NACC2, NFATC2, NMNAT2, NREP, PAM, PCDH10, PPM1G, PSMD12, PTBP2, PYURF, RAD54L2, RPP14, SALL1, SAP30L, SCN3A, SEC16A, SEMA6A, SERP1, SETD5, SKAP2, SLC25A28, SLC30A6, SLC6A1, SOX11, SOX5, SPPL3, SPRED1, SS18, STX16, TIMM9, TJAP1, TLN2, USP9X, ZNF521***
**hsa-miR-199a-3p**	*ACVR2B, AEBP2, CREBRF, ITGA8, NLK, RFX3, SERPINE2, ADD3, APLP2, ARL15, ATAD1, BCAR3, CBLL1, CPEB4, CTNNA2, EMC1, EPG5, ERBB4, ETNK1, FGF7, G3BP2, ITGA3, ITGA6, ITGB8, KDM5A, LIN28B, LRP2, MAP3K2, MECP2, NAA25, NOVA1, PHLPP2, PLCB1, PPP4R2, RAP2A, RB1, RBM47, SEMA3A, SH3GLB1, SLC24A2, TAOK1, VLDLR, ZHX1, ACVR2A, ADAM10, ADAMTS3, ADAMTSL3, ADRB1, AK4, ALX4, ANKRD44, ARHGEF3, ATRX, C2orf49, C9orf40, CBLB, CCDC85C, CD2AP, CDK17, CDK7, CELSR2, CEP85L, CHAD, CHSY3, COL12A1, CSRP2, CXADR, CYB5R4, DCBLD2, DEPDC1B, DNMT3A, EBF1, ESRP1, FAM199X, FBXW11, FN1, FUBP1, FXR1, GNA12, HIC2, IFFO2, ITPK1, KATNBL1, KDM3A, KDM6A, KIAA0319L, KLF13, KLHL3, LLGL2, LPAR4, LRRC1, MAP3K4, MAP3K5, MCFD2, MED12L, MPP7, MVB12B, NACC1, NEDD4, NET1, NID2, PAK4, PAWR, PDE4B, PIK3CB, PLAG1, PLEKHH1, PNRC1, PON2, PPP2R5E, PSD2, PTPN3, PTPRZ1, QKI, RAPH1, RPS6KA6, RUNX1, SCD, SDC2, SLC20A2, SLC39A10, SLITRK6, SP1, TAB2, TGIF2, TMEM62, TPPP, TUBGCP3, VAMP3, WDR47, ZNF217*
**hsa-mir-199b**	***ACVR2B, CREBRF, ITGA8, NLK, RFX3, ZBTB20, ADD3, ITGA3, RBM47, YIPF6, ABCA1, ANK3, AP1G1, BICC1, CACNB2, CACUL1, ETS1, FAM126B, FER, FLRT3, HAPLN1, KPNA4, MARCH7, NTNG1, PPARGC1A, RAD23B, SIRT1, ZNF516, ZNF704, ABHD17C, AFTPH, AKAP1, ARF6, ARHGAP12, ARHGAP21, ASRGL1, ATG14, ATP13A2, ATXN7, AUTS2, BCAM, BEND3, BTRC, CCDC120, CCDC43, CCDC88C, CCNJ, CCNL1, CDCA7L, CECR2, CELF2, CELSR1, CLCN3, CLIP1, COL5A3, CRYBG3, CSDC2, DDR1, DENND6A, ECE1, EIF5B, FAM222B, FZD4, FZD6, GCNT2, GIT1, GNG5, GPR63, GPR89A, GPR89B, GPRC5A, GSK3B, HIF1A, HMCN1, HSPA12A, HSPA5, INO80D, IPO8, KIAA1109, KLHL29, LIN7C, M6PR, MAB21L1, MAP3K11, MARCH8, MGAT3, MGAT4B, MICAL3, MPP5, MYRF, NAA40, NCSTN, NFIL3, NINL, NPAS2, NSG1, PAN3, PAX3, PAXBP1, PDE4D, PDPN, PI4KA, PLXNA2, PLXND1, PPFIBP1, RALGAPA1, RASSF2, RASSF3, RBM24, RBPMS, RGMA, RNF11, RNF38, SACS, SERPINE1, SHOC2, SLC24A3, SLC25A23, SMARCD1, SORCS3, SOS2, SULF1, SUN1, TAB3, TAF9B, TGFB2, TMEM135, TMEM245, TSPAN6, TST, UBAP1, USP31, VPS26A, WDR76, WDTC1, ZFYVE27, ZNF329, ZNF439, ZNF544, ZNF579, ZNF584, ZNF776***
**hsa-miR-199b-3p**	***ACVR2B, AEBP2, CREBRF, ITGA8, NLK, RFX3, SERPINE2, ADD3, APLP2, ARL15, ATAD1, BCAR3, CBLL1, CPEB4, CTNNA2, EMC1, EPG5, ERBB4, ETNK1, FGF7, G3BP2, ITGA3, ITGA6, ITGB8, KDM5A, LIN28B, LRP2, MAP3K2, MECP2, NAA25, NOVA1, PHLPP2, PLCB1, PPP4R2, RAP2A, RB1, RBM47, SEMA3A, SH3GLB1, SLC24A2, TAOK1, VLDLR, ZHX1, ACVR2A, ADAM10, ADAMTS3, ADAMTSL3, ADRB1, AK4, ALX4, ANKRD44, ARHGEF3, ATRX, C2orf49, C9orf40, CBLB, CCDC85C, CD2AP, CDK17, CDK7, CELSR2, CEP85L, CHAD, CHSY3, COL12A1, CSRP2, CXADR, CYB5R4, DCBLD2, DEPDC1B, DNMT3A, EBF1, ESRP1, FAM199X, FBXW11, FN1, FUBP1, FXR1, GNA12, HIC2, IFFO2, ITPK1, KATNBL1, KDM3A, KDM6A, KIAA0319L, KLF13, KLHL3, LLGL2, LPAR4, LRRC1, MAP3K4, MAP3K5, MCFD2, MED12L, MPP7, MVB12B, NACC1, NEDD4, NET1, NID2, PAK4, PAWR, PDE4B, PIK3CB, PLAG1, PLEKHH1, PNRC1, PON2, PPP2R5E, PSD2, PTPN3, PTPRZ1, QKI, RAPH1, RPS6KA6, RUNX1, SCD, SDC2, SLC20A2, SLC39A10, SLITRK6, SP1, TAB2, TGIF2, TMEM62, TPPP, TUBGCP3, VAMP3, WDR47, ZNF217***
**hsa-mir-335**	***ACVR2B, AEBP2, CREBRF, ITGA8, RFX3, SERPINE2, ZBTB20, APLP2, ARL15, ATAD1, BCAR3, CPEB4, CTNNA2, EMC1, ERBB4, FGF7, ITGA6, ITGB8, LRP2, PHLPP2, PLCB1, PPP4R2, RAP2A, SEMA3A, SH3GLB1, SLC24A2, TAOK1, YIPF6, ZHX1, AAK1, ANK3, ARFGEF2, ARID1B, BCOR, BICC1, C8orf44-SGK3, CACNG2, CACUL1, CCDC88A, CDK13, CEP350, CLVS2, CTBS, ETS1, FAM126B, FEM1C, FER, FLRT3, GPATCH2L, GPM6B, HAPLN1, IL1RAP, KLHL28, KPNA4, MARCH7, MTMR10, MTO1, NAALADL2, NUDT15, OSBPL8, OTUD4, PALM2, PHIP, PPARGC1A, PPM1E, PRKAA2, RAD23B, RGS7BP, RORA, SENP6, SGK3, SIX4, SLC30A7, TACR1, THSD7A, TMEM106B, TMEM178B, VAPA, WNT3, ZBTB18, ZBTB33, ZBTB7A, ZFP36L2, ZNF32, ZNF652, ZNF704, 41883, ABCB5, ABCD2, ABHD5, ACADSB, ACSL6, ACSM5, ACTR2, ACVR1C, ADAM32, ADAMTS1, ADAMTS8, ADI1, ADNP, ADO, AFF2, AFF4, AGFG1, AHDC1, AK7, AKAP2, AKAP8,***

	ALDH1L2, ALG10B, AMBRA1, ANGEL2, ANGPT1, ANK1, ANKRD13C, ANKRD29, ANKRD50, ANTXR2, ANXA7, AP4E1, APOOL, APPL1, ARHGAP42, ARID4B, ARL5A, ARL6IP5, ARMC1, ARMC10, ARRDC3, ASB8, ASXL3, ATE1, ATG4C, ATL2, ATP1A2, ATP1B4, ATP6V1G1, ATRNL1, B3GALNT1, B3GNT5, BAG1, BBS7, BDH2, BEND6, BID, BLOC1S5, BORA, BPTF, BRCC3, BRD1, BROX, BRSK2, C10orf88, C11orf45, C14orf119, C1GALT1, C1orf53, C6orf58, C6orf62, CACNA2D1, CACNB4, CALM1, CASR, CCDC169-SOHLH2, CCDC170, CCDC50, CCDC68, CCDC85A, CCNDBP1, CCSER1, CCT4, CCT6A, CD109, CD180, CD200, CD200R1, CD47, CDC73, CDH15, CDKL2, CDKN1B, CENPJ, CEP135, CEP170, CEP68, CERS6, CES2, CFL2, CHD2, CHD9, CHPF2, CHRNA5, CLCN5, CLDND1, CLEC12B, CLIC2, CLPX, CNOT2, CNPY2, CNTN1, CNTN3, CNTN5, CNTNAP5, CNTRL, COBLL1, COL1A2, COL4A1, COL8A1, CORO2A, CPNE3, CPT1A, CPXM2, CREBZF, CRIPT, CRISPLD1, CRISPLD2, CSMD1, CSNK1G3, CSRNP3, CTDSPL2, CUL3, CUL5, CXCL3, CYB5B, CYBRD1, CYP7A1, CYP7B1, DCUN1D1, DCUN1D5, DDHD1, DEFB132, DENND4C, DERA, DHX58, DIAPH1, DIAPH3, DIP2B, DLC1, DMRT2, DNAJB14, DNAJC3, DPYD, DSCAML1, DST, DTL, DUS4L, DUSP19, DYRK1A, ECT2L, EDEM3, EDNRA, EFNA5, EGR3, EHF, EIF1AX, EIF2A, EIF3E, EIF4B, EIF4E3, EIF5AL1, ELAVL2, ELAVL3, ELF1, ELK3, ELMO1, ELN, ELOVL7, EMB, ENPP4, EPHA4, EPHA7, ERAP1, ERC2, ERCC8, EREG, ERP44, ETF1, ETV1, EYA4, EZR, F2R, FAF2, FAM117B, FAM126A, FAM20B, FAM49A, FAM84B, FAM98A, FAT3, FEM1B, FEZF1, FGF12, FGF18, FGF9, FH, FKBP5, FMN2, FMR1, FNDC3A, FOS, FPGT, FRMD4A, FRMD4B, FRS2, FUT8, FUT9, FZD3, G6PC2, GABPA, GABRB2, GABRB3, GABRG1, GBP5, GCNT1, GGA1, GHR, GIGYF1, GLIS3, GLRX3, GLYATL3, GNB4, GNG2, GNPNAT1, GNRHR, GOLGA2, GOLGA6L4, GOLGA6L9, GOSR1, GPAM, GPN3, GPR12, GPR171, GPR180, GPR85, GPRIN3, GRID2, GRIN2A, GRIN3A, GRM5, GSKIP, GTF2H5, GUCY1A2, GXYLT1, HBS1L, HELQ, HELZ, HERC2, HERC3, HIPK1, HLCS, HOMER1, HOOK3, HOXA13, HOXD13, HSCB, HSD17B12, HSPA1B, HTR2A, HTR2C, HYPK, ID2, IFT81, IGSF3, IL16, INSIG1, IPPK, IQGAP2, ISCA1, ITGA4, ITGB6, ITGBL1, JMY, KAT2B, KAZN, KCND2, KCNH7, KCNQ5, KCNT2, KCTD4, KCTD9, KIAA0408, KIAA1143, KIN, KITLG, KLF12, KLHL13, KLHL24, KLHL9, KRAS, LARP4B, LCORL, LHFPL3, LHX9, LIFR, LMAN1, LMBR1, LMO4, LMO7, LMOD2, LPGAT1, LRP1B, LRRC2, LRRC31, LRRC40, LRRTM2, LRRTM3, LSAMP, LTN1, LYPLAL1, MAATS1, MACC1, MAGEA10, MAGEE1, MAGI3, MAGT1, MANEA, MAP2K1, MAP3K9, MAP7, MAPK9, MB21D2, MBTD1, MCAM, MCTP2, MCUR1, MDGA2, MEI4, MEX3A, MIDN, MMRN1, MOSPD2, MPC1, MRPL42, MRPS36, MSH4, MSI2, MSRB3, MTRF1L, MXI1, MXRA5, MYCN, MYO6, MYRIP, N4BP2, NAA15, NAA30, NAA35, NAALAD2, NAP1L1, NAPEPLD, NAPG, NAV1, NCEH1, NCKAP1, NCOA2, NCOA5, NCOA7, NDST3, NDUFA1, NECAB1, NECAP1, NEDD4L, NEGR1, NEK1, NETO1, NEUROG1, NFIA, NHSL1, NIPA1, NIPA2, NKAIN3, NLGN1, NOL4, NOM1, NPAS3, NPPC, NR3C1, NRCAM, NRG4, NRIP1, NSFL1C, NT5C3B, NTRK2, NUFIP2, OCIAD1, ODF2L, OGN, OLFM3, ONECUT2, OPN3, OTOA, OTUD6A, OTUD6B, OXTR, P2RY1, PA2G4, PABPC4L, PAK2, PALLD, PALM2-AKAP2, PAPPA, PARN, PARP11, PARVA, PAX6, PAX8, PBRM1, PCBP1, PCDH17, PCDH7, PCGF5, PCLO, PCM1, PCMTD1, PCSK5, PDE12, PDGFD, PDIK1L, PDS5B, PER3, PGAP1, PGM2L1, PHACTR2, PHF14, PIGV, PIK3CA, PIK3CG, PIKFYVE, PLCB4, PLCL1, PLCXD3, PLEKHM3, PLOD2, PMAIP1, PNISR, PNPT1, POGLUT1, POU2F1, PPAT, PPIL1, PPM1L, PPP3CA, PRDM4, PREX2, PRKAR2B, PRKCI, PRKG1, PRLR, PRMT2, PROSER2, PRPF40A, PRR14L, PRRG3, PRRG4, PSEN1, PSMD14, PTAR1, PTCHD4, PTEN, PTGDR, PUM2, PXDN, RAB1A, RAB30, RAB33B, RAB4A, RAB8B, RAD54B, RAPGEF1, RASGEF1A, RASSF6, RBM11, RBM12, RBM46, RBM7, RBMS2, RBMS3, RC3H1, RCSD1, REST, RFESD, RIC3, RIMKLB, RLIM, RMND5A, RND1, RNF112, RNF128, RNF138, RNF165, RNF169, RNF217, ROBO1, ROCK2, RP2, RPGRIP1L, RPRD1B, RPS6KA1, RPS6KA3, RPS6KB1, RTN3, RUFY2, RUNX1T1, RYR2, S100A14, SAMD5, SAMD8, SAR1B, SATB2, SBNO1, SBSPON, SCN3B, SCYL2, SEL1L, SEMA3C, SEMA3D, SEMA5A, SEPHS2, SEPSECS, SERPINB10, SERPINB13, SESTD1, SFMBT2, SFXN1, SGIP1, SGTB, SH3D19, SH3KBP1, SH3TC2, SHC3, SHOX2, SHROOM3, SIRT7, SLC13A1, SLC16A12, SLC17A6, SLC17A8, SLC19A2, SLC25A13, SLC25A17, SLC30A5, SLC36A4, SLC38A2, SLC4A4, SLC5A3, SLC7A11, SLC9A2, SLC9A7, SLC9C1, SLCO1A2, SMAD5, SMARCA5, SMARCE1, SMIM14, SNCA, SNX13, SNX25, SOBP, SOHLH2, SOS1, SOWAHC, SP110, SPARC, SPN, SPOP, SPTSSA, SRPK2, SRSF6, SSB, SSBP2, SST, ST18, ST3GAL6, STARD3NL, STATH, STC1, STEAP2, STIM2, STK38L, STON1, STON2, STRBP, STRN, STXBP5, STXBP5L, SUCLG2, SUZ12, SWAP70, SYT14, SYT4, TANC2, TAOK3, TBC1D3, TBC1D3C, TBC1D3F, TBC1D3G, TBC1D3H, TBC1D4, TBCK, TBL1XR1, TBX15, TCAIM, TCEAL1, TCF4, TEAD1, THAP2, THAP9, THRB, TIPARP, TLL2, TM2D3, TM9SF3, TMED4, TMED5, TMEM136, TMEM167B, TMEM200A, TMEM237, TMEM260, TMEM33, TMEM56, TMEM74, TMEM97, TMPRSS11D, TMTC1, TMTC4, TMX1, TNFSF13B, TNIP3, TOE1, TOX3, TPH2, TRA2B, TRABD2A, TRAF5, TRDMT1, TRIM23, TRIM9, TRNP1, TRPC3, TRPM8, TRUB1, TSHZ1, TSHZ2, TTBK2, TTC21B, TTC30B, TTC33, TXNRD1, UBE2D1, UBE2D4, UBE2G2, UBE2W, UBE4A, UBL3, UFL1, UGGT1, ULBP1, USP10, USP15, USP29, USP34, UTS2, UTY, VAPB, VCAN, VPS13B, VPS29, VSIG10, VTA1, WDR17, WDR7, WWTR1, XIRP2, XPR1, XRCC4, XYLB, YAF2, YTHDC1, YTHDF3, ZBTB10, ZBTB21, ZC3H11A, ZC3H14, ZC3H6, ZCCHC2, ZDHHC21, ZEB2, ZFAND3, ZFHX3, ZFP1, ZFP36L1, ZFP42, ZFP69, ZFX, ZIC3, ZMYM4, ZNF197, ZNF208, ZNF23, ZNF236, ZNF254, ZNF322, ZNF345, ZNF37A, ZNF426, ZNF430, ZNF440, ZNF449, ZNF470, ZNF480, ZNF559, ZNF578, ZNF655, ZNF681, ZNF682, ZNF697, ZNF763, ZNF786, ZNF793, ZNF845, ZNF850, ZNF92, ZNF99, ZNHIT6, ZSCAN23
**hsa-miR-335-5p**	*MAP3K2, NAA25, CALU, CDK13, CEP350, DAAM1, FBXO28, MTMR4, NXPH2, PRKAA2, RASA1, VAPA, APTX, ARGLU1, ARHGAP18, CAMKK2, CASP7, CCNF, CERS5, CHFR, CNOT7, EIF5A2, F13A1, FAM107B, FAM131B, FMN1, GJA5, GLYR1, HAND1, HOXD8, HPCAL4, KAT7, KDM2B, KDM4C, MED21, MLLT3, NCKAP5, NRXN1, PGM3, PLEKHA8, POU2F3, POU5F1, PSD3, PTPRB,*

	RBFOX2, RPRM, SECISBP2L, SEPHS1, SMARCA2, SNIP1, SORCS1, SREK1IP1, TMEM59, UBE2G1, ZMPSTE24, ZRANB1
**hsa-mir-376c**	ZBTB20, EPG5, AAK1, BCOR, CLVS2, GDF5, GPM6B, IL1RAP, MTO1, PHIP, RORA, USP38, ADH5, ALCAM, APEX1, ASB15, C5orf15, CDH6, EDNRB, EXOSC3, FAM81A, FIGN, GLS, GLT8D2, LATS2, LRP6, MACF1, NAIP, NEUROG2, NF1, NFE2L3, NFKBIA, PDSS1, PGR, PICALM, SCN1A, SLC2A2, SLC39A13, SLITRK1, TEP1, TMEM108, TMEM18, TMEM204, TTC22, WIPF3, ZKSCAN3, ZNF34, ZNF74
**hsa-miR-376c-3p**	VLDLR, ABCA1, ARFGEF2, CACNB2, FBXO28, HBEGF, OTUD4, THSD7A, TMEM38B, WNT3, ARFGEF1, ARID4A, B3GNT9, CDK12, CNIH2, CNTN4, CPEB3, DCAF7, DUSP11, EDN1, EN2, ESRRG, FRY, GLG1, GRIP1, GULP1, HNRNPK, HOXB7, HSDL1, IPO7, KANSL1L, KPNA2, LIX1L, MFAP3L, NEUROD6, NFIB, NR2F1, PAFAH1B1, PAPSS2, PDE8B, PHLDA2, PROX1, RABGEF1, RASAL2, RAVER2, RFTN2, RFX6, SASH1, SENP1, SLC25A37, SYF2, TGFBR3, TMEM255A, YTHDF1, ZMYM2, ZNF146, ZNF746, ZSWIM6
**Genes potentially regulated by miRNAs whose levels are decreased in CuFi**	
**hsa-mir-106b**	BTBD10, CLOCK, CREB5, CRYBG3, DCBLD2, EGLN3, FRS2, GABBR2, GPR137C, GXYLT1, KCNK10, MED12L, ZBTB20, ZNF236, 37104, 37500, AAK1, ABCA1, ABCG4, ABHD2, ABHD5, ABI1, ABL2, ACSL4, ADAM9, AGFG2, AHNAK, AKAP11, AKAP13, AKT3, AKTIP, AMER2, ANKFY1, ANKH, ANKIB1, ANKRD13C, ANKRD17, ANKRD29, ANKRD33B, ANKRD50, ANKRD52, ANO6, AP2B1, APCDD1, APP, ARAP2, ARHGAP1, ARHGAP12, ARHGAP26, ARHGEF10, ARHGEF11, ARHGEF18, ARHGEF28, ARHGEF3, ARID4A, ARID4B, ARMC8, ATAD2, ATG14, ATG16L1, ATG2B, ATG7, ATL3, ATP1A2, ATXN1, ATXN1L, ATXN7L1, B3GALT2, BAHD1, BAMBI, BBX, BCL11B, BCL2L11, BHLHE41, BICC1, BICD2, BMPR2, BNC2, BNIP2, BRMS1L, BRWD1, BTBD7, BTG2, BTG3, C14orf28, C2CD2, C2orf69, C3orf70, C6orf120, C7orf43, CALD1, CAPN15, CAPRIN2, CC2D1A, CCDC71L, CCND1, CCNG2, CD274, CD69, CDC23, CDC37L1, CDKN1A, CENPQ, CEP120, CEP170, CEP97, CERCAM, CFL2, CHD5, CHRM2, CLIP4, CMKLR1, CMPK1, CMTR2, CNOT6L, CNOT7, CORO2B, CPEB3, CREB1, CRK, CROT, CRY2, CSRNP3, CTSA, CYBRD1, DAB2, DDHD1, DDX5, DENND5B, DERL2, DNAJB9, DNAJC16, DNAJC27, DNAL1, DOCK4, DPYSL2, DPYSL5, DRD1, DUSP2, DUSP8, DYNC1LI2, E2F1, E2F5, EFCAB14, EGR2, EIF5A2, ELK3, ELK4, ENPP5, ENTPD4, EPHA4, EPHA5, EPHA7, ERC1, EREG, ETV1, EZH1, F3, FAM102A, FAM117B, FAM126B, FAM129A, FAM13A, FAM13C, FAM189A1, FAM199X, FAM19A1, FAM210A, FAM219B, FAM3C, FAM45A, FASTK, FAT2, FAT4, FBXL3, FBXL5, FBXO21, FBXO31, FBXO48, FCHO2, FEM1C, FGD4, FGD5, FJX1, FLT1, FNBP1L, FNDC3B, FOXJ3, FOXK2, FRMD6, FSD1L, FYCO1, FZD3, GAB1, GLIS3, GNB5, GNS, GOLGA1, GOSR1, GPR137B, GPR6, GPR63, HAS2, HAUS8, HBP1, HECTD2, HEG1, HIF1A, HLF, HPS5, HS3ST5, HSPA8, HTR2A, IGSF10, IKZF4, IL1RAP, IL6ST, IQSEC2, IRF1, IRF9, ISM2, ITCH, ITGA4, ITGB8, ITPRIPL2, JAK1, KAT2B, KATNAL1, KCNB1, KCNJ10, KIAA0513, KIAA1522, KIF23, KIF26B, KIF3B, KLF11, KLF12, KLF9, KLHL15, KLHL2, KLHL28, KMT2A, KMT2B, KPNA2, KPNA3, L3MBTL3, LAMA3, LAPTM4A, LASP1, LCOR, LDLR, LDLRAP1, LHX6, LIMA1, LIMK1, LMO3, LPGAT1, LRCH1, LRIG1, LRP8, LRRC55, LYPD6, LYST, LZIC, MAP3K2, MAP3K8, MAP3K9, MAP7, MAPK1, MAPK4, MAPRE3, MARCH8, MASTL, MCF2L, MCL1, MEX3D, MFAP3L, MFN2, MIDN, MINK1, MKNK2, MKRN1, MMP2, MMP24, MTF1, MTMR3, MYLIP, MYNN, MYT1L, NAA30, NABP1, NACC2, NAGK, NANOS1, NAPEPLD, NBEA, NCKAP5, NCOA3, NDEL1, NEDD4L, NEUROG1, NEUROG2, NFAT5, NFIB, NFIC, NHLRC3, NIN, NKIRAS1, NPAS2, NPAS3, NPAT, NPLOC4, NR2C2, NRIP3, NTN4, NTNG1, NUP35, OCRL, ORMDL3, OSBPL5, OSM, OSR1, OSTM1, OTUD4, OXR1, PAFAH1B1, PANX2, PARD6B, PBX3, PCDHA1, PCDHA10, PCDHA11, PCDHA12, PCDHA13, PCDHA2, PCDHA3, PCDHA4, PCDHA5, PCDHA6, PCDHA7, PCDHA8, PCDHAC1, PCDHAC2, PDCD1LG2, PDE3B, PDGFRA, PDLIM5, PEX5L, PFKFB3, PFKP, PGBD5, PGM2L1, PHC3, PHIP, PHLPP2, PIK3R1, PITPNA, PKD1, PKD2, PLAG1, PLAGL2, PLCB1, PLEKHA3, PLS1, PLXDC2, PLXNA1, POLR3G, PPARA, PPP1R15B, PPP1R21, PPP1R3B, PPP3R1, PPP6C, PRDM6, PRR14L, PRR15, PRR16, PRRG1, PSD, PTGDR, PTHLH, PTPDC1, PTPN21, PTPN3, PTPN4, PTPRD, PURB, PXK, RAB10, RAB11FIP1, RAB11FIP5, RAB12, RAB22A, RAB30, RAB5B, RAB8B, RACGAP1, RAP2C, RAPGEFL1, RAPH1, RASA2, RASD1, RASGRF2, RASL11B, RB1CC1, RBBP7, RBL1, RBL2, REEP3, REPS2, REST, REV3L, RGL1, RGMA, RGMB, RHOC, RLIM, RNF128, RNF217, RNF6, ROCK2, RORA, RORC, RPS6KA4, RPS6KA5, RPS6KA6, RRAGD, RRM2, RUFY2, RUNX3, S1PR1, SACS, SALL1, SALL3, SAMD12, SAMD8, SAR1B, SCAMP2, SCAMP5, SCN1A, SCN2A, SCN2B, SEMA4B, SEMA7A, SERP1, SERTAD2, SESN3, SFMBT1, SGTB, SH3BP5, SH3PXD2A, SLAIN2, SLC16A6, SLC16A9, SLC17A7, SLC22A23, SLC24A2, SLC30A7, SLC33A1, SLC40A1, SLC41A1, SLC45A4, SLC46A3, SLC4A4, SLITRK2, SLITRK3, SMAD5, SMOC1, SMOC2, SNTB2, SNX16, SNX8, SOCS6, SORL1, SOS1, SOWAHC, SOX4, SPOPL, SPRED1, SPTY2D1, SQSTM1, SRCIN1, SRGAP1, SRPK2, SS18L1, SSH1, SSX2IP, ST3GAL1, ST6GALNAC3, ST6GALNAC6, STAT3, STK11, STK17B, STK38, STRIP2, STXBP5, STYX, SUCO, SYTL4, TAGAP, TANC1, TAOK1, TAOK3, TBC1D17, TBC1D20, TBC1D8B, TBC1D9, TENM1, TET1, TET3, TGFB1I1, TGFBR2, THRA, TIAM1, TMEM100, TMEM127, TMEM167A, TMEM168, TMEM25, TMEM64, TMX3, TNFAIP1, TNFRSF21, TNKS1BP1, TNKS2, TOPORS, TP73, TPRG1L, TRIM3, TRIM36, TRIM37, TRIP10, TRIP11, TRPV6, TSG101, TSPAN9, TUSC2, TXNIP, U2SURP, UBE2Q2, UBE3C, UBXN2A, UEVLD, ULK1, UNC80, UNK, UNKL, URI1, USP24, USP28, USP3, USP31, USP32, USP46, USP6, UXS1, VANGL1, VASH2, VASP, VLDLR, VSX1, WDFY2, WDFY3, WDR37, WEE1, WFS1, WNK3, XRN1, YOD1, ZADH2, ZBTB18, ZBTB21, ZBTB33, ZBTB4, ZBTB41, ZBTB47, ZBTB7A, ZBTB8A, ZBTB9, ZC3H12C,

	ZDHHC1, ZDHHC9, ZFAND4, ZFP91, ZFPM2, ZFYVE26, ZFYVE9, ZHX2, ZNF148, ZNF2, ZNF202, ZNF25, ZNF280B, ZNF367, ZNF512B, ZNF597, ZNF652, ZNF704, ZNF800, ZNF827, ZNFX1, ZXDA
**hsa-miR-106b-3p**	*AP4S1, NR2F1*
**hsa-mir-31**	*BTBD10, CREB5, FRS2, GABBR2, GPR137C, KCNK10, MED12L, ZNF236, 40422, AIFM2, ARRDC3, AS3MT, BACH1, BCL10, C6orf62, CDH13, CHMP4B, DAPK1, DDHD2, EHBP1, ELAVL4, FCHSD2, GPCPD1, GRHL1, GRIN2A, HAUS4, HTR3E, IL15, KIAA0232, KIAA1324, KRT36, KRTAP3-3, MBD4, NAA25, NKRF, NT5DC1, PAFAH1B2, PIK3CG, PLEKHB2, POLR2K, PRDM1, PRSS23, RCOR1, RHOXF2, RHOXF2B, RXFP1, SEC31A, SHCBP1, SIPA1L2, SYBU, TDP1, TRIT1, USP9X, VAT1L, VPS13A, ZDBF2, ZMYM2, ZMYM5*
**hsa-miR-31-5p**	*CLOCK, CRYBG3, DCBLD2, EGLN3, GXYLT1, ZBTB20, AHCYL1, AK4, APBB2, ARHGEF2, ATF6, ATL2, BACH2, CAMK2D, CEP85L, CIAPIN1, CLASP2, CREG1, CSMD1, DMD, DUSP7, ELAVL1, FAM53B, FGF7, FLOT1, FNDC5, GCH1, HIF1AN, HOMER1, IDE, IL34, JAZF1, KCTD20, KCTD21, KHDRBS3, LATS2, LBH, MAP1B, MGAT1, NR5A2, NUFIP2, NUMB, NUP153, OXSR1, PAX5, PAX9, PC, PCDH8, PDZD4, PEX5, PIK3C2A, POU2F1, PPP1R9A, PRKAA2, PRKAR2A, PRKCE, PSMB11, PTGFRN, RHOBTB1, RIMS3, RNF144B, RREB1, RSBN1, SATB2, SEMA6D, SGMS1, SH2D1A, SH3BGRL2, SLC1A2, SLC2A4, SLC6A6, STARD13, STAU2, STK40, SYDE2, TACC1, TBXA2R, TENM4, TFRC, TMEM145, TMEM43, TMPRSS11F, TNS1, UACA, VAPB, VAV3, VEZT, VPS26B, VPS53, WDR5, WNK1, YWHAE, ZFP36L1*
